# Age‐specific survivorship and fecundity shape genetic diversity in marine fishes

**DOI:** 10.1002/evl3.265

**Published:** 2021-12-24

**Authors:** Pierre Barry, Thomas Broquet, Pierre‐Alexandre Gagnaire

**Affiliations:** ^1^ ISEM, Univ Montpellier, CNRS, EPHE, IRD Montpellier France; ^2^ UMR 7144, Station Biologique de Roscoff, CNRS & Sorbonne Université Roscoff France

**Keywords:** Adult lifespan, genetic diversity, life tables, marine fishes, variance in reproductive success

## Abstract

Genetic diversity varies among species due to a range of eco‐evolutionary processes that are not fully understood. The neutral theory predicts that the amount of variation in the genome sequence between different individuals of the same species should increase with its effective population size ($N_{e}$). In real populations, multiple factors that modulate the variance in reproductive success among individuals cause $N_{e}$ to differ from the total number of individuals (N). Among these, age‐specific mortality and fecundity rates are known to have a direct impact on the $N_{e}/N$ ratio. However, the extent to which vital rates account for differences in genetic diversity among species remains unknown. Here, we addressed this question by comparing genome‐wide genetic diversity across 16 marine fish species with similar geographic distributions but contrasted lifespan and age‐specific survivorship and fecundity curves. We sequenced the whole genome of 300 individuals to high coverage and assessed their genome‐wide heterozygosity with a reference‐free approach. Genetic diversity varied from 0.2% to 1.4% among species, and showed a negative correlation with adult lifespan, with a large negative effect (slope=−0.089 per additional year of lifespan) that was further increased when brooding species providing intense parental care were removed from the dataset (slope=−0.129 per additional year of lifespan). Using published vital rates for each species, we showed that the $N_{e}/N$ ratio resulting simply from life tables parameters can predict the observed differences in genetic diversity among species. Using simulations, we further found that the extent of reduction in $N_{e}/N$ with increasing adult lifespan is particularly strong under Type III survivorship curves (high juvenile and low adult mortality) and increasing fecundity with age, a typical characteristic of marine fishes. Our study highlights the importance of vital rates as key determinants of species genetic diversity levels in nature.

**Impact Summary**

Understanding how and why genetic diversity varies across species has important implications for evolutionary and conservation biology. Although genomics has vastly improved our ability to document intraspecific DNA sequence variation at the genome level, the range and determinants of genetic diversity remain partially understood. At a broad taxonomic scale in eukaryotes, the main determinants of diversity are reproductive strategies distributed along a trade‐off between the quantity and the size of offspring, which likely affect the long‐term effective population size. Long‐lived species also tend to show lower genetic diversity, a result that has however not been reported by comparative studies of genetic diversity at lower taxonomic scales. Here, we compared genetic diversity across 16 European marine fish species showing marked differences in longevity. Adult lifespan was the best predictor of genetic diversity, with genome‐wide average heterozygosity ranging from 0.2% in the black anglerfish (*Lophius budegassa*) to 1.4% in the European pilchard (*Sardina pilchardus*). Using life tables summarizing age‐specific mortality and fecundity rates for each species, we showed that the variance in lifetime reproductive success resulting from age structure, iteroparity, and overlapping generations can predict the range of observed differences in genetic diversity among marine fish species. We then used computer simulations to explore how combinations of vital rates characterizing different life histories affect the relationship between adult lifespan and genetic diversity. We found that marine fishes that display high juvenile but low adult mortality, and increasing fecundity with age, are typically expected to show reduced genetic diversity with increased adult lifespan. However, the impact of adult lifespan vanished using bird and mammal‐like vital rates. Our study shows that variance in lifetime reproductive success can have a major impact on species genetic diversity and explains why this effect varies widely across taxonomic groups.

Genetic diversity, the substrate for evolutionary change, is a key parameter for species adaptability and vulnerability in conservation and management strategies (Frankham 1995; Lande 1995; DeWoody et al. 2021). Understanding the determinants of species' genetic diversity has been, however, a long‐standing puzzle in evolutionary biology (Lewontin 1974). Advances in DNA sequencing technologies have allowed to describe the range of genetic diversity levels across eukaryote species and identify the main evolutionary processes governing that variation (Leffler et al. 2012; Romiguier et al. 2014). Yet, the extent and reasons for which life history traits, and in particular reproductive strategies, influence genetic diversity remain to be clarified (Ellegren and Galtier 2016).

The neutral theory provides a quantitative prediction for the amount of genetic variation at neutral sites (Kimura 1983). Assuming equilibrium between the introduction of new variants by mutation occurring at rate μ, and their removal by genetic drift at a rate inversely proportional to the effective population size $N_{e}$, the amount of genetic diversity (θ) of a stable randomly mating population is equal to $4N_{e}\mu$ (Kimura and Crow 1964). This quantity should basically determine the mean genome‐wide heterozygosity expected at neutral sites for any given individual in that population. However, because the neutral mutation‐drift balance can be slow to achieve, contemporary genetic diversity often keeps the signature of past demographic fluctuations rather than being entirely determined by the current population size. Therefore, genetic diversity should be well predicted by estimates of $N_{e}$ that integrate the long‐term effect of drift over the coalescent time. Unfortunately, such estimates are very difficult to produce using demographic data only.

Demographic variations set aside the most proximate determinant of $N_{e}$ is the actual number of individuals (N), also called the census population size. Comparative genomic studies in mammals and birds have shown that current species abundance correlates with the long‐term coalescence $N_{e}$, despite a potential deviation from long‐term population stability in several of the species studied (Díez‐Del‐Molino et al. 2018; Leroy et al. 2020; Peart et al. 2020). General laws in ecology, such as the negative relationship between species abundance and body size (White et al. 2007) have also been used to predict the long‐term $N_{e}$. Higher genetic diversity in small body size species was found in butterflies and Darwin's finches (Brüniche‐Olsen et al. 2019; Mackintosh et al. 2019), while in the latter genetic diversity also positively correlated with island size, another potential proxy for the long‐term $N_{e}$ (Brüniche‐Olsen et al. 2019). Surprisingly, however, genetic diversity variation across metazoans is much better explained by fecundity and propagule size than classical predictors of species abundance such as body size and geographic range (Romiguier et al. 2014). This result has been attributed to differences in extinction risk for species that have contrasted reproductive strategies. Under this hypothesis, species with low fecundity and large propagule size (K‐strategists) would be more resilient to low population size episodes compared to species with high fecundity and small propagule size (r‐stategists), which would go extinct if they reach such population sizes (Romiguier et al. 2014). By contrast, Mackintosh et al. (2019) found no effect of propagule size on genetic diversity within Papilionoidea, a superfamily showing little variation in reproductive strategy. Therefore, the major effect of the r/K gradient on genetic diversity variation across metazoa probably hides other determinants that act within smaller branches of the tree of life. In particular, how demography and evolutionary processes influence genetic variation in different taxa remains unclear.

Other factors than fluctuations in population size are known to reduce the value of $N_{e}$ relative to the census population size, impacting the $N_{e}/N$ ratio to a different extent from one species to another. These factors include unbalanced sex ratios, variance in lifetime reproductive success among individuals, age structure, kinship‐correlated survival, and some metapopulation configuration (Wright 1969; Lande and Barrowclough 1987; Falconer 1989). A potentially strong effect comes from variance in the number of offspring per parent ($V_{k}$), which reduces $N_{e}$ compared to N following $N_{e}=\frac{4N-4}{V_{k}+2}$ (Crow and Kimura 1970). Variance in reproductive success can naturally emerge from particular age‐specific demographic characteristics summarized in life tables that contain age‐specific (or stage‐specific) survival and fecundity rates (Ricklefs and Miller 1999). The impact of life tables characteristics on expected $N_{e}/N$ ratio has been the focus of a large body of theoretical and empirical works (Nunney 1991, 1996; Waples 2002, 2016a, 2016b; Waples et al. 2018). Accounting for iteroparity and overlapping generations, a meta‐analysis of vital rates in 63 species of plants and animals revealed that half of the variance in $N_{e}/N$ among species can be explained by just two life history traits: adult lifespan and age at maturity (Waples et al. 2013). Interestingly, longevity was the second most important factor explaining differences in genetic diversity across metazoans (Romiguier et al. 2014). However, there is still no attempt to evaluate the extent to which lifetime variance in reproductive success explains differences in genetic diversity between species with different life table components.

Marine fishes are good candidates to address this issue. They are expected to show a particularly high variance in reproductive success as a result of high abundance, type III survivorship curves (i.e., high juvenile mortality and low adult mortality) and increasing fecundity with age. Consequently, it has been suggested that marine fish species show a marked discrepancy between adult census size and effective population size, resulting in $N_{e}/N$ ratios potentially smaller than $10^{-3}$. The disproportionate contribution of a few lucky winners to the offspring of the next generation is sometimes referred as the “big old fat fecund female fish” (BOFFFF) effect, a variant of the “sweepstakes reproductive success” hypothesis (Hedgecock 1994; Hedrick 2005; Hedgecock and Pudovkin 2011) that is often put forward to explain low empirical estimates of effective population sizes from genetic data (Hauser and Carvalho 2008). However, subsequent theoretical work showed that low values of $N_{e}/N$ less than 0.01 can only be generated with extreme age‐structure characteristics (Waples 2016b). The real impact of lifetime variance in reproductive success on genetic diversity thus remains unclear, even in species like fish in which its impact is supposed to be strong. Contrasting results have been obtained by comparative studies in marine fishes, including negative relationship between diversity and body size (Waples 1991; Pinsky and Palumbi 2014), fecundity (Martinez et al. 2018), and overfishing (Pinsky and Palumbi 2014). However, these studies relied on few nuclear markers, that could provide inaccurate or biased estimates of genetic diversity (Väli et al. 2008). They also compared species sampled from different locations, thus, likely having different demographic histories, which could blur the relationship between species characteristics and genetic diversity (Ellegren and Galtier 2016).

Here, we compared the genome‐average heterozygosity to the life history traits and life table characteristics of 16 marine teleostean species sharing similar Atlantic and Mediterranean distributions. We estimated genetic diversity from unassembled whole‐genome reads using GenomeScope (Vurture et al. 2017) and checked the validity of these estimates with those obtained using a high‐standard reference‐based variant calling approach. Using these data, we related species genetic diversity to eight simple quantitative and qualitative life history traits. Then, we built species life tables and determined if the lifetime variance in reproductive success induced by these tables could explain observed differences in genetic diversity using an analytical and a forward‐in‐time simulation approach. Finally, we generalized our findings by exploring the influence of age‐specific survival and fecundity rates on the variance in reproductive success and ultimately genetic diversity via simulated lifetimes tables.

## Materials and Methods

### SAMPLING, DNA EXTRACTION, AND WHOLE‐GENOME SEQUENCING

**Figure 1:**
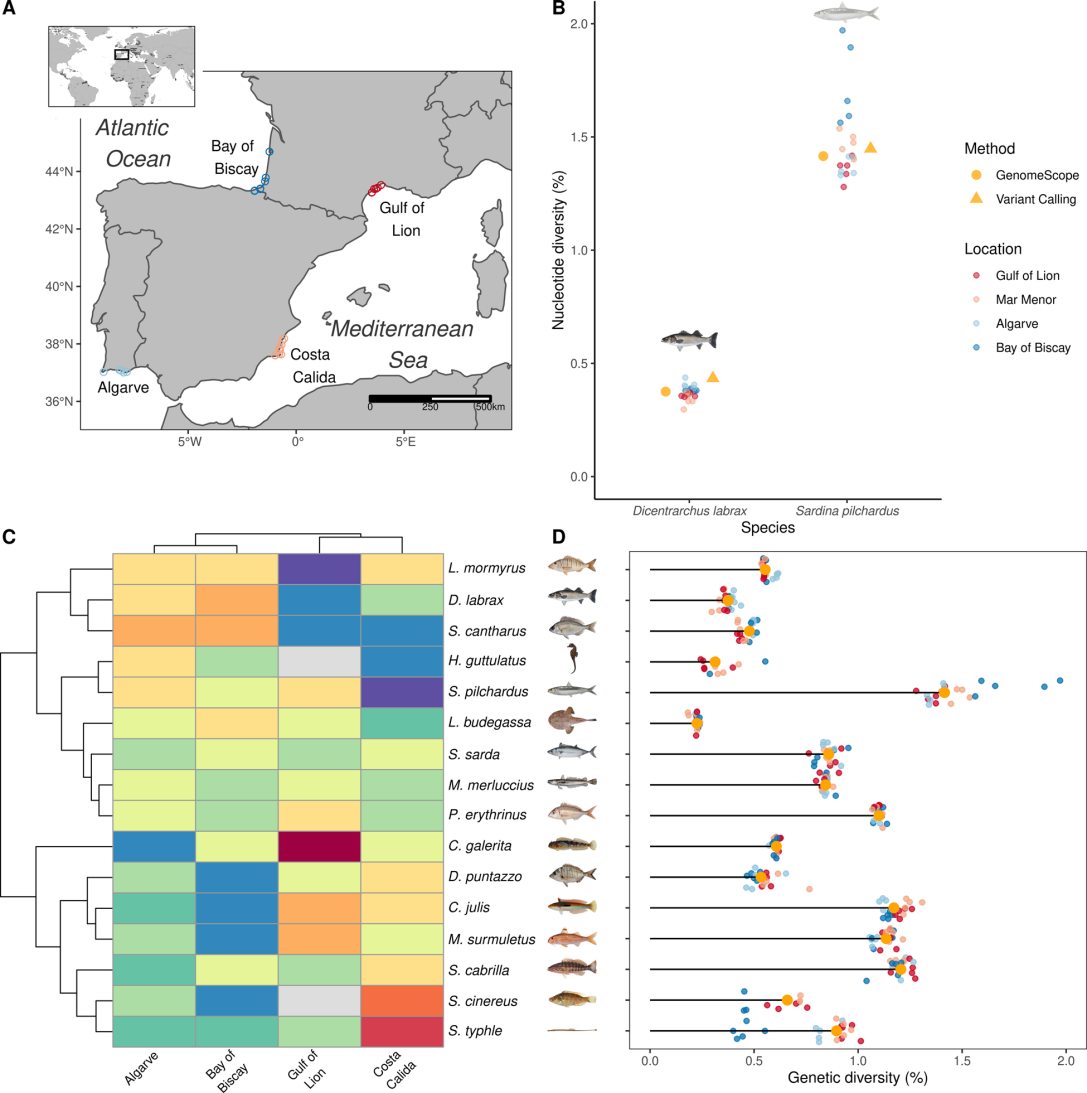
Sampling and estimation of genetic diversity in 16 marine fish species. In panels A, B, and D, the geographical origin of samples is represented by colors. Atlantic: Bay of Biscay (dark blue), Faro region in Algarve (light blue). Mediterranean: Murcia region in Costa Calida (pink), and Gulf of Lion (red). (A) Sampling map of all individuals included in this study. Each point represents the coordinates of a sample taken from one of four locations: two in the Atlantic Ocean and two in the Mediterranean Sea. (B) Genome‐wide diversity in the European pilchard (*S. pilchardus*) and European sea bass (*D. labrax*) estimated after variant calling (orange triangle) or from GenomeScope (orange dot: median; smaller dots: individual estimates) (C) Heatmap clustering showing the variance in scaled genetic diversity within species among locations. Each line represents one species, with the corresponding species name written on the right side; every column represents one location. Blue and red colors, respectively, indicate lower and higher genetic diversity within a location for a given species compared to the average species genetic diversity. (D) Individual and median genetic diversity within each species estimated with GenomeScope. Species illustrations were retrieved from Iglésias (2013) with permissions.

We sampled 16 marine teleostean fish species presenting a wide diversity of life history strategies expected to affect genetic diversity (Table 1). All these species share broadly overlapping distributions across the northeastern Atlantic and Mediterranean regions. Sampling was performed at the same four locations for all species: two in the Atlantic (the Bay of Biscay in southwestern France or northwestern Spain and the Algarve in Portugal), and two in the western Mediterranean Sea (the Costa Calida region around Mar Menor in Spain and the Gulf of Lion in France; see Fig. 1A). Individual whole‐genome sequencing libraries were prepared following the Illumina TruSeq DNA PCR‐Free Protocol and sequenced to an average depth of 20× on an Illumina NovaSeq 6000 platform by Genewiz, Inc. (USA). Raw reads were preprocessed with fastp version 0.20.0 (Chen et al. 2018) using default parameters (see Supporting Information).

**Table 1:** Life history traits and observed genetic diversity of the 16 teleostean marine species

Species	Vernacular name	*N*	Genetic diversity (%)	Body size (cm)	Trophic level	Fecundity	Propagule size (mm)	Maturity (years)	Lifespan (years)	Adult lifespan (years)	Parental care	Hermaphroditism
*Coryphoblennius galerita*	Montagu's blenny	16	0.607(±0.014)	7	2.28	NA	3.3	1.5	6	4.5	NG	–
*Coris julis*	Rainbow wrasse	20	1.172(±0.056)	27.2	3.24	169.81	0.63	1	7	6	–	PG
*Dicentrarchus labrax*	European sea bass	20	0.375(±0.031)	102.15	3.47	12,436.52	1.15	3	15	12	–	–
*Diplodus puntazzo*	Sharp‐snout seabream	19	0.533(±0.074)	49.69	3.07	277.87	0.87	2	10	8	–	RUD
*Hippocampus guttulatus*	Long‐snouted seahorse	12	0.313(±0.090)	19.8	3.5	1.21	12	0.5	5	4.5	MP	–
*Lophius budegassa*	Black anglerfish	20	0.225(±0.015)	103	4.23	2304.03	1.88	7.5	21	13.5	–	–
*Lithognathus mormyrus*	Striped seabream	20	0.553(±0.027)	37.85	3.42	214.09	0.75	2	12	10	–	PA
*Merluccius merluccius*	European hake	20	0.844(±0.025)	88.9	4.43	2294.54	1.07	3	11	8	–	–
*Mullus surmuletus*	Striped red mullet	19	1.135(±0.048)	30.18	3.46	2569.32	0.86	1.5	6	4.5	–	–
*Pagellus erythrinus*	Common pandora	19	1.100(±0.020)	36	3.46	2280.46	0.77	2	8	6	–	PG
*Serranus cabrilla*	Comber	19	1.205(±0.055)	30.8	3.68	37.97	0.91	2	6	4	–	–
*Spondyliosoma cantharus*	Black seabream	19	0.478(±0.034)	35.7	3.27	425.62	2.1	3	10	7	NG	PG
*Symphodus cinereus*	Gray wrasse	10	0.660(±0.125)	14.1	3.3	13.20	2.87	1.5	6	4.5	NG	–
*Sardina pilchardus*	European pilchard	20	1.415(±0.182)	20.35	2.94	22.89	1.64	1	5	4	–	–
*Syngnathus typhle*	Broadnosed pipefish	20	0.859(±0.047)	26.2	3.75	0.38	20	1	3	2	MP	–
*Sarda sarda*	Atlantic bonito	20	0.896(±0.208)	68.9	4.34	15,647.73	1.3	1	4	3	–	–

*Note*: For each species, the number of individuals used for the estimation of genetic diversity; observed median genetic diversity among all individuals (±standard deviation); body size (in centimeters); trophic level; fecundity (in eggs/day); propagule size (in millimeters); age at first maturity (in years), lifespan (in years), adult lifespan (in years, defined as the difference between lifespan and age at maturity), parental care behavior (−, no egg protection; NG, nest guarders; MP, male brood‐pouch), and hermaphroditism (−, no hermaphroditism; PG, protogynous; PA, protandrous, RUD, rudimentary). Detailed bibliographic references are provided in the Supporting Information.

### ESTIMATION OF GENETIC DIVERSITY

We used GenomeScope version 1.0 to estimate individual genome‐wide heterozygosity (Vurture et al. 2017). Briefly, this method uses a *k*‐mers‐based statistical approach to infer overall genome characteristics, including total haploid genome size, percentage of repeat content, and genetic diversity from unassembled short‐read sequencing data. We used jellyfish version 2.2.10 to compute the *k*‐mer profile of each individual (Marçais and Kingsford 2011). The genetic diversity of each species was determined as the median of the individual genome‐wide heterozygosity values. We chose the median instead of the mean diversity because it is less sensitive to the possible presence of individuals with nonrepresentative genetic diversity values (e.g. inbred or hybrid individuals) in our samples.

To assess the reliability of GenomeScope and detect potential systematic bias, we compared our results with high‐standard estimates of genetic diversity obtained after read alignment against available reference genomes (see details in Supporting Information). To perform this test, we used the sea bass (*Dicentrarchus labrax*) and the European pilchard (*Sardina pilchardus*), two species that represent the lower and upper limits of the range of genetic diversity in our dataset (Table 1, Fig. 1D).

### LIFE HISTORY TRAITS DATABASE

We collected seven simple quantitative variables describing various aspects of the biology and ecology of the 16 species: body size, trophic level, fecundity, propagule size, age at maturity, lifespan, and adult lifespan (Tables 1 and S4 for detailed information on bibliographic references). We used the most representative values for each species and each trait when reported traits varied among studies due to plasticity, selection, or methodology. In addition, we collected two qualitative variables describing the presence/absence of hermaphroditism and brooding behavior, as revealed by males carrying the eggs in a brood pouch (*Hippocampus guttulatus* and *Syngnathus typhle*) or nest‐guarding (*Coryphoblennius galerita*, *Symphodus cinereus*, and *Spondyliosoma cantharus*). Detailed information on data collection is available in Supporting Information.

### CONSTRUCTION OF LIFE TABLES

Life tables summarize survival rates and fecundities at each age during lifetime (Ricklefs and Miller 1999). Thus, they provide detailed information on vital rates that influence the variance in lifetime reproductive success among individuals. This tool is well designed to describe population structure from the probability of survival to a specific age at which a specific number of offspring are produced. Ideally, age‐specific survival is estimated by direct demographic measures, such as mark‐recapture. Unfortunately, direct estimates of survival were not available for the 16 studied species. We thus followed Benvenuto et al. (2017) to construct species life tables. Age‐specific mortality of species sp, $m_{sp,a}$, is a function of species body length at age a, $L_{sp,a}$, species asymptotic Von Bertalanffy length $L_{sp,inf}$, and species Von Bertalanffy growth coefficient, $K_{sp}$:

(1) $$m_{sp,a}={\left[\left(\frac{L_{sp,a}}{L_{sp,inf}}\right)\right]}^{-\frac{1}{5}}\times K_{sp}.$$

Age‐specific survival rates, $s_{sp,a}$ were then estimated as

(2) $$s_{sp,a}=e^{-m_{sp,a}}.$$

We collected age‐specific length from empirical data and estimated $L_{inf}$ and K values from age‐length data as explained in the Supporting Information Appendix, setting survival probability to zero at the maximum age (Appendix S1). When differences in age‐specific lengths between sexes were apparent in the literature, we estimated a different age‐specific survival curve for each sex. The relationship between absolute fecundity and individual length is usually well fitted with the power‐law function ($F=\alpha L^{\beta }$), although some studies also used an exponential function ($F=\alpha e^{\beta L}$) or a linear function (F=α+Lβ). We collected empirical estimates of α and β and determined age‐specific fecundity from the age‐specific length and the fecundity‐length function reported in the literature for each species. Fecundity was set to zero before the age at first maturity.

### EFFECT OF THE VARIANCE IN REPRODUCTIVE SUCCESS ON THE $N_{e}/N$ RATIO

To understand how differences in life tables drive differences in genetic diversity between species, we estimated the variance in lifetime reproductive success, $V_{k}$ and the ensuing ratio $N_{e}/N$ using the analytic framework developed in AgeNe (Waples et al. 2011). AgeNe infers $V_{k}$ using information from life tables only. Hence, the estimated variance in reproductive success estimated is only generated by interindividual differences in fecundity and survival. AgeNe assumes constant population size, stable age structure, and no heritability of survival and fecundity. We used the life tables constructed as described above and set the number of new offspring to 1000 per year. This setting is an arbitrary value that has no influence on the estimation of either $V_{k}$ or $N_{e}/N$ by AgeNe. For all species, we set an initial sex ratio of 0.5 and equal contribution of individuals of the same age (i.e., no sweepstakes reproductive success among same‐age individuals). We ran AgeNe and estimated $N_{e}/N$ for each species.

Four life tables components can generate differences in $N_{e}/N$ between species: age at maturity, age‐specific survival rates, age‐fecundity relationships and sex‐related differences in these components. To determine the role that each parameter plays in shaping levels of genetic diversity among species, we built 16 alternative life tables where the effect of each component was added one after the other, while the others were kept constant across species. Thus, in our null model, age at maturity was set at 1 year for all species, fecundity and survival did not vary with age (constant survival chosen to have 0.01% of individuals remaining at maximum age, following Waples 2016b), and there were no differences between sexes. Next, the effect of each component was tested by replacing these constant values with their biological values in species' life tables. For each of the 16 life tables thus constructed, we tested whether variation in $N_{e}/N$ explained the variation in observed genetic diversity after scaling these two variables by their maximum value. With this scaling, the correlation between $N_{e}/N$ and genetic diversity should overlap with the y=x function in cases where a decrease in $N_{e}/N$ predicts an equal decrease in genetic diversity, indicating a strong predictive power of the components included in life tables.

### FORWARD SIMULATIONS

A complementary analysis of the contribution of life table properties on genetic diversity was performed using forward simulations in SLiM version 3.3.1 (Haller and Messer 2017). Compared to the deterministic model implemented in AgeNe, the forward simulations include the stochastic variation inherent to the coalescent process and directly predict genetic diversity. Thus, they provide another approach to the problem and can lead to a more intuitive understanding of why vital rates affect $N_{e}$ over the long‐term, and ultimately genetic diversity. We simulated populations with overlapping generations, sex‐specific lifespan, and age‐ and sex‐specific fecundity and survival. We used life tables estimated as previously, and sex‐specific lifespan estimates were collected in the literature as described above. However, age at maturity was not taken into account in these forward simulations for technical reasons. Age and species‐specific fecundity were determined as previously and scaled between 0 (age 0) and 100 (maximum age) within each species. In the simulations, each individual first reproduces and then either survives to the next year or dies following a probability determined by its age and the corresponding life table. We kept population size constant and estimated the mean genetic diversity (i.e., the proportion of heterozygous sites along the locus) over the last 10,000 years of the simulation after the mutation‐drift equilibrium was reached and using 50 replicates (see Supporting Information for further information).

As previously, we evaluated the contribution of each component among 8 alternative life tables by comparing scaled observed and simulated genetic diversity.

### EVALUATING THE IMPACT OF LIFE TABLES BEYOND MARINE FISH

To generalize our understanding of the influence of life tables on genetic diversity beyond the species analyzed in this study, we simulated a wide range of age‐specific survival and fecundity curves and explored their effect on the relationship between adult lifespan and variance in reproductive success. To this end, we defined 16 theoretical species with age at first maturity and lifespan equal to that of our real species and then introduced variation in survival and fecundity curves. First, age‐specific mortality was simulated following Pinder et al. (1978):

(3) $$M\left(\text{Age},\text{Age}+1\right)=1-{\text{exp}}^{{\left(\frac{\text{Age}}{b}\right)}^{c}-{\left(\frac{\text{Age}+1}{b}\right)}^{c}},$$

where c defines the form of the survivorship curve, with c>1, c=1 and c<1 defining respectively *Type I* (e.g., mammals), *Type II* (e.g., birds), and *Type III* (e.g., fish) survival curves. We took values of c from 0.01 to 30 (Fig. 4A). Parameter b was equal to $-\frac{\text{Lifespan}}{\log{\left(0.01\right)}^{1/c}}$ to scale survivorship curves in such a way that 1% of the initial population remains at maximum age.

**Figure 2:**
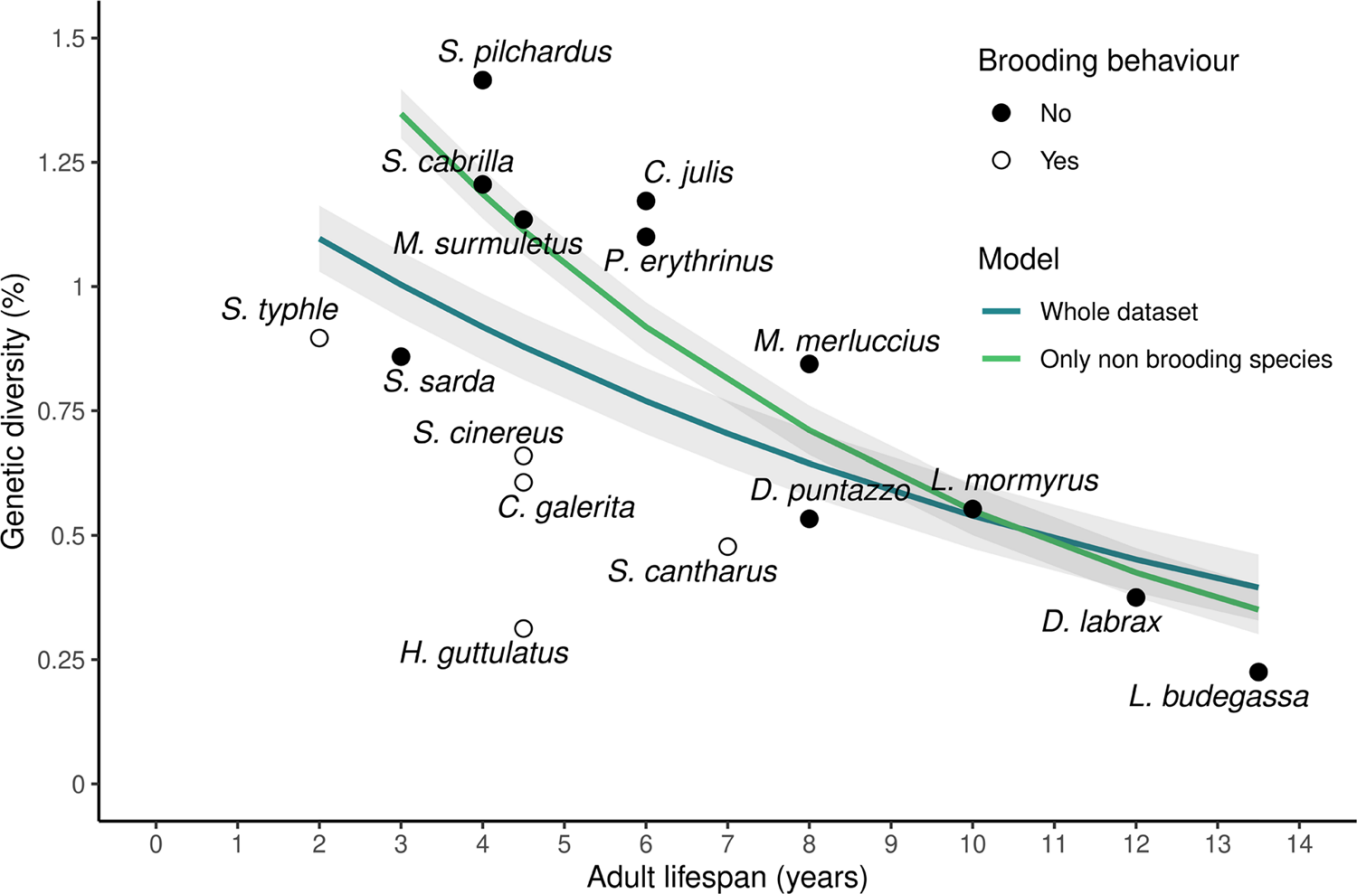
Relationship between species median genetic diversity (%) and adult lifespan, Each point represents the median of the individual genetic diversities for a given species. Adult lifespan is defined as the difference between lifespan and age at first maturity in years. Dot points and empty circles represent nonbrooding species and brooding species, respectively. Blue and green lines represent the beta regression between adult lifespan and genetic diversity considering either the whole dataset (16 species), or the 11 nonbrooding species only, respectively.

**Figure 3:**
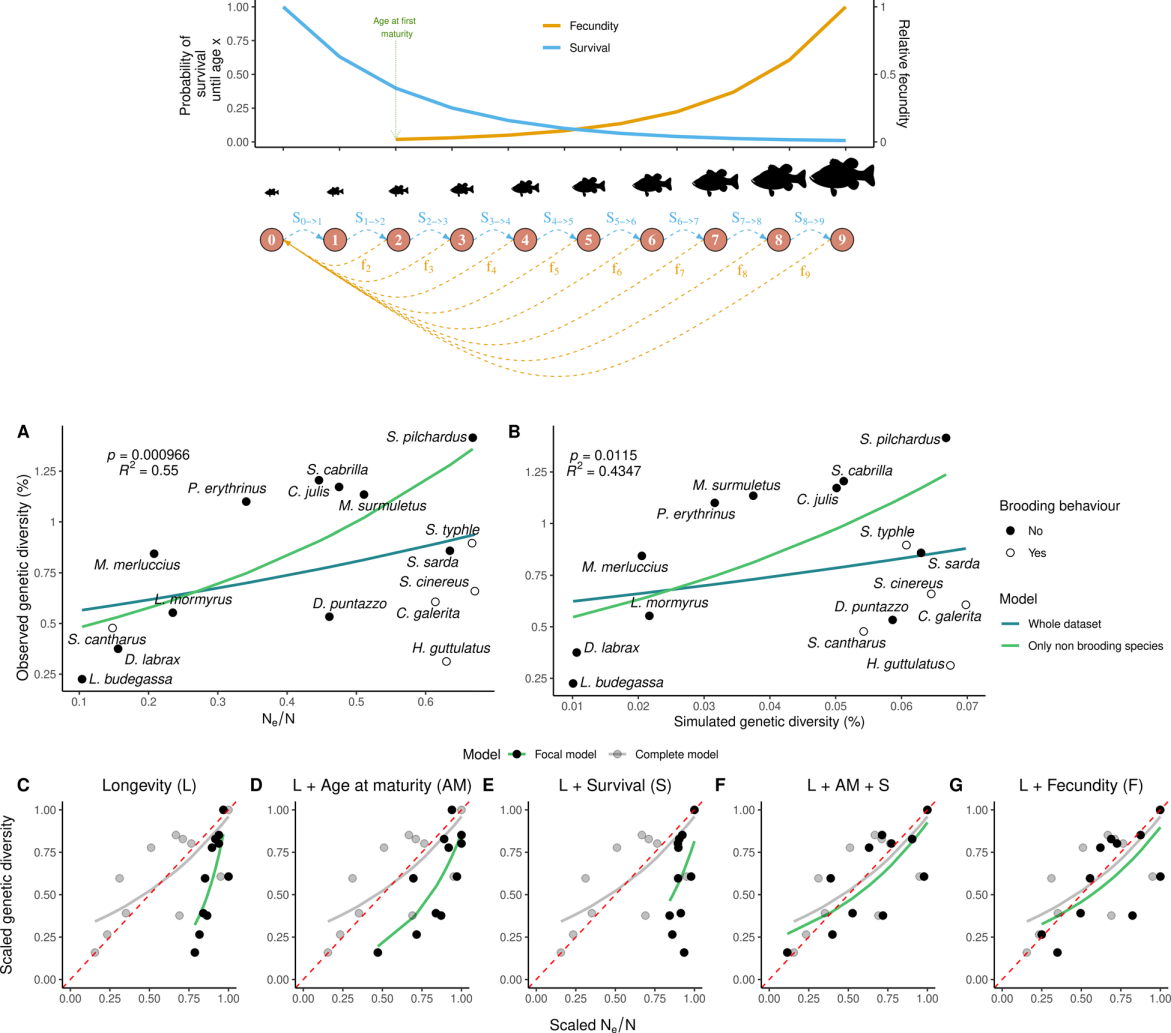
Variance in reproductive success induced by age‐specific vital rates and adult lifespan correlates with observed genetic diversity. On top, schematic illustration of age‐specific fecundity ($F_{\text{Age}}$, in orange) and survival ($S_{\text{Age}->\text{Age}+1}$, blue) for a given species. (A) and (B) represent the relationship between observed genetic diversity on the y‐axis and, respectively, $N_{e}/N$ estimated by AgeNe, and simulated genetic diversity with forward‐in‐time simulations in SLiM version 3.31 (Haller and Messer 2017), on x‐axis. Life tables containing information on age‐specific survival, fecundity and lifespan were used for the 16 species. Age at maturity was used only with AgeNe. Dot points represent nonbrooding species and empty circles, brooding species. Blue and green lines represent the beta regression between adult lifespan and genetic diversity considering the whole dataset (16 species), and the 11 nonbrooding species only, respectively. The p-value and the pseudo‐$R^{2}$ are represented on the top left for each of the two top panels for the nonbrooders model. Panels (C)–(G) represent the relationship between scaled genetic diversity and scaled $N_{e}/N$ (i.e., divided by the maximum corresponding value) for the 11 nonbrooding species. In each panel, the gray points represent scaled $N_{e}/N$ estimated from life tables including age at maturity, age‐specific fecundity and survival and sex‐specific differences (as in Panel A). Black points are scaled estimates of $N_{e}/N$ from life tables with only: (C) longevity (L); (D) longevity (L) and age at maturity (AM); (E) longevity (L) and age‐specific survival (S); (F) longevity (L), age at maturity (AM) and age‐specific survival (S); and (G) longevity (L) and age‐specific fecundity (F). Beta regression models (gray and green lines) that closely overlap the red dotted line indicate that a decrease in $N_{e}/N$ leads to a similar decrease in genetic diversity.

**Figure 4:**
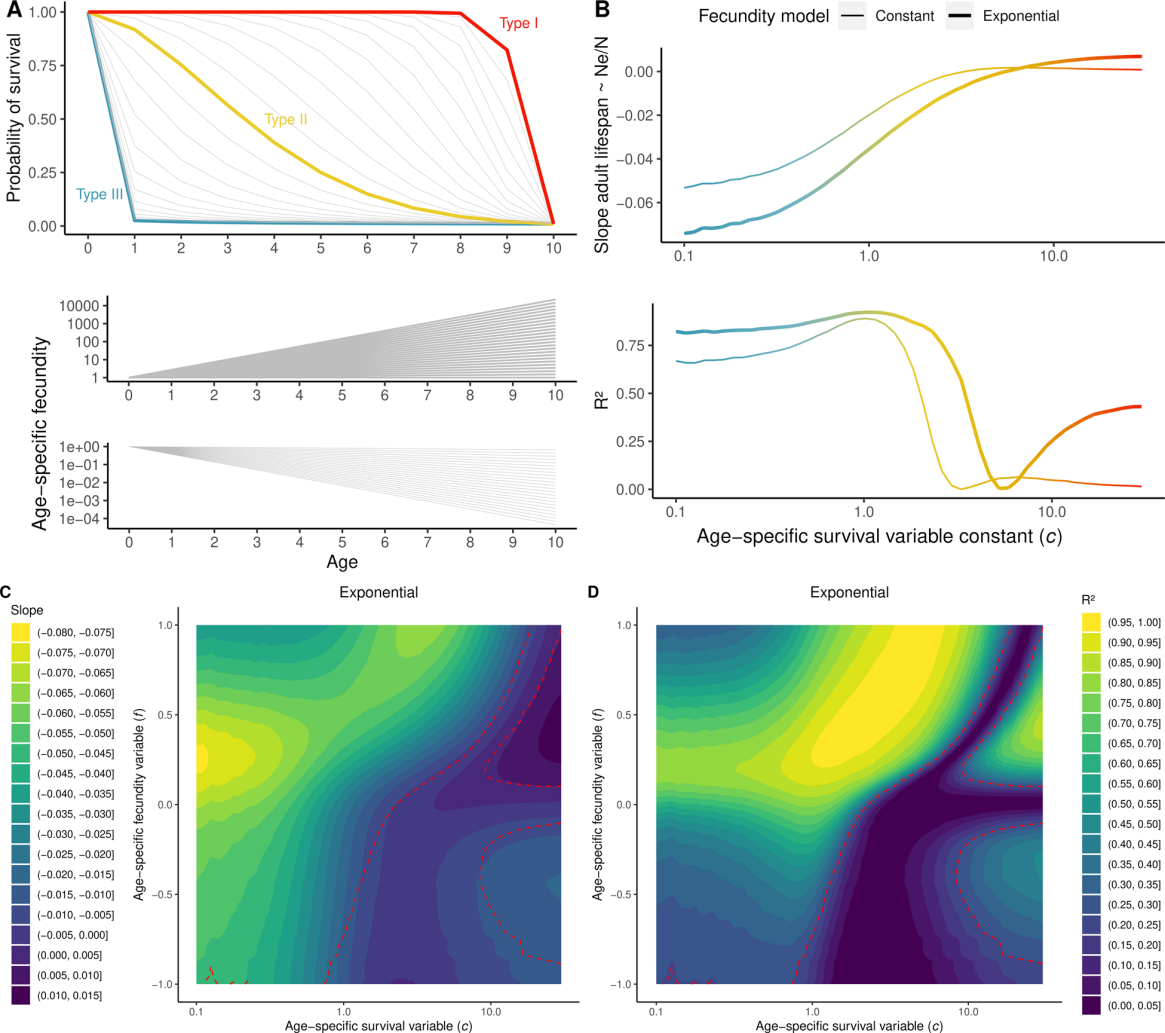
Slope of the linear model between adult lifespan and $N_{e}/N$ ratio estimated with AgeNe for different combinations of age‐specific survival and fecundity. (A) On top, gradient of survivorship curves simulated, ranging from type III (blue, c<1) characterized by high juvenile mortality and low adult mortality; to type II (orange, c around 1), constant mortality and type I (red), low juvenile mortality and high adult mortality. At the bottom, simulated fecundity either increases or decreases exponentially with age following $F_{\text{Age}}={\text{exp}}^{f\times \text{Age}}$, with f ranging from −1 to 1. Sixteen simulated life tables were constructed with the same values of age at maturity and lifespan as the 16 studied species, and all possible survivorship curves and fecundity‐age relationships shown in Panels A and B). Slope and $R^{2}$ of the regression between adult lifespan and $N_{e}/N$ ratio for the 16 simulated species as a function of c, for constant fecundity with age (thin line) and exponential increase of fecundity with age with f=0.142 (thick line). (C) Slope and (D) $R^{2}$ of the regression between adult lifespan and $N_{e}/N$ ratio for the 16 simulated species for a gradient of values of c and f. In (C), warmer colors indicate steeper negative slopes; in (D) higher $R^{2}$.

Second, age‐specific fecundity was simulated with two models: constant and exponential. In the first model, fecundity is constant for all ages since maturity. In the second model, fecundity increases or decreases exponentially with age following $F_{\text{Age}}={\text{exp}}^{f\times \text{Age}}$, as it is often observed in marine fishes (Curtis and Vincent 2006). We first set f=0.142 as the median of the f values for the 16 species. Second, we took values of f ranging from −1 to 1 (Fig. 4A). We scaled maximum fecundity to 1 for all simulations.

For each combination of c and f, and for each fecundity model, we simulated all species life tables given age at maturity and lifespan. Then, we ran AgeNe and estimated $N_{e}/N$ for each simulated species and estimated the slope of the regression between adult lifespan and $N_{e}/N$ across all 16 species. We explored the impact of alternative fecundity–age models on the relationship between adult lifespan and $N_{e}/N$ (see details in Supporting Information).

### INTRASPECIFIC VARIATION IN GENETIC DIVERSITY

We addressed the potential effects of population structure, demography, and historical contingencies on genetic diversity by examining the extent of spatial variation in genetic diversity between the four populations within each species. First, we evaluated the relative amount of intraspecific compared to interspecific variation in genetic diversity. Then, we applied a z‐transformation of individual genetic diversity within each species to put spatial differences in within‐species diversity on the same scale. To detect similar spatial patterns of genetic diversity among species, we finally performed a hierarchical clustering analysis of the matrix of z‐transformed genetic diversity values with the pheatmap function available in pheatmap v1.0.12 R package.

### STATISTICAL ANALYSES

All statistical analyses were carried out using R‐3.6.1 (R Core Team, 2018). We fitted beta regression models between genetic diversity and any covariate with the R‐package betareg version 3.1‐3 (Cribari‐Neto and Zeileis 2010). We tested statistical interactions between any quantitative and qualitative covariates using likelihood tests with the lmtest version 0.9‐37 package (Zeileis and Hothorn 2002).

## Results

### WHOLE‐GENOME RESEQUENCING DATASET

We resequenced 300 individual genomes from 16 marine teleostean species, with high read quality scores (mean Q30 rate = 92.4%) and moderate duplication rates (10.8%) (Fig. S2). GC content was moderately variable among species and highly consistent among individuals of the same species, except for three individuals that showed a marked discrepancy with the overall GC content of their species (Fig. S2). These three individuals were thus removed from downstream analyses to avoid potential issues due to contamination or poor sequencing quality.

### ESTIMATION OF GENETIC DIVERSITY WITH GENOMESCOPE

The GenomeScope model successfully converged for all of the 297 individual genomes retained (Fig. S6E). The average depth of sequencing coverage per diploid genome exceeded 20× in most individuals. Estimated genome sizes were very consistent within species (Fig. S6A–C). Estimated levels of genetic diversity were also homogeneous among individuals of the same species with some few exceptions (e.g. *S. cinereus* and *S. typhle*) and most of the variability in genetic diversity was observed between species (Fig.  1D). Two individuals (one *D. puntazzo* and one *P. erythrinus*) showed a surprisingly high genetic diversity (more than twice the average level of their species), indicating possible issues in the estimation of genome‐wide heterozygosity. Therefore we removed these individuals from subsequent analysis, although their estimated genome size and GC content matched their average species values (therefore excluding contamination as a cause of genetic diversity estimation failures).

Observed values of genetic diversity ranged from 0.225% for *Lophius budegassa* to 1.415% for *Sardina pilchardus*. We found no correlation between species genetic diversity and genome size (p−value=0.983). The estimation of genetic diversity was robust to the choice for k‐mer lengths ranging from 21 to 25, suggesting a low sensitivity of GenomeScope regarding this parameter (Fig. S4). The fraction of reads mapped against reference genomes ranged between 96.72 and 98.50% for *D. labrax* and between 87.45 and 96.42 % for *S. pilchardus* (Table S2, Fig. S3). We found similar species genetic diversity estimates between GenomeScope and the GATK reference‐based variant calling approach for the two control species, representing extreme values within the range of genetic diversity in our dataset (Fig. 1B).

### ADULT LIFESPAN IS THE BEST PREDICTOR OF GENETIC DIVERSITY

We evaluated the effect of several key life history traits that potentially affect species genetic diversity (Table S1).

Two widely used predictors of population size, body size and trophic level, were not significantly correlated to genetic diversity (p−value=0.119 and 0.676, respectively, Fig. S8A and B). Although we detected a significant negative relationship between the logarithm of fecundity and propagule size (p−value=0.00131, slope = −0.4385±0.1076) as in Romiguier et al. (2014), we found no significant correlation between either propagule size (p−value=0.561), or the logarithm of fecundity (p−value=0.785) and genetic diversity (Fig. S8C and D).

By contrast, both lifespan (p−value=0.011) and adult lifespan (p−value=0.007) were significantly negatively correlated with genetic diversity (Table S1, Fig. 2). The percentage of variance explained by each variable reached 43.8% and 42.9 %, respectively. Repeating the same statistical analyses with genetic diversity estimates obtained either only from Mediterranean or Atlantic individuals led to the same results, revealing no effect of within‐species population structure on the relationship between genetic diversity and life history traits (Figs. 1C and S9, Table S3).

We found no significant interaction between hermaphroditism and any of the previous variables on genetic diversity. By contrast, parental care showed a significant interaction with lifespan (p−value=0.0011), adult lifespan (p−value=0.0008), and body size (p−value=0.0035) on genetic diversity. Brooding species (nest protection by males for *C. galerita*, *S. cinereus*, and *S. cantharus* and male abdominal brood‐pouch for *H. guttulatus* and *S. typhle*) had systematically lower genetic diversity than nonbrooding species with similar adult lifespan.

When considering only nonbrooding species, we found steeper negative correlations and higher percentages of between‐species variance in genetic diversity explained by lifespan ($p-\mathrm{value}=1.017\times 10^{-7}$, pseudo‐$R^{2}=0.851$) and adult lifespan ($p-\mathrm{value}=1.645\times 10^{-7}$, pseudo‐$R^{2}=0.829$, Fig. 2, Table S1). To test the relevance of considering this sub‐dataset, we estimated the slope of the regression and the pseudo‐$R^{2}$ for all combinations of 11 out of 16 species and compared the distribution of these values to the estimated slope and pseudo‐$R^{2}$ obtained for the 11 nonbrooding species (Fig. S13). The estimated slope for nonbrooders lied outside of the 95% confidence interval of the distribution of estimated slopes (slope=−0.129,95%CI =[−0.122,−0.049]) and the same was found for pseudo‐$R^{2}$ (pseudo‐$R^{2}=0.829,95\%$CI=[0.073,0.727]). Furthermore, considering nonbrooding species only, there was still no significant correlation between genetic diversity and trophic level (p−value=0.259), propagule size (p−value=0.170), and fecundity (p−value=0.390), but genetic diversity appeared significantly negatively correlated to body size ($p-\mathrm{value}=6.602\times 10^{-5}$, pseudo‐$R^{2}=0.616$). We did not detect any significant correlation between any trait variable and genetic diversity within the sub‐dataset of brooding species. However, this should be taken with caution given the very low number of brooding species (n=5) in our dataset.

Body size and lifespan were highly positively correlated traits in our dataset ($p-\mathrm{value}=0.0013,R^{2}=0.536$, Fig. S7). Thus, using empirical observations only, it was not possible to fully disentangle the impact of each of these traits among the possible determinants of genetic diversity in marine fishes. However, we found important differences in effect sizes for body size (slope=−0.014), lifespan (−0.095), and adult lifespan (−0.129), which rule out body size as a major determinant of diversity in our dataset.

### VARIANCE IN REPRODUCTIVE SUCCESS EXPLAINS LEVELS OF OBSERVED GENETIC DIVERSITY

To understand the mechanisms by which adult lifespan affects genetic diversity and test if it can alone explain our results, we built life tables for each of the 16 species by gradually incorporating age‐specific fecundity and survival, age at first maturity, lifespan, and sex‐specific differences in these parameters.

Nongenetic estimates of $N_{e}/N$ ratio obtained with AgeNe ranged from 0.104 in *L. budegassa* to 0.671 for *S. cinereus*. When considering the 16 species together, the $N_{e}/N$ ratio was not significantly correlated with genetic diversity (p-value=0.0935). However, four out of five brooding species had low genetic diversity despite high $N_{e}/N$ ratios (Fig. 3A). As previously observed, removing the five brooders increased the slope and the percentage of variance in genetic diversity explained by the $N_{e}/N$ ratio above null expectations obtained by removing groups of five species at random (slope=1.849,95%CI =[0.048,1.582], pseudo‐$R^{2}=0.55,95\%$CI =[0.004,0.533], Fig. S14). Thus, the $N_{e}/N$ ratio predicted by life tables was positively correlated to genetic diversity when considering nonbrooding species only (Fig. 3A, p-value=0.000966).

Our next step was to determine the impact of each component of life tables as well as their combinations on genetic diversity (Fig. 3C–G). Starting from a null model (Fig. 3C), in which species life tables differed only in lifespan, we found that the $N_{e}/N$ ratio ranged from 0.558 to 0.733, a variance much lower than that of observed genetic diversities. Then, adding separately age at maturity (Fig. 3D) or age‐specific survival (Fig. 3E) did not better predict the range of observed genetic diversities. However, combining age at maturity and age‐specific survival (Fig. 3F) or adding only age‐specific fecundity (Fig. 3G) enabled us to explain the range of observed diversity values. Finally, combining these three parameters together (age at maturity, age‐specific survival, and fecundity, model 8, Fig. S10H) resulted in the best fitted slope for both nonbrooding species and the whole dataset. Adding sex‐specific differences in life tables did not improve the fit, however (models 9–16, Fig. S10I–P).

Our final step was to further explore the role of the variance in reproductive success on genetic diversity by simulating genetic diversity at mutation‐drift equilibrium with the age‐specific vital rates of the 16 species.

We simulated a population of 2000 individuals with age‐specific survival and fecundity. As expected, including age‐specific vital rates decreased the equilibrium level of genetic diversity compared to expectations under the classical Wright‐Fisher model ($\theta =4N_{e}\mu =0.08\%$). It was reduced to 0.070% in the species with the least effect of age‐specific vital rates (*C. galerita*), and down to 0.010% in the species with the greatest effect (*L. budegassa*). Again, simulated genetic diversity was not correlated to genetic diversity considering all 16 species (p−value=0.297, Fig. 3B), but significantly positively correlated within the subsample of the 11 nonbrooding species (p−value=0.0115).

### LIFE TABLES DRIVE THE CORRELATION BETWEEN LIFESPAN AND THE $N_{e}/N$ RATIO

To determine the general effect of life table properties on the relation between adult lifespan and $N_{e}/N$ beyond the case of marine fish, we modeled 16 life tables with age at maturity and lifespan similar to those observed in our species but with simulated age‐specific survival and fecundity (Fig. 4A).

Considering models including constant fecundity with age, we found a significant relationship between adult lifespan and $N_{e}/N$ for species with type III survivorship curves (c<1) but not for species having an age‐specific survivorship curve constant, c, superior to 2, including type I species (Fig. 4B). The slope between adult lifespan and $N_{e}/N$ was steepest for type III species, reaching −0.053 for c=0.1. For c<2, the percentage of variation in $N_{e}/N$ explained by adult lifespan was higher than 60%. Interestingly, it reached a maximum for c=1.03 at 89% and abruptly dropped down around c=2 (Fig. 4B).

Then, we added an exponential increase in fecundity with age, first taking f=0.142, which is close to the mean empirical estimation across our 16 species (Fig. 4B). The slope between adult lifespan and $N_{e}/N$ became steeper for type I and type II species and reached −0.074 for extreme type III species (c=0.01). When we included this exponential increase of fecundity with age, the percentage of variation explained was superior for approximately all values of c, and the abrupt drop of the percentage of variation explained shifted toward higher c values, around c=3. Interestingly, we found significant positive relationships associated with low slope values when c became superior to 10 (type I species).

Then, we compared values of slope and $R^{2}$ for all c values and for f ranging from −1 to 1 (Fig. 4C and D). The steepest slope between adult lifespan and $N_{e}/N$ that we obtained reached −0.076 for extreme type III species (c around 0.1), and exponential constant, f, between 0.18 and 0.31. For type III and type II species (c<1), both the slope and the percentage of variation explained first increased with increasing exponential constant and then decreased. Significant negative relationships were found for c<1 for any values of f, except some extreme values near −1, whereas no significant relationship was found for c>1 when f is negative except for values of c near 1 and values of f near 0. The steepest slope and the highest percentage of variation explained were obtained for type III species with intermediate values of f (0.1<f<0.5) and for type II species (1<c<5) for positive values of f. For type I species, as c values increased, higher values of f are needed to obtain a significant negative relationship between adult lifespan and the $N_{e}/N$ ratio. Above c>20, no significant negative relationship was found for any values of f. Again, we found significant positive relationships and low slopes for c>15 and intermediate positive values of f.

We found similar results considering a power‐law relationship between age and fecundity, with slightly flatter slopes between $N_{e}/N$ and adult lifespan, and no significant correlations for extreme positive values of f and extreme low values of c (Fig. S16C). In contrast, we found limited or no impact of f on the relationship between $N_{e}/N$ and adult lifespan, respectively, for the linear and the polynomial age‐fecundity models (Fig. S16A and B).

## Discussion

In this study, we used whole‐genome high‐coverage sequencing data to estimate the genetic diversity of 16 marine teleost fish with similar geographic distribution ranges. We found that adult lifespan was the best predictor of genetic diversity, species with long reproductive lifespans generally having lower genetic diversities (Fig. 2). Longevity was already identified as one of the most important determinants of genetic diversity across metazoans and plants, in which it also correlates with the efficacy of purifying selection (Romiguier et al. 2014; Chen et al. 2017). A positive correlation between longevity and the ratio of nonsynonymous to synonymous substitutions (dN/dS) was also found in teleost fishes (Rolland et al. 2020), thus suggesting lower $N_{e}$ in long‐lived species. However, the mechanisms by which lifespan impacts genetic diversity remain poorly understood and may differ among taxonomic groups. Here we showed that age‐specific fecundity and survival (i.e., vital rates), summarized in life tables, naturally predict the empirical correlation between adult lifespan and genetic diversity in marine fishes.

### IMPACT OF LIFE TABLES ON GENETIC DIVERSITY

On a broad taxonomic scale including plants and animals, Waples et al. (2013) showed that almost half of the variance in $N_{e}/N$ estimated from life tables can be explained with only two life history traits: age at maturity and adult lifespan. Therefore, the effect of adult lifespan on genetic diversity should reflect variations in age‐specific fecundity and survival across species. If the species vital rates used to derive $N_{e}/N$ ratios are relatively stable over time, the reduction in $N_{e}$ due to lifetime variance in reproductive success should not only apply to contemporary time scales but more generally throughout the coalescent time. Thus, a direct impact of life tables on genetic diversity can be expected for iteroparous species with overlapping generations.

Using both an analytical (with AgeNe) and a simulation‐based (with SLiM) approach, we showed that age‐specific survival and fecundity rates alone can explain a significant fraction of the variance in genetic diversity among species (Fig. 3A and B). This may appear surprising at first sight, considering that we did not account for variation in census population size among species, which vary by several orders of magnitude in marine fishes (Hauser and Carvalho 2008). Our results thus support that intrinsic vital rates are crucial demographic components of the neutral model to understand differences in levels of genetic diversity in marine fishes. But how generalizable is this finding to other taxa?

Age‐specific survivorship curves are one of the main biological components of life tables. Three main types of survivorship curves are classically distinguished: type I curves are characterized by low juvenile and adult mortality combined with an abrupt decrease of survival when approaching the maximum age (e.g., mammals); in type II curves, survival is relatively constant during lifetime (e.g., birds) while type III curves are characterized by high juvenile mortality followed by low adult mortality (e.g., fishes and marine invertebrates). Type III survivorship curves favor the disproportionate contribution of a few lucky winners that survive to old age, compared to type I survivorship curves, where individuals have more equal contributions to reproduction, generating a lower variance in reproductive success. Thus, in type III species, higher lifetime variance in reproductive success is expected as the lifespan of a species increases. By simulating extreme type III survivorship curves (c=0.1) for our 16 species while keeping their true adult lifespans, we found that $N_{e}/N$ can decrease by at most 0.05 per year of lifespan (Fig. 4B, extreme left). This can theoretically induce up to a 60% difference in genetic diversity between the species with the shortest and the longest lifespans of our dataset. In contrast, we found no correlation between adult lifespan and $N_{e}/N$ when simulating type I survivorship curves with the true lifespan values of the 16 species studied here (Fig. 4B, c>2), meaning than lifespan and variance in reproductive success may have limited influence in other taxonomic groups, such as birds or mammals.

Another important component of life tables is age‐specific fecundity. In marine fishes, fecundity is positively correlated to female ovary size, and the relationship between fecundity and age is usually well approximated with an exponential ($F=a{\text{exp}}^{Ab}$) or power‐law ($F=aA^{b}$) function. By adding an exponential increase in fecundity with age to our simulations, we found that $N_{e}/N$ decreases even more strongly with increasing adult lifespan ($N_{e}/N$ decreases by up to 0.07 per extra year of reproductive life). Using both type III survivorship and exponentially increasing fecundity with age, we could thus predict up to 84% of the variance in genetic diversity between species with the shortest and longest lifespans.

We found that $N_{e}/N$ predicted from fecundity alone or age at maturity combined with age‐specific survival, explained as much variation in genetic diversity as life tables with both these components (Fig. 3). This is because both these scenarios create sharp differences in fitness between young and old age classes. By contrast, variation in age at maturity alone (all other parameters being held constant across species) introduces some variation in $N_{e}/N$ because the onset of reproduction age varies from 1 to 7 years depending on the species, but this effect is buffered by the long subsequent period during which adults will reproduce equally. Similarly, the effect of survival alone is insufficient if individuals of all species start reproducing early enough at the age of 1 year.

Although these predicted relationships were pretty close to our empirical findings, genome‐wide heterozygosity decreased by about 0.09 per additional year of lifespan in our real dataset (Fig. 2), which seems to be a stronger effect compared to theoretical predictions based on vital rates alone. It is thus likely that other correlates of adult lifespan and unaccounted factors also contribute to observed differences in genetic diversity among species.

### CORRELATED EFFECTS

When relating measures of diversity with the estimates of $N_{e}/N$ derived from life tables, we did not take into account differences in census size (N) between species. Population census sizes can be huge and are notoriously difficult to estimate in marine fishes. For that reason, abundance data remain largely unavailable for the 16 species of this study. We nevertheless expect long‐lived species to have lower abundance compared to short‐lived species because in marine fishes N is generally negatively correlated to body size (White et al. 2007), which is itself positively correlated to adult lifespan in our dataset (Fig. S7). Hence, while we have demonstrated here that variation in vital rates has a direct effect on long‐term genetic diversity, the slope between adult lifespan and genetic diversity may be inflated by uncontrolled variation in N. Recent genome‐wide comparative studies found negative correlations between $N_{e}/N$ and N in pinnipeds (Peart et al. 2020) as well as between genetic diversity and body size in butterflies and birds (Brüniche‐Olsen et al. 2019; Mackintosh et al. 2019). Here, a highly significant negative correlation was found between genetic diversity and body size and the strength of that correlation was comparable to that found in a meta‐analysis of microsatellite diversity using catch data and body size as proxies for fish abundance (Mccusker and Bentzen 2010). We note, however, that body size was not as good a predictor of genetic diversity as lifespan and adult lifespan for the 11 nonbrooding species and it was even not significant in the whole dataset of the 16 species (Table S1).

Another potentially confounding effect is the impact of r/K strategies, which are the main determinant of genetic diversity across metazoans (Romiguier et al. 2014). In our dataset, fecundity and propagule size (proxies for the r/K gradient) showed only little variance compared to their range of variation across metazoans, and none of them were correlated to adult lifespan. However, we found that the five brooding species of our dataset, which are typical *K*‐strategists, displayed lower genetic diversities with respect to their adult lifespan (Fig. 2). Most interestingly, when these species were removed from the analysis, the effect of adult lifespan on genetic diversity was amplified, indicating a potentially confounding effect of parental care in marine fishes. Alternatively, low levels of genetic diversity in brooding species can also be explained by underestimated lifetime variance in reproductive success by AgeNe due to unaccounted variance in reproductive success within age classes. This may be particularly important in males as the age‐fecundity relationship is empirically estimated for females only. This effect could be high for species with strong sexual selection and mate choice (Hastings 1988; Naud et al. 2009). Moreover, most of these species inhabit lagoons and coastal habitats, corresponding to smaller and more instable ecological niches compared to species with no parental care, thus potentially resulting in lower long‐term abundances. The discrepancy introduced by brooders in the relationship that we observed here between adult lifespan and genetic diversity may thus involve a variety of effects that remain to be elucidated.

Temporal fluctuations of effective population size may also have impacted observed levels of genetic diversity (Nei et al. 1975). All studied species possibly went through a bottleneck during the Last Glacial Maximum (Jenkins et al. 2018), which may have simultaneously decreased their genetic diversities. As the time of return to mutation‐drift equilibrium is positively correlated to generation time, which is itself directly linked to adult lifespan, we may expect long‐lived species to have recovered less genetic variation than short‐lived species following their latest bottleneck. Moreover, long‐lived species may not have recovered their pre‐bottleneck population sizes as rapidly as short‐lived species. If true, the negative relationship between adult lifespan and genetic diversity may be inflated compared to the sole effect of life tables.

Variation in mutation rates between species could not be accounted for due to a lack of estimates. However, if species‐specific mutation rates were correlated with adult lifespan, we would expect mutation rate variation to have a direct effect on genetic diversity. Mutation rate could be linked with species life history traits through three possible mechanisms. First, the drift‐barrier hypothesis predicts a negative correlation between species effective population size and the per‐generation mutation rate (Sung et al. 2012). However, this hypothesis cannot explain our results because species with the highest effective population sizes have the highest genetic diversity. Second, species with larger genome size tend to have more germline cell divisions, hence possibly higher mutation rates. But we did not find any correlation between genome size and genetic diversity or any other qualitative and quantitative life history traits. Third, species with longer generation time, which is positively correlated to lifespan and age at maturity, may have higher per‐generation mutation rate as older individuals accumulate more germinal mutations throughout their lives. Again, under this assumption, we would expect species with longer lifespan to have higher mutation rate and genetic diversity, which goes against our observations. In summary, variation in mutation rates among species due to differences in lifespan is unlikely to explain the negative lifespan‐diversity relationship we observed. If anything, variation in mutation rates should theoretically oppose this relationship.

Using one of the few direct estimates of the per‐generation mutation rate in fish, Feng et al. (2017) explained the surprisingly low nucleotide diversity found in the Atlantic herring *Clupea harengus* (π=0.3%) by a very low mutation rate of $2\times 10^{9}$ per base per generation estimated from pedigree analysis. Although the herring is one of the most abundant and fecund pelagic species in the North Atlantic Ocean, its genetic diversity appears approximately 80% lower than that of the European pilchard *S. pilchardus*, another member of the *Clupeidae* family that shows the highest diversity in our study. Even if *C. harengus* has a larger body size (approximately 30 cm, compared to 20 cm for *S. pilchardus*; Froese et al. 2000), it has above all a much longer lifespan (between 12 and 25 years) and a later age at maturity (between 2 and 6.5 years) (Jennings and Beverton 1991). Considering even the lowest estimate of adult lifespan reported for the herring (10 years), the corresponding genetic diversity predicted by our model linking adult lifespan to genetic diversity would be around 0.5%, which is pretty close to the empirical estimate.

Finally, we did not take into account the erosion of neutral diversity through linked selection. Addressing that issue would need to generate local estimates of nucleotide diversity and population recombination rate along the genome of each species using resequencing data aligned to a reference assembly, which was out of the scope of this study. The predicted effect of linked selection could be, however, to remove more diversity in species with large compared to small $N_{e}$. It is therefore likely that linked selection would rather attenuate the negative relationship between adult lifespan and genetic diversity compared to neutral predictions.

### CONCLUSION

Here we used a simple approach to generate reference‐free genome‐wide estimates of diversity with k‐mer analysis. Tested on two species with genetic diversities ranging from 0.22% to 1.42% the k‐mer approach performed close to the level of a high‐standard reference‐based method in capturing fine‐scale variation in diversity between evolutionary lineages and even populations of the same species. This opens the possibility to address the determinants of genetic diversity in other groups of taxa at limited costs without relying on existing genomics resources. Across metazoans, the level of genetic diversity showed no significant relationship with the species’ conservation status (Romiguier et al. 2014). Studies performed at lower phylogenetical scales such as in Darwin's finches and pinnipeds, however, found reduced contemporary genetic diversity in threatened compared to nonthreatened species (Brüniche‐Olsen et al. 2019; Peart et al. 2020). Our results complement and extend this literature by showing the importance of taking into account life tables in comparisons of genetic diversity between species.

### AUTHOR CONTRIBUTIONS

P.B., T.B., and P.‐A.G. wrote the manuscript. P.B. and P.‐A.G performed fieldwork. P.B. performed molecular experiments, and all bioinformatics and evolutionary genomics analyses with inputs from T.B. and P.‐A.G. P.‐A.G. conceived the project and managed financial support and genome sequencing.

## DATA ARCHIVING

Data and scripts used in this study are freely available in the GitHub repository https://github.com/pierrebarry/life_tables_genetic_diversity_marine_fishes. All sampling metadata are accessible under GEOME at the CoGeDiv Project Homepage: https://geome‐db.org/workbench/project‐overview?projectId=357. Sequence reads have been deposited in the GenBank Sequence Read Archive under the accession code BioProject ID PRJNA777424.

## Supporting information


**Figure S1**: *k‐mer* frequency‐coverage relationship and estimation by GenomeScope v.1.0 (Vurture et al., 2017) for two species, *L. budegassa* and *M. surmuletus*.
**Table S1**: Statistical relationships between species genetic diversity and life history traits.
**Table S2**: Mapping and variant calling statistics for *D. labrax* and *S. pilchardus* individuals.
**Table S3**: Statistical relationships between different estimations of species genetic diversity and life history traits.
**Figure S2**: Number of reads ($10^{9}$ bp), percentage of reads with quality superior to Q30, duplication rate and GC content after filtering, correcting and trimming steps carried out with fastp v.0.20.0 (Chen et al. 2018).
**Figure S3**: Mapping statistics.
**Figure S4**: Effect of k−mer length on genetic diversity estimation with GenomeScope v.1.0 (Vurture et al. 2017).
**Figure S5**: Individual whole‐genome sequences features estimated with GenomeScope v.1.0 (Vurture et al. 2017).
**Figure S6**: Relationship between individual mean genome‐wide heterozygosity estimated with the k‐mer based reference‐free approach in GenomeScope (x‐axis), and the high standard reference‐based approach in GATK (y‐axis), for european sea bass (*D. labrax*, dots) and european pilchard (*S. pilchardus*, triangle).
**Figure S7**: Correlation matrix between genetic diversity and all quantitative life history traits.
**Figure S8**: Relationship between species median genetic diversity (%) and 5 covariables.
**Figure S9**: Effect of population structure on genetic diversity estimates.
**Figure S10**: Relationship between relative species genetic diversity and relative $N_{e}/N$ estimated by AgeNe for 16 sets of life tables.
**Figure S11**: Relationship between relative species genetic diversity and simulated genetic diversity with forward‐in‐time simulations for 16 sets of life tables.
**Figure S12**: Residuals of the linear model between genetic diversity and variance in reproductive success estimated from various combinations of life tables from a model with slope equals 1 and intercept 0.
**Figure S13**: Distribution of slope and pseudo *R* ^2^ of the beta regression between adult lifespan and genetic diversity for random subsets of 11 species.
**Figure S14**: Distribution of slope and pseudo $R^{2}$ of the beta regression between $N_{e}/N$ and genetic diversity for random subsets of 11 species.
**Figure S15**: Distribution of slope and pseudo $R^{2}$ of the beta regression between simulated, with SLiM v.3.3.1, and observed genetic diversity for random subsets of 11 species.
**Figure S16**: Slope of and proportion of variance explained by linear models between adult lifespan and $N_{e}/N$ estimated with AgeNe for different combinations of age‐specific survival and fecundity for three fecundity‐age models: linear, polynomial and power‐law.
**Figure S17**: Population size count for the 50 iterations of the 16 species for set 1 of life tables (age at first maturity at 1 year old, constant age‐specific survival rate, constant age‐specific fecundity and no differences between sex‐specific life tables).
**Figure S18**: Population size count for the 50 iterations of the 16 species for set 2 of life tables (age at first maturity at 1 year old, increasing age‐specific survival rate, constant age‐specific fecundity and no differences between sex‐specific life tables).
**Figure S19**: Population size count for the 50 iterations of the 16 species for set 3 of life tables (age at first maturity at 1 year old, constant age‐specific survival rate, increasing age‐specific fecundity and no differences between sex‐specific life tables).
**Figure S20**: Population size count for the 50 iterations of the 16 species for set 4 of life tables (age at first maturity at 1 year old, increasing age‐specific survival rate, increasing age‐specific fecundity and no differences between sex‐specific life tables).
**Figure S21**: Population size count for the 50 iterations of the 16 species for set 5 of life tables (age at first maturity at 1 year old, constant age‐specific survival rate, constant age‐specific fecundity and sex‐specific differences in life tables).
**Figure S22**: Population size count for the 50 iterations of the 16 species for set 6 of life tables (age at first maturity at 1 year old, increasing age‐specific survival rate, constant age‐specific fecundity and sex‐specific differences in life tables).
**Figure S23**: Population size count for the 50 iterations of the 16 species for set 7 of life tables (age at first maturity at 1 year old, constant age‐specific survival rate, increasing age‐specific fecundity and sex‐specific differences in life tables).
**Figure S24**: Population size count for the 50 iterations of the 16 species for set 8 of life tables (age at first maturity at 1 year old, increasing age‐specific survival rate, increasing age‐specific fecundity and sex‐specific differences in life tables).
**Figure S25**: Genetic diversity simulated for each of the 16 species for set 1 of life tables (age at first maturity at 1 year old, constant age‐specific survival rate, constant age‐specific fecundity and no differences between sex‐specific life tables).
**Figure S26**: Genetic diversity simulated for each of the 16 species for set 2 of life tables (age at first maturity at 1 year old, increasing age‐specific survival rate, constant age‐specific fecundity and no differences between sex‐specific life tables).
**Figure S27**: Genetic diversity simulated for each of the 16 species for set 3 of life tables (age at first maturity at 1 year old, constant age‐specific survival rate, increasing age‐specific fecundity and no differences between sex‐specific life tables).
**Figure S28**: Genetic diversity simulated for each of the 16 species for set 4 of life tables (age at first maturity at 1 year old, increasing age‐specific survival rate, increasing age‐specific fecundity and no differences between sex‐specific life tables).
**Figure S29**: Genetic diversity simulated for each of the 16 species for set 5 of life tables (age at first maturity at 1 year old, constant age‐specific survival rate, constant age‐specific fecundity and sex‐specific differences in life tables).
**Figure S30**: Genetic diversity simulated for each of the 16 species for set 6 of life tables (age at first maturity at 1 year old, increasing age‐specific survival rate, constant age‐specific fecundity and sex‐specific differences in life tables).
**Figure S31**: Genetic diversity simulated for each of the 16 species for set 7 of life tables (age at first maturity at 1 year old, constant age‐specific survival rate, increasing age‐specific fecundity and sex‐specific differences in life tables).
**Figure S32**: Genetic diversity simulated for each of the 16 species for set 8 of life tables (age at first maturity at 1 year old, increasing age‐specific survival rate, increasing age‐specific fecundity and sex‐specific differences in life tables).

Data S1

Data S2
