# Supplementary material for: Age‐specific survivorship and fecundity shape genetic diversity in marine fishes
Source: Evol Lett. 2021 Dec 24;6(1):46–62. doi: 10.1002/evl3.265 (PMC8802244; doi:10.1002/evl3.265)
Supplement: Supplementary file 1 — Figure S1: k‐mer frequency‐coverage relationship and estimation by GenomeScope v.1.0 (Vurture et al., 2017) for two species, L. budegassa and M. surmuletus. Table S1: Statistical relationships between species genetic diversity and life history traits. Table S2: Mapping and variant calling statistics for D. labrax and S. pilchardus individuals. Table S3: Statistical relationships between different estimations of species genetic diversity and life history traits. Figure S2: Number of reads (109 bp), percentage of reads with quality superior to Q30, duplication rate and GC content after filtering, correcting and trimming steps carried out with fastp v.0.20.0 (Chen et al. 2018). Figure S3: Mapping statistics. Figure S4: Effect of k−mer length on genetic diversity estimation with GenomeScope v.1.0 (Vurture et al. 2017). Figure S5: Individual whole‐genome sequences features estimated with GenomeScope v.1.0 (Vurture et al. 2017). Figure S6: Relationship between individual mean genome‐wide heterozygosity estimated with the k‐mer based reference‐free approach in GenomeScope (x‐axis), and the high standard reference‐based approach in GATK (y‐axis), for european sea bass (D. labrax, dots) and european pilchard (S. pilchardus, triangle). Figure S7: Correlation matrix between genetic diversity and all quantitative life history traits. Figure S8: Relationship between species median genetic diversity (%) and 5 covariables. Figure S9: Effect of population structure on genetic diversity estimates. Figure S10: Relationship between relative species genetic diversity and relative Ne/N estimated by AgeNe for 16 sets of life tables. Figure S11: Relationship between relative species genetic diversity and simulated genetic diversity with forward‐in‐time simulations for 16 sets of life tables. Figure S12: Residuals of the linear model between genetic diversity and variance in reproductive success estimated from various combinations of life tables from a model with slope equals 1 and interce [file EVL3-6-46-s003.pdf]

Supplementary Material to Barry and al. ”Age-specific survivorship and fecundity shape genetic diversity in marine fishes”

Contents

Additional methods **2**
Sampling and sequencing 2
Collection of life history traits database 2
Estimation of genetic diversity with GenomeScope 2
Forward simulations 4
Evaluating the impact of life tables beyond marine fish 4

Additional results **4**
Whole-genome resequencing data set 4
Intraspecific variation in genetic diversity 5

Additional tables **6**

Additional figures **7**

Additional methods

Sampling, DNA extraction, whole-genome sequencing and reads quality control

We sampled 16 marine teleostean fish species presenting a wide diversity of life history strategies expected to affect genetic diversity. For 12 of these species, 20 individuals were sampled (5 per location). For the 4 other species, the total number of samples ranged from 10 to 19 (Table 1). Individuals were either sampled from landings in local fish markets, captured in the field (using hand nets, lure fishing, spearfishing or beach seines) or provided by collaborators. The majority of the sampling was done in 2018 and 2019. Whole-genomic DNA was extracted from fin or tissue clips stored in 95% ethanol using the NucleoSpin Tissue Kit (Macherey-Nagel) and treated with RNAse A to remove residual RNA. Double-stranded nucleic acid concentration was quantified using Qubit2.0 and standardized to 20ng per μl. Individual whole-genome sequencing libraries were prepared following the Illumina TruSeq DNA PCR-Free Protocol and sequenced by Genewiz Inc (USA). Libraries were quantified and multiplexed by groups of 40 individuals and sequenced on two S4 flow cells on a NovaSeq6000 instrument (Illumina) to generate 150 bp paired-end reads, targetting an average read depth of 20X per individual. Raw reads were preprocessed with `fastp` v.0.20.0 (Chen et al., 2018) using default parameters, allowing quality control, filtering by quality, length and complexity, and adapter trimming to be performed in a single step. Base correction was performed using a quality comparison between overlapping bases of paired-end reads, and polyG tail trimming was enabled to correct for artefactual G repetitions occurring in Novaseq read tails.

Collection of life history traits database

As growth is indeterminate in fish, we defined adult body size as the infinite length, L_{inf} determined by the Von Bertalanffy equation ($L_t = L_{inf}[1 - exp^{-K(t-t0)}]$), that links individual body size L_t to age t, with K a parameter defining the shape of this relationship (Pauly et al., 1987). We estimated adult body size as the median of all L_{inf} values reported for each species in the online database Fishbase (Froese et al., 2000). As L_{inf} was not documented in Fishbase for *D. puntazzo* and *C. galerita*, we took the median of the values reported in (Kraljević et al., 2007) and (Domínguez-Seoane et al., 2006) for *D. puntazzo*, and the maximum length observed in (Milton, 1983) for *C. galerita*. Trophic level was retrieved from Fishbase. Fecundity was defined as the absolute fecundity, i.e. the mean number of eggs in an ovary of a female in a single spawning event. Females may spawn several times during one reproductive season (Ganias et al., 2003; Murua and Motos, 2006), so absolute fecundity is not the value most directly relevant to global genetic diversity. However, it is the most commonly reported in the literature as the number of spawnings events per reproductive season is difficult to measure. Because fecundity is proportional to individual body size, we computed fecundity at infinite length, L_{inf}. Propagule size was determined following Romiguier et al. (2014), as the size of the dispersal stage that becomes independent of the parents. For all species of this study, this corresponded to egg diameter, except for brooders, for which we used hatching size. All propagule size data were retrieved from species-specific references. Age at maturity was defined as the age at which 50% of the population is mature. Values for age at maturity were taken from Tsikliras and Stergiou (2015) for seven species while other values were retrieved from species-specific references. Likewise, lifespan values were taken from (Tsikliras and Stergiou, 2015) for six species and completed with specific references. Finally, adult lifespan was defined as $Lifespan - Age\ at\ Maturity$ (Waples et al., 2013).

Estimation of genetic diversity with GenomeScope

Provided a sufficient average coverage depth (e.g. 20X), `GenomeScope` evaluates the fraction of heterozygous sites from the ratio of the height of the heterozygous to the homozygous k-mer peak, occurring at 50% (i.e. 10X) and 100% (i.e. 20X) of the average coverage depth, respectively. The number of different possible k-mers (and thus the precision of the method) increases with k, but so does the runtime and the probability of "wrong" k-mers due to sequencing errors. We set $k = 21$ as recommended by `GenomeScope` and performed a sensitivity analysis by estimating genetic diversity and genome size for one individual of *D. labrax* using k from 17 to 25 (Fig S4).

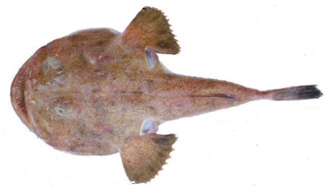

Lophius budegassa:
high genome size and
low diversity (0.225 %)

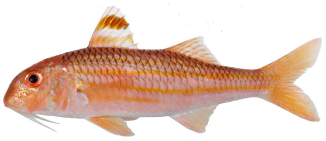

Mullus surmuletus:
low genome size and
high diversity (1.135 %)

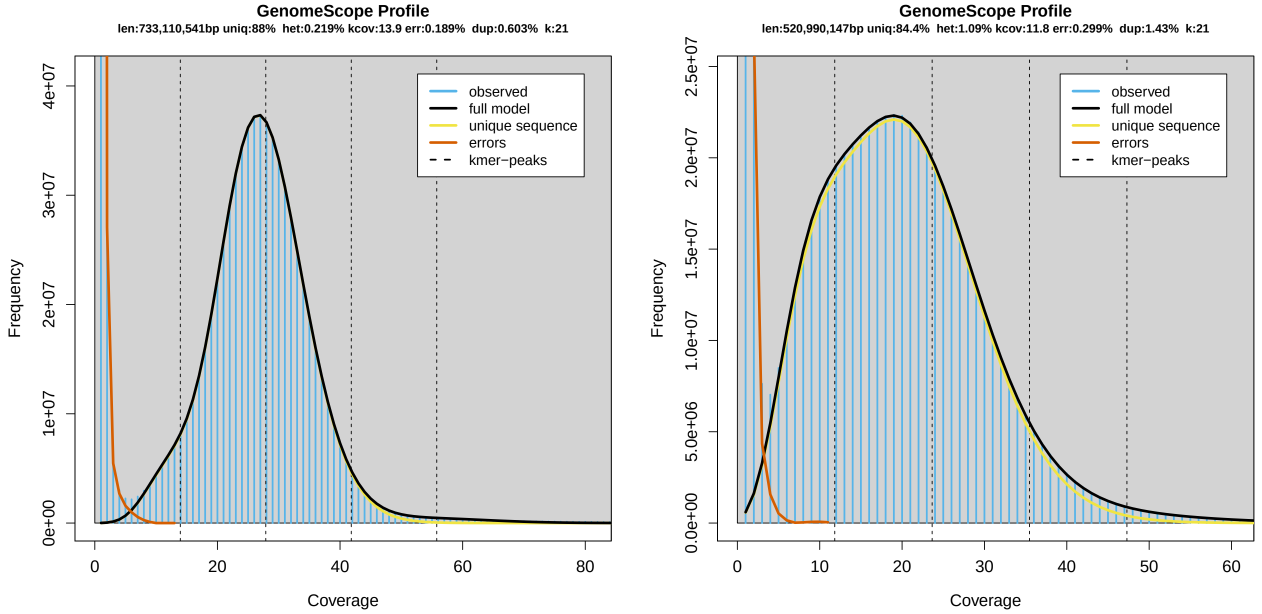

Figure S1: $k - mer$ **frequency-coverage relationship and estimation by `GenomeScope` v.1.0 (Vurture et al., 2017) for two species, *L. budegassa* and *M.surmuletus***

In order to assess the reliability of `GenomeScope`, the 20 resequenced genomes for two species (*S. pilchardus* and *D. labrax*) were aligned with `bwa-mem` v.0.7.17 (Li and Durbin, 2009) to the reference genomes retrieved from Louro et al. (2019) and Tine et al. (2014) for *S. pilchardus* and *D. labrax*, respectively. We then removed PCR duplicates with the Picard tools `MarkDuplicates` v.2.23.2. We followed the best-practice pipeline in `GATK` v.4.1.6.0 for variant calling (Poplin et al., 2018): we ran `HaplotypeCaller` with default options to generate individual GVCFs files, stored them in a database with `GenomicsDBImport` and finally computed VCF files with `GenotypeGVCFs`. We didn't apply post variant calling filtering steps, such as hard filters on genotype quality scores or Hardy-Weinberg Equilibrium criterion, in order to

avoid potential bias in the comparison of genetic diversity between species with very different rates of heterozygosity. However, we assume that possible bias due to the absence of variant filtering should not impact differences among individuals within each species. However, we generated VCF files with all sites, including non-variant ones, to avoid underestimation of genetic diversity due to the assumption that missing sites are homozygous for the reference allele. We estimated species genetic diversity as:

$$\pi = \frac{\sum^{L}\sum_{i<j} k_{ij}}{\sum^{L}\binom{n}{2}} \tag{S1}$$

where $\sum_{i<j} k_{ij}$ is the number of pairwise nucleotide differences between all haplotypes at a given site, $\binom{n}{2}$ is the total number of pairwise nucleotide comparison between all haplotypes at a given site, and L, the number of sites. We thus exclude missing data from the analysis. However, we include multiallelic sites as removing these sites may underestimate genetic diversity, especially for species with high genetic diversity.

Forward simulations

We used `SLiM` v.3.3.1 (Haller and Messer, 2017) to perform forward simulations to estimate genetic diversity at mutation-drift equilibrium incorporating age-specific survival and fecundity. For each individual, the number of offspring produced per year was determined by a Poisson distribution with mean $\lambda_{S,a}$ specific to each species and each age. Age at first maturity was set to 1 for all simulations. To keep population size constant in these non Wright-Fisher forwards simulations, we introduced a carrying capacity parameter, allowing population size to fluctuate around this capacity (Fig S17 - S24). We arbitrarily set this parameter to $N = 2000$, and simulated non-recombining 1Mb loci with a mutation rate of $\mu = 1e^{-7}$. Each simulation was run for 25000 years, which was long enough for genetic diversity to reach mutation-drift equilibrium (Fig S25 - S32). For each simulation, we estimated the mean genetic diversity (i.e., the proportion of heterozygous sites along the 1Mb locus) over the last 10000 years after checking that an equilibrium has been reached. For each species, we ran 50 replicates and defined the genetic diversity predicted by a given simulation scenario as the mean genetic diversity at equilibrium averaged over the 50 replicates.

Evaluating the impact of life tables beyond marine fish

We explored the impact of alternative fecundity-age models on the relationship between adult lifespan and $\frac{N_e}{N}$ using three additional biologically realistic models: linear ($F_{Age} = a \times Age + b$), polynomial ($F_{Age} = [Age - AgeMat][(AgeMat + Lifespan - Age)^2]$, common in mammal) (Gage, 2001) and power-law ($F_{Age} = Age^f$). For the linear and the polynomial model, f describes the maximum fecundity at lifespan and age with the highest fecundity, respectively (i.e. higher absolute values of f correspond to higher differences in fecundity between low and high fecund ages for both models). f was between -1 and 1 for the linear and the polynomial model. For the power-law model, we took values of f from -5 to 5.

Additional results

Whole-genome resequencing data set

We resequenced 300 individual genomes from 16 marine teleostean species, generating from 59.86×10^6 to 200.92×10^6 reads per individual (mean = 129×10^6, sd = 20×10^6, Fig S2). The

read quality score (Q30 rate) ranged between 88% and 94% (mean = 92.4%, sd = 1.1) and the duplication rate lied between 5 and 15% (mean = 10.8%, sd = 2.6) (Fig S2). GC content was moderately variable among species and highly consistent among individuals of the same species, except for one individual of *S. cabrilla*, *D. puntazzo* and *M. surmuletus* that showed a marked discrepancy with the overall GC content of their species (Fig S2). These three individuals were thus removed from downstream analyses to avoid potential issues due to contamination or poor sequencing quality (see discussion).

Intraspecific variation in genetic diversity

Although, we detected slight differences in within-species genetic diversity between individuals of different basins, this does not affect the results linking species' genetic diversity to life-history traits. However, regarding variation in within-species genetic diveristy, we distinguished two clusters: the first one included 9 species with generally lower genetic diversity in the Mediterranean than Atlantic localities, while the opposite was observed in the second cluster (7 species) (Fig 1C). The species of the second cluster are often found in coastal habitats, lagoons, estuaries whereas species of the first cluster are rather pelagic, epi-pelagic or benthic species. The only exception was the presence of *H. guttulatus* in the Atlantic cluster.

Additional tables

Dataset	Predictor	*p-value*	Pseudo R^2	Slope estimate (± 95% interval)
Whole data set	Body size	0.119	0.192	$-0.006(-0.014; 0.002)$
	Trophic level	0.676	0.012	$-0.091(-0.524; 0.343)$
	Propagule size	0.562	0.015	$-0.014(-0.062; 0.034)$
	Fecundity	0.653	0.013	$-1.22e^{-5}(-6.63e^{-5}; 4.20e^{-5})$
	Lifespan	**0.0107**	0.438	$-0.062(-0.111; -0.013)$
	Adult lifespan	**0.0070**	0.429	$-0.089(-0.156; -0.023)$
	Hermaphroditism	0.434	0.034	$0.1779(-0.278; 0.633)$
	Parental Care	0.274	0.075	$-0.273(-0.772; 0.226)$
No parental care	Body size	$\mathbf{6.60e^{-5}}$	0.616	$-0.014(-0.021; -0.007)$
	Trophic level	0.256	0.093	$-0.326(-0.902; 0.251)$
	Propagule size	0.170	0.175	$-0.518(-1.273; 0.237)$
	Fecundity	0.390	0.056	$-2.51e^{-5}(-8.35e^{-5}; 3.33e^{-5})$
	Lifespan	$\mathbf{1.017e^{-7}}$	0.851	$-0.095(-0.131; -0.060)$
	Adult lifespan	$\mathbf{1.65e^{-7}}$	0.829	$-0.129(-0.179; -0.080)$
	Hermaphroditism	0.454	0.044	$0.206(-0.345; 0.757)$

Table S1: **Statistical relationships between species genetic diversity and life history traits** - Genetic diversity was fitted to 6 quantitative (body size, trophic level, propagule size, fecundity, lifespan and adult lifespan) and two qualitative predictors (hermaphroditism and parental care) with a beta regression model using the `betareg` R package (Zeileis and Hothorn, 2002). In the upper part of the table, regressions were performed with the whole dataset, while in the lower part only the 11 non-brooding species were considered.

Table S2: **Mapping and variant calling statistics for *D. labrax* and *S. pilchardus* individuals** - For each, individual, number and percentage of reads mapped with `bwa-mem` v.0.7.17 (Li and Durbin, 2009). Individual GCVF files were created from bam files with `HaplotypeCaller` from `GATK` v.4.1.6.0 (Poplin et al., 2018), then stored in a GCVF database with `GenomicsDBImport`, and VCF files were finally generated with `GenotypeGVCFs`. Individual heterozygosity was estimated with a custom script. O_{het} is the number of observed heterozygous positions at N_{sites} number of variable sites. $Het_{bwa+GATK}$ and $Het_{GenomeScope}$ correspond to genome-wide average heterozygosity estimated with the variant calling and `GenomeScope` approaches respectively. Sample names indicate geographical origin (Li = Gulf of Lion, Mu = Costa Calida, Fa = Algarve, Ga = Bay of Biscay).

Species	Sample	Reads mapped	% reads mapped	O_{het}	N_{sites}	$Het_{bwa+GATK}$	$Het_{GenomeScope}$
D. labrax	DlabrFa1	113 750 879	98.50	2 147 840	503 641 870	0.4264618	0.4023185
	DlabrFa3	113 675 336	98.47	2 208 200	503 847 656	0.4382674	0.4056315
	DlabrFa4	119 760 663	98.26	2 168 714	503 981 403	0.4303163	0.3963415
	DlabrFa5	111 632 052	98.40	2 280 744	503 764 199	0.4527404	0.4370325
	DlabrFa6	118 251 538	98.39	2 153 188	503 902 634	0.4273024	0.391308
	DlabrMu1	111 356 113	98.20	1 719 388	502 790 222	0.3419693	0.2962895
	DlabrMu2	78 923 334	96.72	1 930 312	498 084 060	0.3875474	0.3733955
	DlabrMu3	100 325 589	98.00	1 874 416	502 625 989	0.3729246	0.332756
	DlabrMu4	113 701 410	98.29	1 955 294	503 912 767	0.3880223	0.355843
	DlabrMu6	117 131 984	98.11	1 923 386	503 422 444	0.3820620	0.3329545
	DlabrLi1	100 519 118	98.16	1 919 555	503 396 155	0.3813209	0.3627435
	DlabrLi2	103 717 839	98.18	1 926 045	503 259 727	0.3827139	0.3566875
	DlabrLi3	101 815 078	98.18	1 917 988	503 399 019	0.3810075	0.3713805
	DlabrLi4	94 399 356	98.21	1 920 186	502 848 166	0.3818620	0.3520245
	DlabrLi5	115 515 514	98.06	1 918 720	503 724 011	0.3809070	0.354731
	DlabrGa2	129 379 987	98.45	2 114 798	503 898 866	0.4196870	0.3770105
	DlabrGa3	118 863 303	98.02	2 082 274	503 524 710	0.4135396	0.388246
	DlabrGa4	125 269 167	98.02	2 085 977	503 675 656	0.4141508	0.380342
	DlabrGa5	121 308 016	98.05	2 093 356	503 693 457	0.4156012	0.3807775
	DlabrGa6	113 020 888	98.04	2 150 834	503 563 171	0.4271230	0.379656
S. pilchardus	SpilcFa1	98 789 082	96.42	7 128 735	604 856 771	1.178582	1.331
	SpilcFa3	109 782 816	96.24	7 452 162	613 645 048	1.214409	1.340575
	SpilcFa4	97 444 013	95.07	7 195 048	609 516 704	1.180451	1.347235
	SpilcFa5	110 357 654	95.67	7 545 357	615 252 143	1.226385	1.40959
	SpilcFa6	104 223 183	95.90	7 500 848	615 157 704	1.219337	1.41341
	SpilcMu1	124 885 493	96.20	7 471 544	617 558 276	1.209852	1.500415
	SpilcMu2	109 948 539	95.70	7 272 037	615 199 861	1.182061	1.474785
	SpilcMu3	108 564 997	92.46	7 272 929	614 357 522	1.183827	1.537
	SpilcMu4	122 541 302	95.47	7 283 499	616 999 758	1.180470	1.446285
	SpilcMu6	105 613 487	96.25	7 172 809	614 547 029	1.167170	1.40057
	SpilcLi2	121 934 021	95.06	7 123 340	614 907 641	1.158441	1.373815
	SpilcLi3	126 015 301	95.03	7 581 636	617 017 545	1.228755	1.417255
	SpilcLi4	116 445 615	95.68	7 350 852	615 226 870	1.194820	1.37415
	SpilcLi5	119 470 404	95.48	7 097 138	612 517 142	1.158684	1.336275
	SpilcLi6	112 805 605	96.10	6 835 691	611 663 555	1.117557	1.279095
	SpilcGa1	99 664 874	93.16	7 195 528	612 305 296	1.175154	1.5632
	SpilcGa3	106 538 562	95.80	7 353 467	615 590 542	1.194539	1.65899
	SpilcGa4	97 977 975	90.70	6 894 734	609 669 475	1.130897	1.895515
	SpilcGa5	102 032 394	95.10	7 137 835	612 596 676	1.165177	1.592515
	SpilcGa6	97 318 612	87.45	7 129 996	611 349 731	1.166271	1.97076

Dataset	Predictor	*p-value*			Pseudo R^2			Slope estimate (± 95% interval)		
		All	Med.	Atl.	All	Med.	Atl.	All.	Med.	Atl.
Whole data set	Body size	0.119	0.105	0.159	0.192	0.206	0.169	−0.006 (−0.014; 0.002)	−0.006 (−0.014; 0.001)	−0.005 (−0.013; 0.002)
	Trophic level	0.676	0.671	0.672	0.012	0.012	0.012	−0.091 (−0.524; 0.343)	−0.094 (−0.536; 0.349)	−0.090 (−0.516; 0.336)
	Propagule size	0.562	0.608	0.450	0.015	0.012	0.032	−0.014 (−0.062; 0.034)	−0.013 (−0.061; 0.036)	−0.018 (−0.066; 0.030)
	Fecundity	0.653	0.605	0.723	0.013	0.017	0.008	$-1.22e^{-5}$ ($-6.63e^{-5}; 4.20e^{-5}$)	$-1.44e^{-5}$ ($-7.00e^{-5}; 4.13e^{-5}$)	$-9.2e^{-6}$ ($-6.18e^{-5}; 4.35e^{-5}$)
	Lifespan	**0.0107**	**0.0085**	**0.0219**	0.438	0.453	0.404	−0.062 (−0.111; −0.013)	−0.0652 (−0.115; −0.016)	−0.056 (−0.104; −0.007)
	Adult lifespan	**0.0070**	**0.0055**	**0.0216**	0.429	0.444	0.374	−0.089 (−0.156; −0.023)	−0.093 (−0.161; −0.026)	−0.077 (−0.144; −0.0010)
	Hermaphroditism	0.434	0.465	0.350	0.034	0.03	0.050	0.1779 (−0.278; 0.633)	0.1701 (−0.296; 0.636)	0.2069 (−0.2359; 0.6497)
	Parental Care	0.274	0.287	0.168	0.075	0.071	0.121	−0.273 (−0.772; 0.226)	−0.271 (−0.781; 0.239)	−0.335 (−0.822; 0.152)
No parental care	Body size	$\mathbf{6.60e^{-5}}$	$\mathbf{3.85e^{-5}}$	$\mathbf{8.17e^{-5}}$	0.616	0.636	0.607	−0.014 (−0.021; −0.007)	−0.014 (−0.021; −0.007)	−0.014 (−0.021; −0.007)
	Trophic level	0.256	0.245	0.254	0.093	0.097	0.095	−0.326 (−0.902; 0.251)	−0.340 (−0.925; 0.244)	−0.328 (−0.903; 0.247)
	Propagule size	0.170	0.155	0.200	0.175	0.184	0.157	−0.518 (−1.273; 0.237)	−0.544 (−1.310; 0.221)	−0.484 (−1.240; 0.272)
	Fecundity	0.390	0.353	0.401	0.056	0.065	0.054	$-2.51e^{-5}$ ($-8.35e^{-5}; 3.33e^{-5}$)	$-2.76e^{-5}$ ($-8.71e^{-5}; 3.19e^{-5}$)	$-2.44e^{-5}$ ($-8.26e^{-5}; 3.37e^{-5}$)
	Lifespan	$\mathbf{1.017e^{-7}}$	$\mathbf{7.28e^{-8}}$	$\mathbf{5.451e^{-7}}$	0.851	0.856	0.832	−0.095 (−0.131; −0.060)	−0.097 (−0.134; −0.061)	−0.094 (−0.131; −0.056)
	Adult lifespan	$\mathbf{1.65e^{-7}}$	$\mathbf{1.36e^{-7}}$	$\mathbf{8.778e^{-7}}$	0.829	0.837	0.805	−0.129 (−0.179; −0.080)	−0.131 (−0.181; −0.081)	−0.127 (−0.178; −0.075)
	Hermaphroditism	0.454	0.407	0.489	0.044	0.053	0.038	0.206 (−0.345; 0.757)	0.231 (−0.326; 0.787)	0.191 (−0.360; 0.741)

Table S3: **Statistical relationships between different estimations of species genetic diversity and life history traits** - Genetic diversity, either estimated from all individuals (all), individuals from Mediterranean Sea (Med.), Atlantic Ocean (Atl.) was fitted to 6 quantitative (body size, trophic level, propagule size, fecundity, lifespan and adult lifespan) and two qualitative predictors (hermaphroditism and parental care) with a beta regression model using the `betareg` R package (Zeileis and Hothorn, 2002). In the upper part of the table, regressions were performed with the whole dataset, while in the lower part only the 11 non-brooding species were considered.

Additional figures

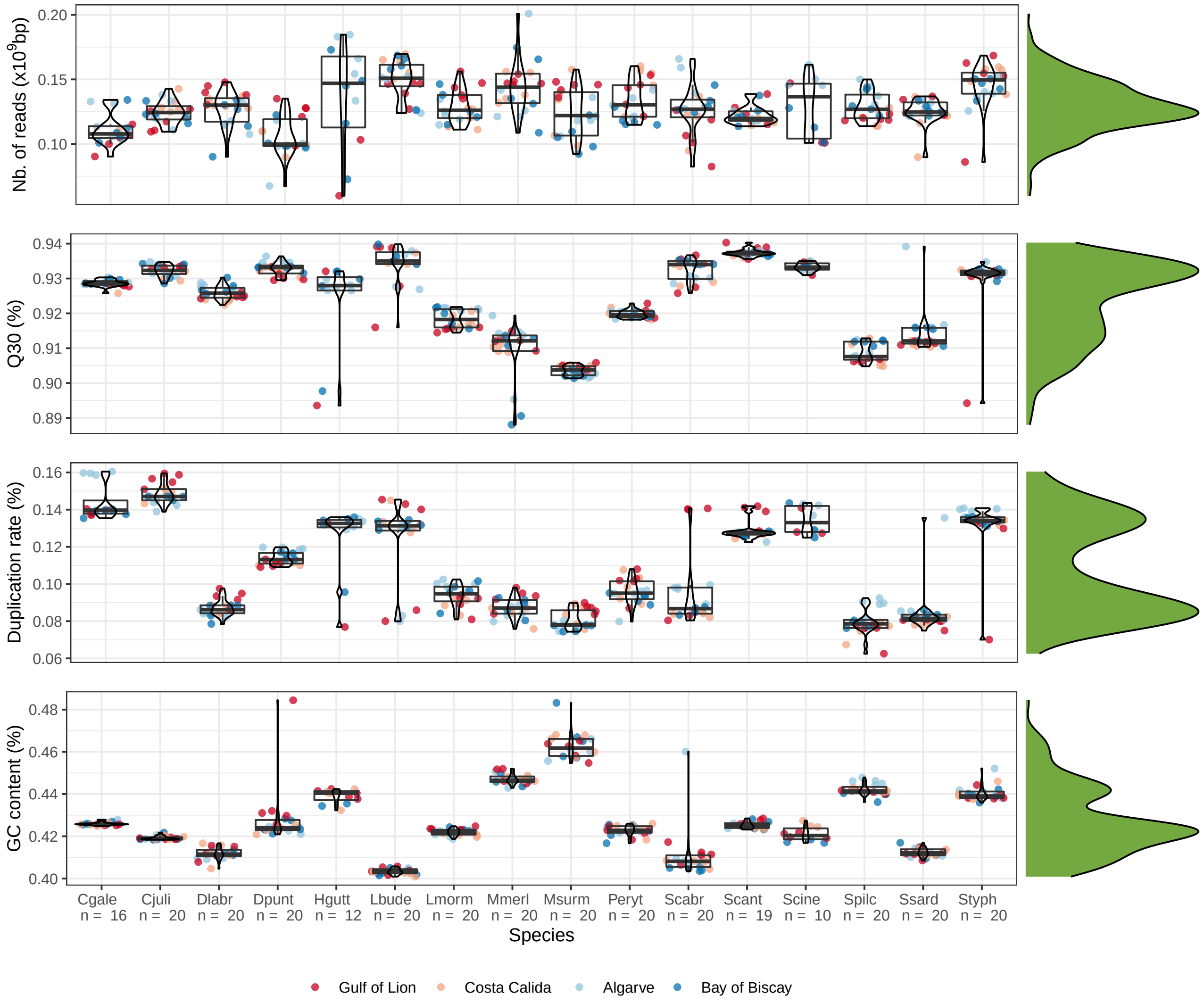

Figure S2: **Number of reads (10^9 bp), percentage of reads with quality superior to Q30, duplication rate and GC content after filtering, correcting and trimming steps carried out with `fastp` v.0.20.0 (Chen et al., 2018)**. Each point represents an individual unassembled genome: species are represented on the y-axis. Colors represent sampling locations: mediterranean locations in warm colors (Gulf of Lion in dark red, Murcia in pale red), atlantic locations in cold colors (Faro in pale blue, Bay of Biscay in dark blue). Overall distributions of each parameters are represented on the right side of each panel. Cgale = *Coryphoblennius galerita*, Cjuli = *Coris julis*, Dlabr = *Dicentrarchus labrax*, Dpunt = *Diplodus puntazzo*, Hgutt = *Hippocampus guttulatus*, Lbude = *Lophius budegassa*, Lmorm = *Lithognathus mormyrus*, Mmerl = *Merluccius merluccius*, Msurm = *Mullus surmuletus*, Peryt = *Pagellus erythrinus*, Scabr = *Serranus cabrilla*, Scant = *Spondyliosoma cantharus*, Scine = *Symphodus cinereus*, Spilc = *Sardina pilchardus*, Ssard = *Sarda sarda*, Styph = *Syngnathus typhle*.

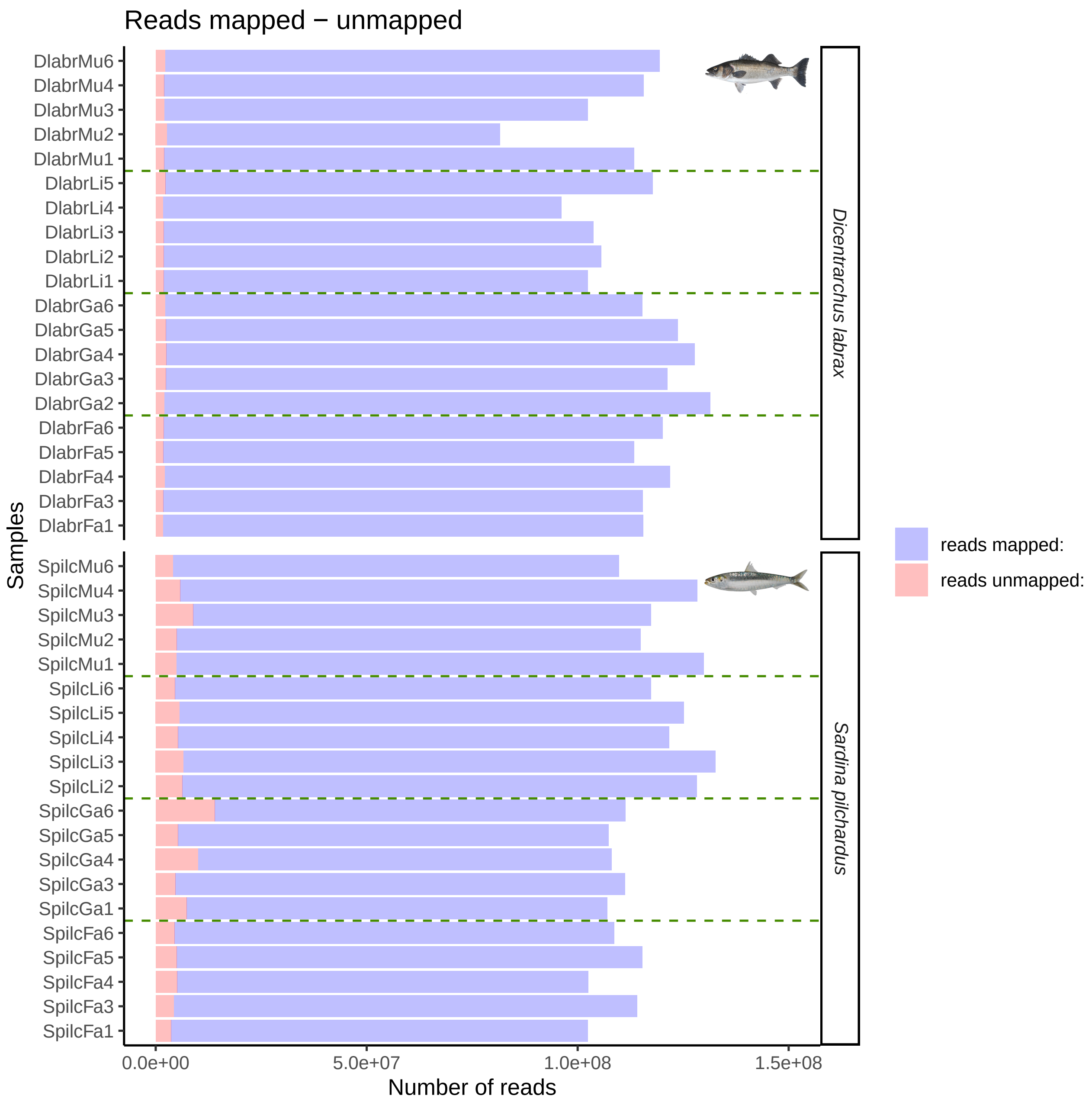

Figure S3: **Mapping statistics** - Number of reads mapped in red and unmapped blue with `bwa` v.0.7.17 for the 20 indiviuals of *D. labrax* and *S. pilchardus*.

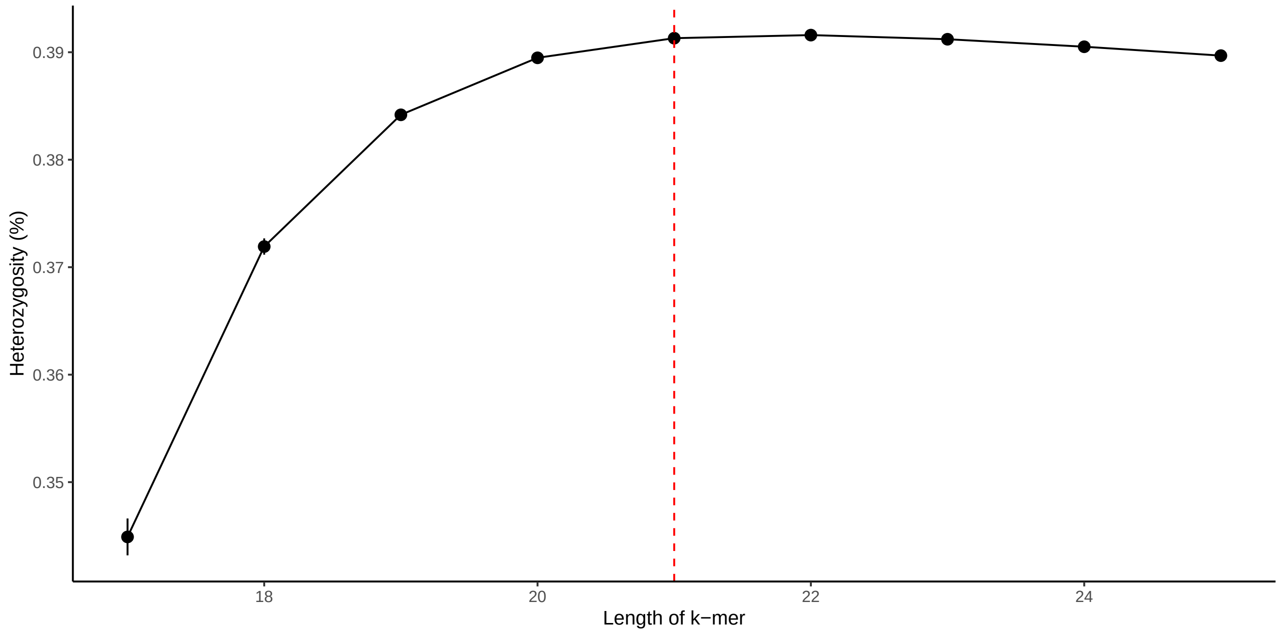

Figure S4: **Effect of $k - mer$ length on genetic diversity estimation with `GenomeScope` v.1.0 (Vurture et al., 2017)** - Genetic diversity was estimated with `GenomeScope` with different values of $k - mer$ length (17,19,21,23 and 25) on one European sea bass individual (*D. labrax*), all other parameters being equal (as detailed in the main text). The red vertical dashed line represents the value used in this study (21).

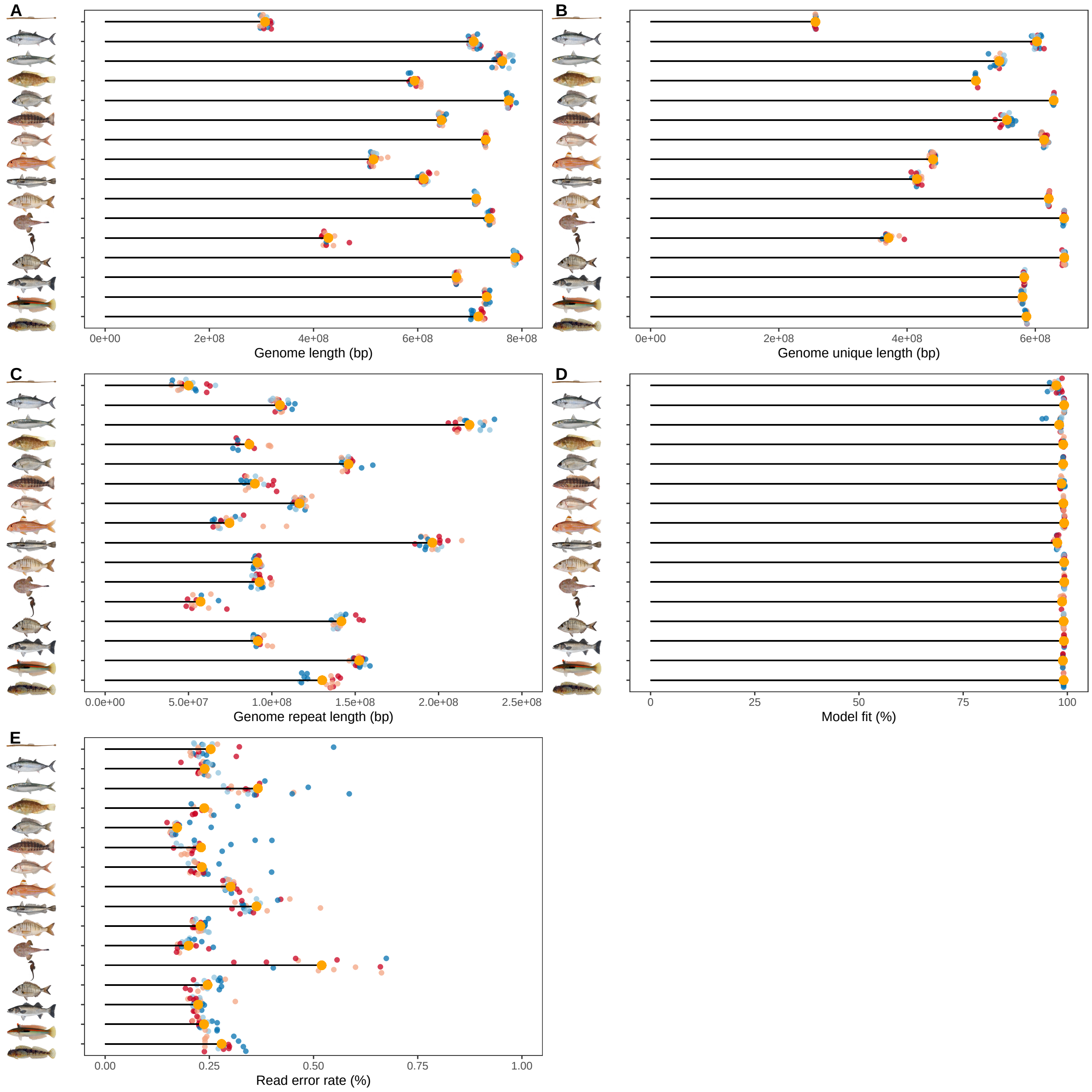

Figure S5: **Individual whole-genome sequences features estimated with** `GenomeScope` **v.1.0 (Vurture et al., 2017)**. A) Genome length (number of base pairs), B) genome unique length (number of base pairs), C) genome repeat length (number of base pairs), D) read error rate (%) and E) model fit (%). Individual feature and species median feature is represented in yellow in each panel. Species illustrations were retrieved from Iglésias (2013) with permissions.

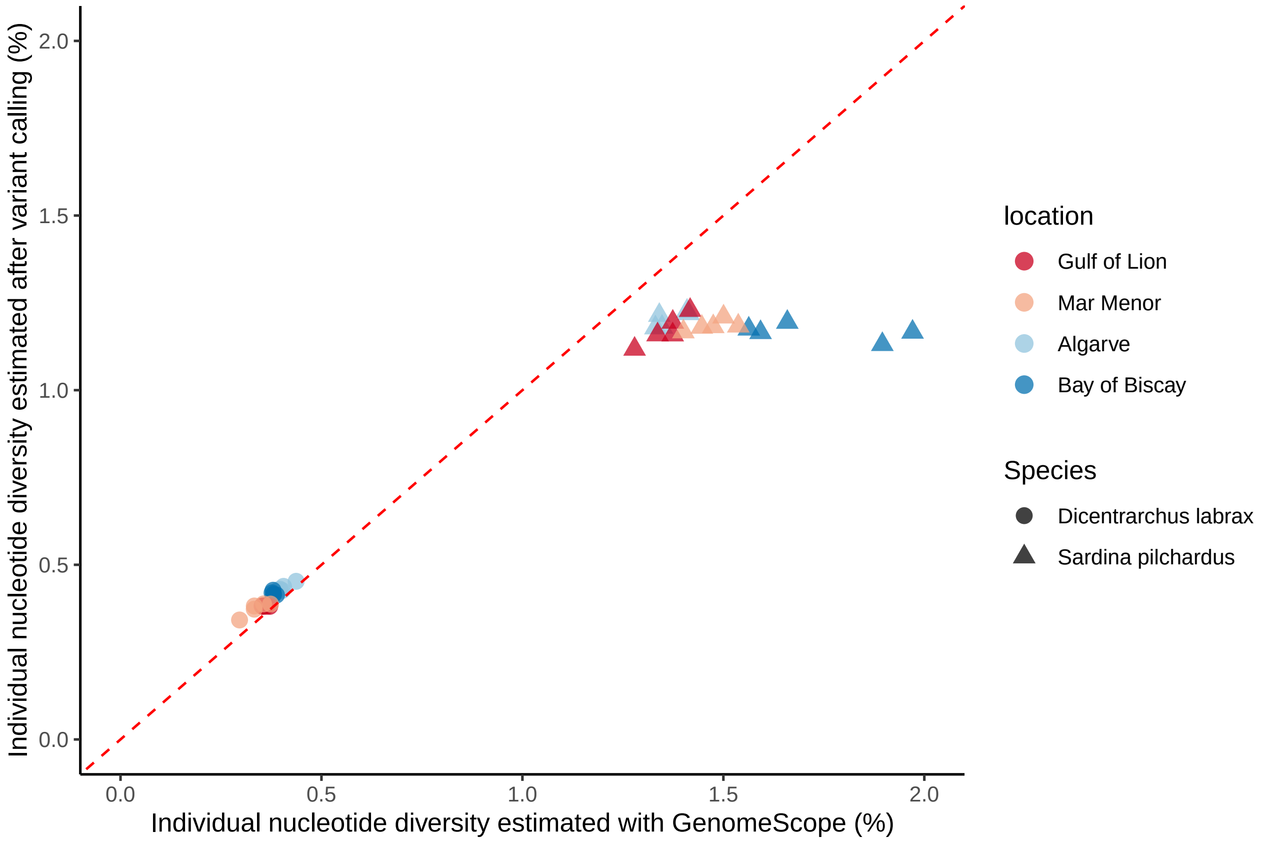

Figure S6: Relationship between individual mean genome-wide heterozygosity estimated with the k-mer based reference-free approach in GenomeScope (x-axis), and the high standard reference-based approach in GATK (y-axis), for european sea bass (*D. labrax*, dots) and european pilchard (*S. pilchardus*, triangle).

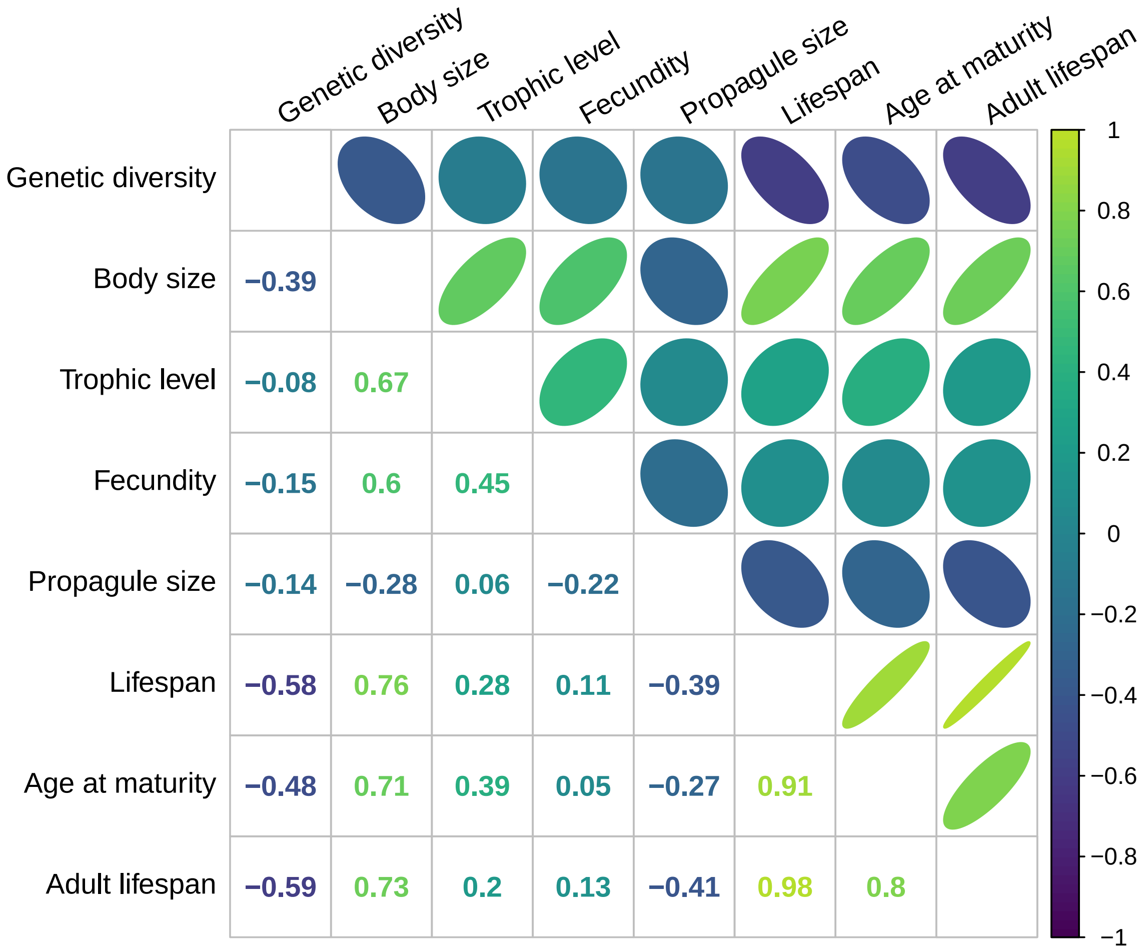

Figure S7: **Correlation matrix between genetic diversity and all quantitative life history traits** - Upper triangle represents the strength and the direction of the correlation, the lower triangle the coefficient of correlation.

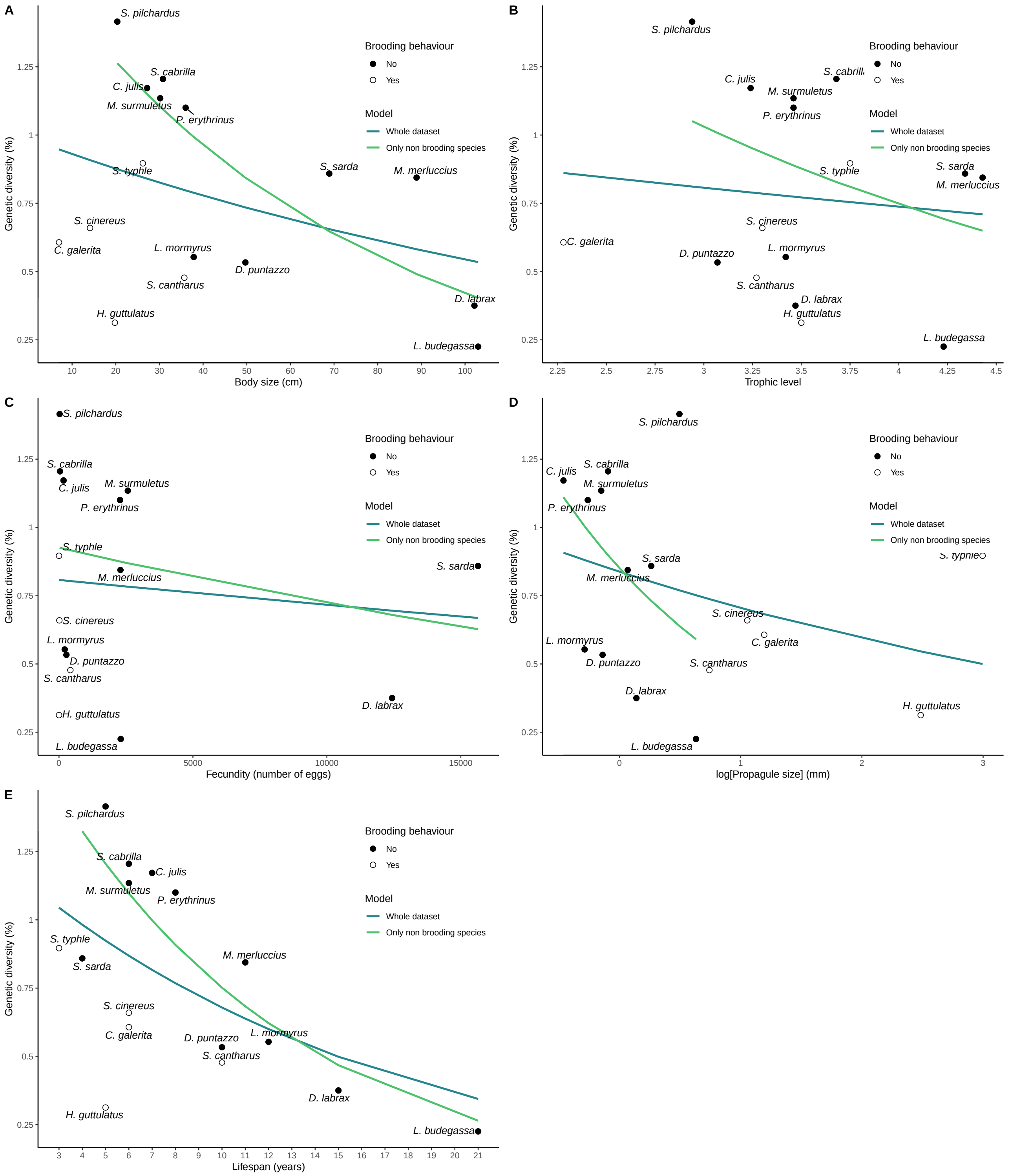

Figure S8: **Relationship between species median genetic diversity (%) and 5 covariables** - Each point represents the median of observed genetic diversity among individuals within each species. Full points represents non-brooding species, empty circles, brooding species. Blue and green line represent the beta regression between each predictive variable and genetic diversity considering either the whole dataset (16 species), or the 11 non-brooding species only, respectively. **A)** adult body size, **B)** trophic level, **C)** fecundity, **D)**, propagule size and **E)**, lifespan.

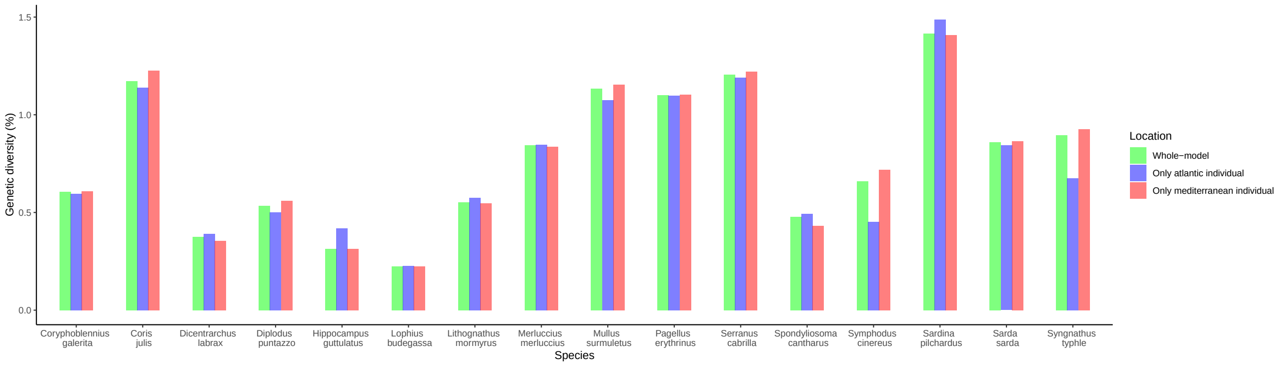

Figure S9: **Effect of population structure on genetic diversity estimates**. For each species on the x-axis, genetic diversity is estimate from the median of individual genome-wide heterozygosities for all the individuals (in green), from the individuals from the Mediterranean Sea (in red) or from the individuals from the Atlantic Ocean (in blue).

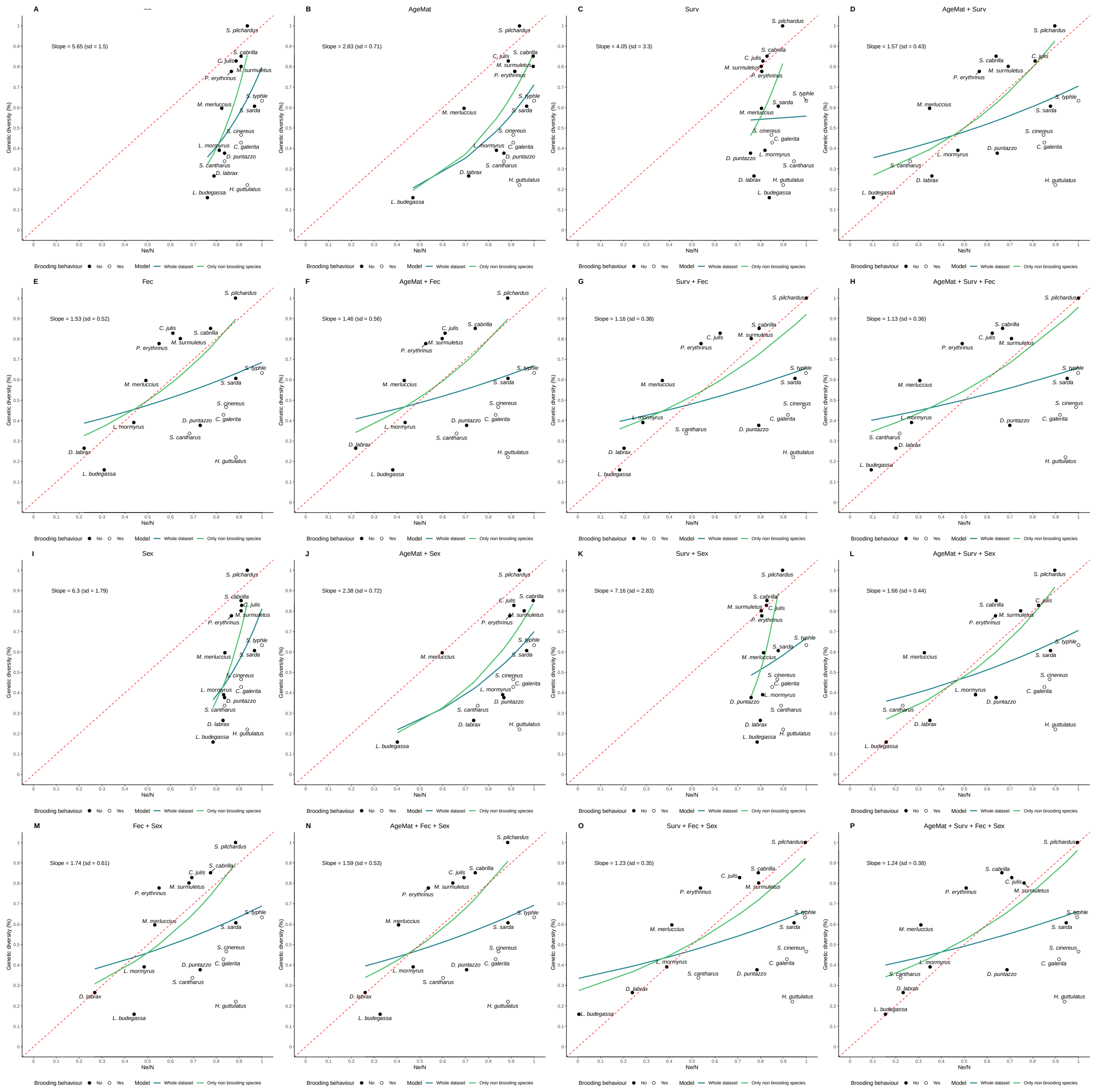

Figure S10: **Relationship between relative species genetic diversity and relative $\frac{N_e}{N}$ estimated by AgeNe for 16 sets of life tables.** Estimated species genetic diversity divided by the highest empirical estimates (i.e. *Sardina pilchardus*) on y-axis, and $\frac{N_e}{N}$ estimated by AgeNe divided by the highest estimates in each of the 16 sets of life tables. Each of the 16 panels represents the impact of species life tables considering some characteristics: AgeMat (delayed first age at maturity), Surv (age-specific survival estimated from empirical species age-length distributions), Fec (empirical estimates of increasing fecundity with age), Sex (sex-specific differences in either, age at first maturity, age-specific survival or/and fecundity). A) Age at first maturity at 1 year old; constant age-specific survival; constant age-specific fecundity and no differences between sex life tables, B) Species-specific age at first maturity; constant age-specific survival; constant age-specific fecundity and no differences between sex life tables, C) Age at first maturity at 1 year old; increasing age-specific survival rate; D) Species-specific age at first maturity; increasing age-specific survival rate; constant age-specific fecundity and no differences between sex life tables, constant age-specific fecundity and no differences between sex life tables, E) Age at first maturity at 1 year old; constant age-specific survival; increasing fecundity with age and no differences between sex life tables, F) Species-specific age at first maturity; constant age-specific survival; increasing fecundity with age and no differences between sex life tables, G) Age at first maturity at 1 year old; increasing age-specific survival rate; increasing fecundity with age and no differences between sex life tables, H) Species-specific age at first maturity; increasing age-specific survival rate; increasing fecundity with age and no differences between sex life tables, I) Age at first maturity at 1 year old; constant age-specific survival; constant age-specific fecundity and sex-specific differences between life tables, J) Species-specific age at first maturity; constant age-specific survival; constant age-specific fecundity and sex-specific differences between life tables, K) Age at first maturity at 1 year old; increasing age-specific survival rate; constant age-specific fecundity and sex-specific differences between life tables, L) Species-specific age at first maturity; increasing age-specific survival rate; constant age-specific fecundity and sex-specific differences between life tables, M) Age at first maturity at 1 year old; constant age-specific survival; increasing fecundity with age and sex-specific differences between life tables, N) Species-specific age at first maturity; constant age-specific survival; increasing fecundity with age and sex-specific differences between life tables, O) Age at first maturity at 1 year old; increasing age-specific survival rate; increasing fecundity with age and sex-specific differences between life tables, P) Species-specific age at first maturity; increasing age-specific survival rate; increasing fecundity with age and sex-specific differences between life tables. Full points represents non-brooding species, empty points, brooding species. Dashed blue line and solid green represent the beta regression between relative genetic diversity and relative estimated $\frac{N_e}{N}$. Dotted red line represents the identity curve, with slope equals 1 and intercept 0

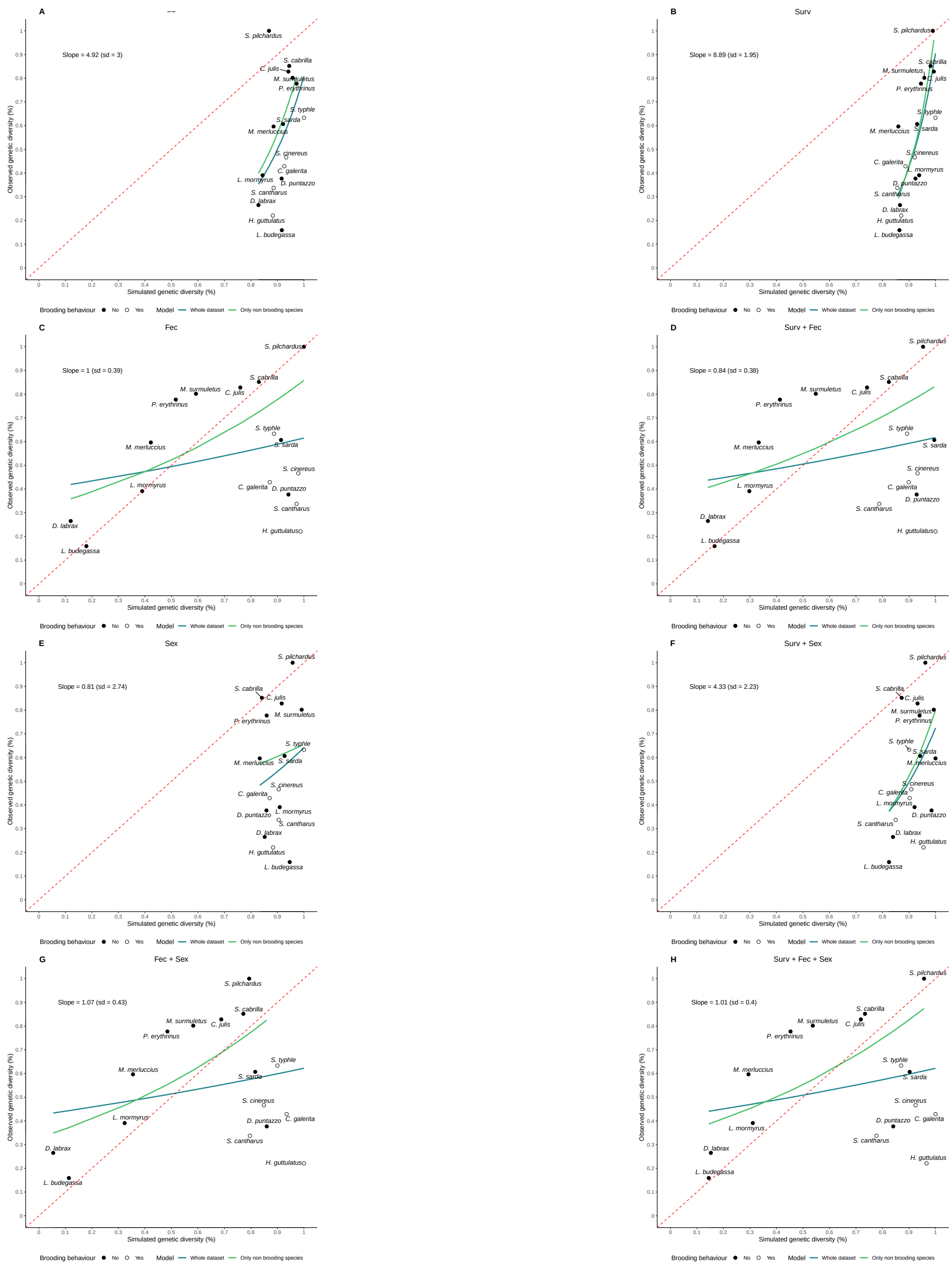

Figure S11: **Relationship between relative species genetic diversity and simulated genetic diversity with forward-in-time simulations for 16 sets of life tables**. Estimated species genetic diversity divided by the highest empirical estimates (i.e. *Sardina pilchardus*) on *y*-axis, and simulated genetic diversity estimated by `SLiM` v.3.3.1 (Haller and Messer, 2017) divided by the highest estimates in each of the 16 set of life tables. Each of the 16 panels represents species life tables considering some characteristics: AgeMat (delayed first age of maturity), Surv (age-specific survival estimated from empirical species age-length distributions), Fec (empirical estimates of increasing fecundity with age), Sex (sex-specific differences in either, age at first maturity, age-specific survival or/and fecundity). A) Age at first maturity at 1 year old; constant age-specific survival; constant age-specific fecundity and no differences between sex life tables, B) Age at first maturity at 1 year old; increasing age-specific survival rate; C) Age at first maturity at 1 year old; constant age-specific survival; increasing fecundity with age and no differences between sex life tables, D) Age at first maturity at 1 year old; increasing age-specific survival ratel; increasing fecundity with age and no differences between sex life tables, E) Age at first maturity at 1 year old; constant age-specific survival; constant age-specific fecundity and sex-specific differences between life tables, F) Age at first maturity at 1 year old; increasing age-specific survival rate; constant age-specific fecundity and sex-specific differences between life tables, G) Age at first maturity at 1 year old; constant age-specific survival; increasing fecundity with age and sex-specific differences between life tables, H) Age at first maturity at 1 year old; increasing age-specific survival rate; increasing fecundity with age and sex-specific differences between life tables, Full points represents non-brooding species, empty points, brooding species. Dashed blue line and solid green represent the beta regression between relative genetic diversity and relative estimated $\frac{N_e}{N}$. Dotted red line represents the identity curve, with slope equals 1 and intercept 0

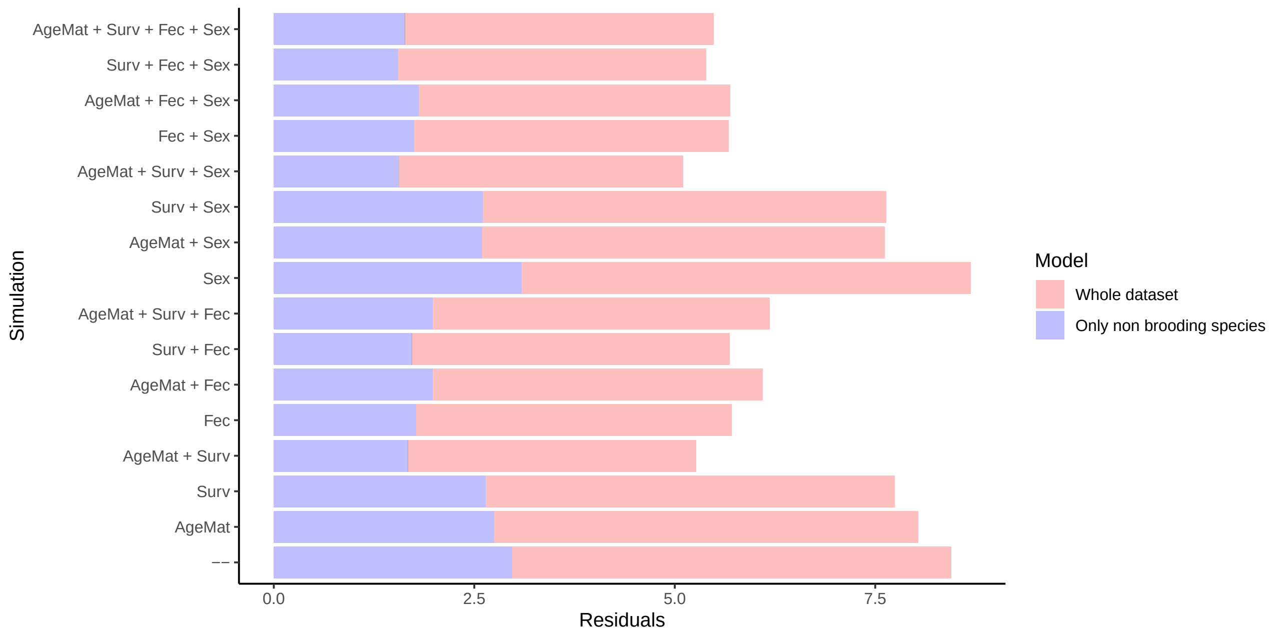

Figure S12: **Residuals of the linear model between genetic diversity and variance in reproductive success estimated from various combinations of life tables from a model with slope equals 1 and intercept 0**. For each set of life tables, we estimated $\frac{N_e}{N}$ with `AgeNe` and calculated the sum of squared deviation between these estimate and a model with slope equals 1 and intercept 0, as residuals. Higher residual number means variation in $\frac{N_e}{N}$ explain little the variation of observed genetic diversity.

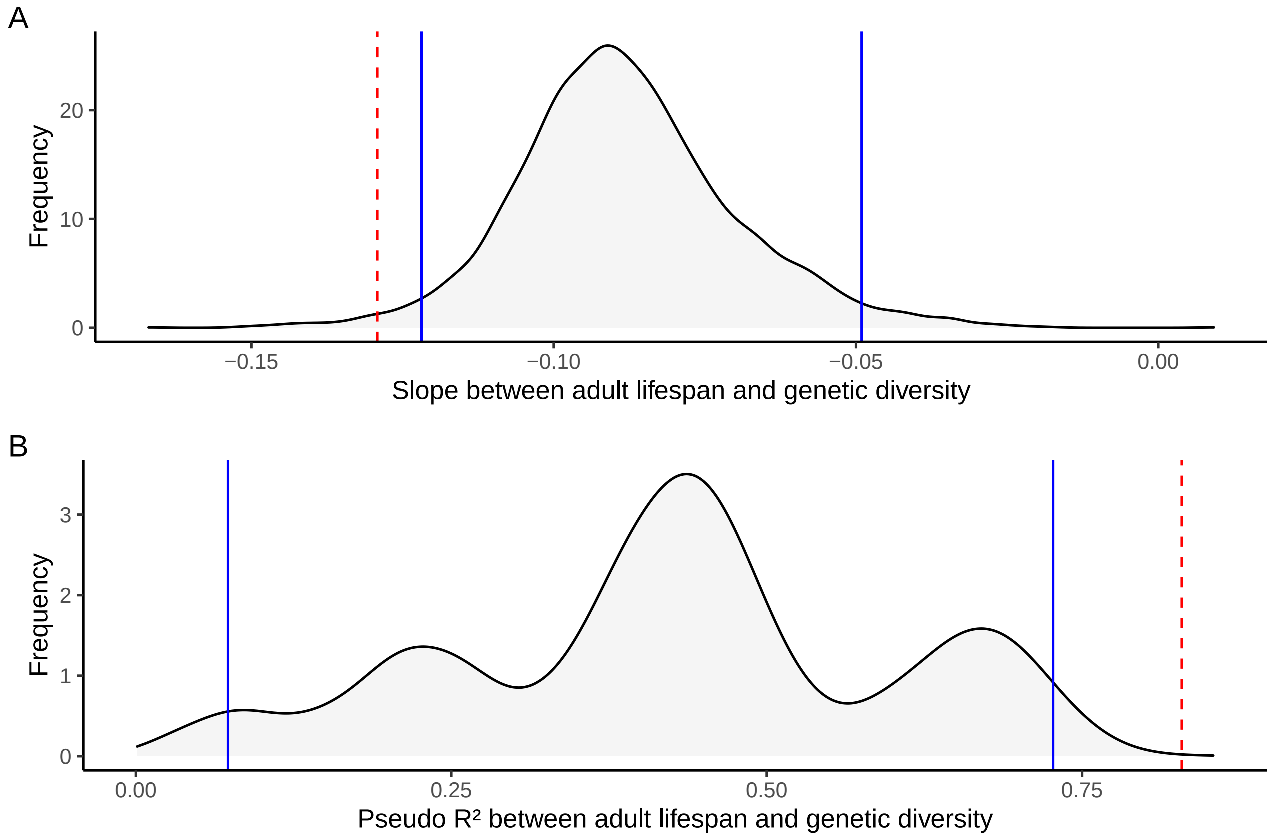

Figure S13: **Distribution of slope and pseudo R^2 of the beta regression between adult lifespan and genetic diversity for random subsets of 11 species** - We fit a beta regression between adult lifespan and genetic diversity for all sub-samples of 11 species and calculated the width of the 95% interval of the slope (A) and pseudo R^2 (B), represented with solid blue lines. Dashed red lines represent the estimated slope and pseudo R^2 for the sub-sample of the 11 species with no parental care behaviour. In (A) the 95% width interval ranged from -0.122 to -0.049, and the estimated slope for the non-brooders only was equal to -0.129. In (B), the 95% width interval ranged from 0.073 to 0.727, and the estimated pseudo R^2 for the non-brooders only was equal to 0.829.

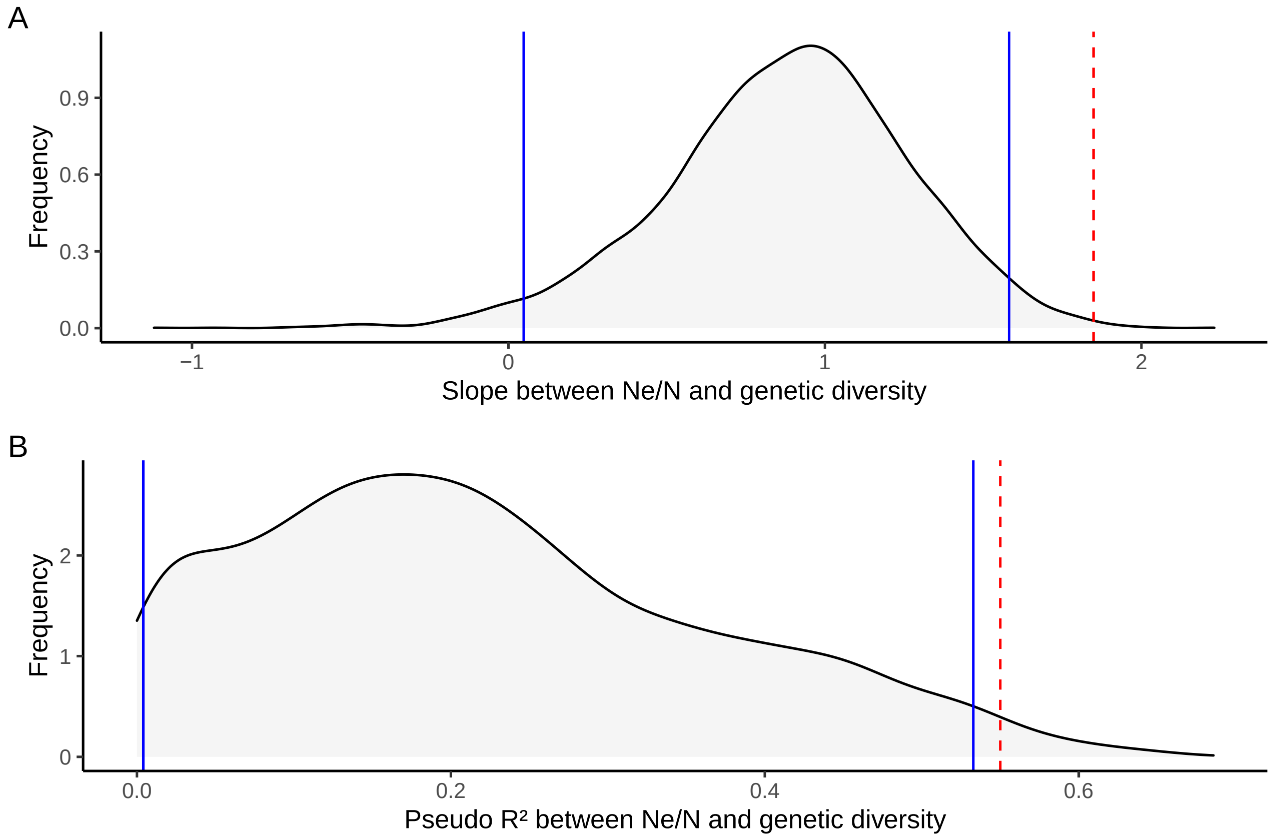

Figure S14: **Distribution of slope and pseudo R^2 of the beta regression between $\frac{N_e}{N}$ and genetic diversity for random subsets of 11 species** - We fit a beta regression between observed genetic diversity and $\frac{N_e}{N}$ for all sub-samples of 11 species and calculated the width of the 95% interval of the slope (A) and pseudo R^2 (B), represented with solid blue lines. Dashed red lines represent the estimated slope and pseudo R^2 for the sub-sample of the 11 species with no parental care behaviour. In (A) the 95% width interval ranged from 0.048 to 1.582, and the estimated slope for the non-brooders only was equal to 1.849. In (B), the 95% width interval ranged from 0.004 to 0.533, and the estimated pseudo R^2 from the non-brooders only was equal to 0.55.

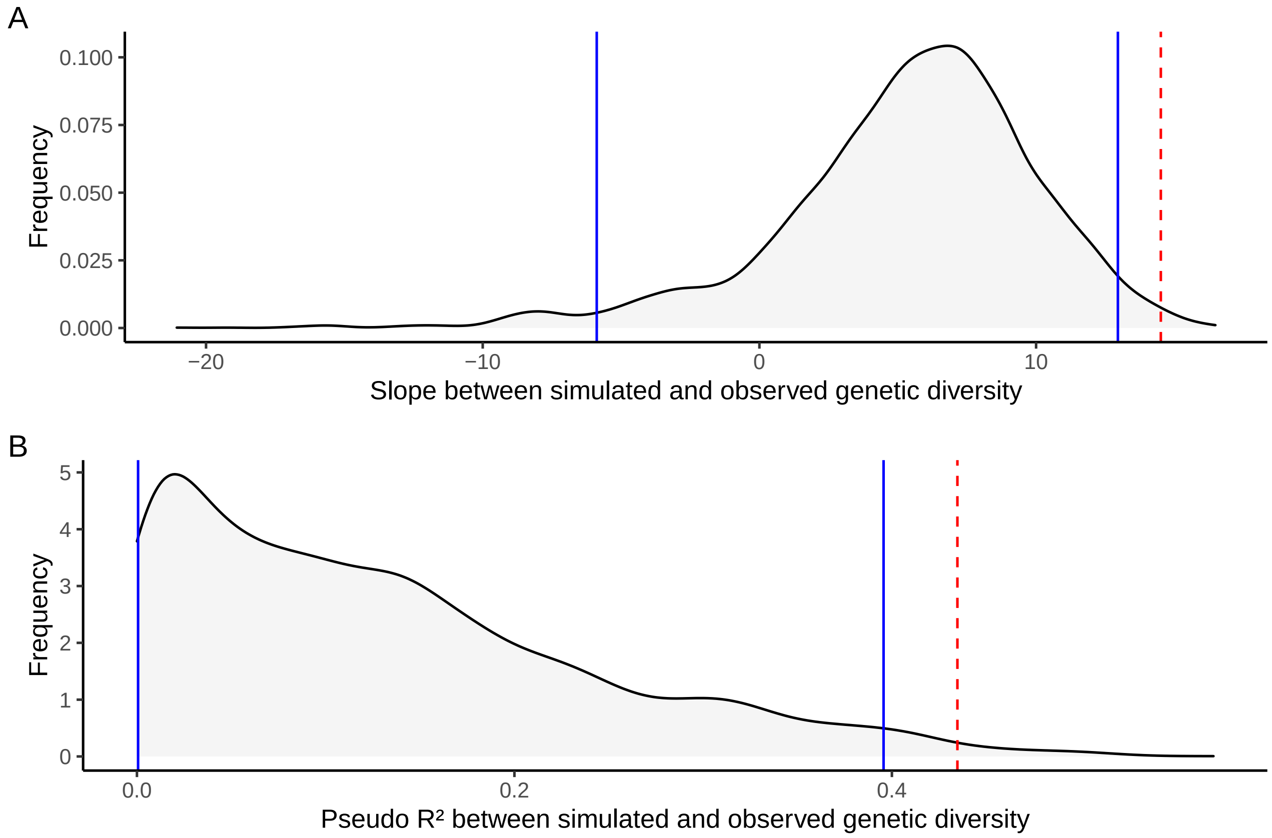

Figure S15: **Distribution of slope and pseudo R^2 of the beta regression between simulated, with `SLiM` v.3.3.1, and observed genetic diversity for random subset of 11 species** - We fit a beta regression between simulated genetic diversity and observed genetic diversity for all sub-samples of 11 species and calculated the width of the 95% interval of the slope (A) and pseudo R^2 (B), represented with solid blue lines. Dashed red lines represent the estimated slope and pseudo R^2 for the sub-sample of the 11 species with no parental care behaviour. In (A) the 95% width interval ranged from -5.879 to 12.957, and the estimated slope for the non-brooders only was equal to 14.509. In (B), the 95% width interval ranged from 0.001 to 0.396, and the estimated pseudo R^2 from the non-brooders only was equal to 0.435.

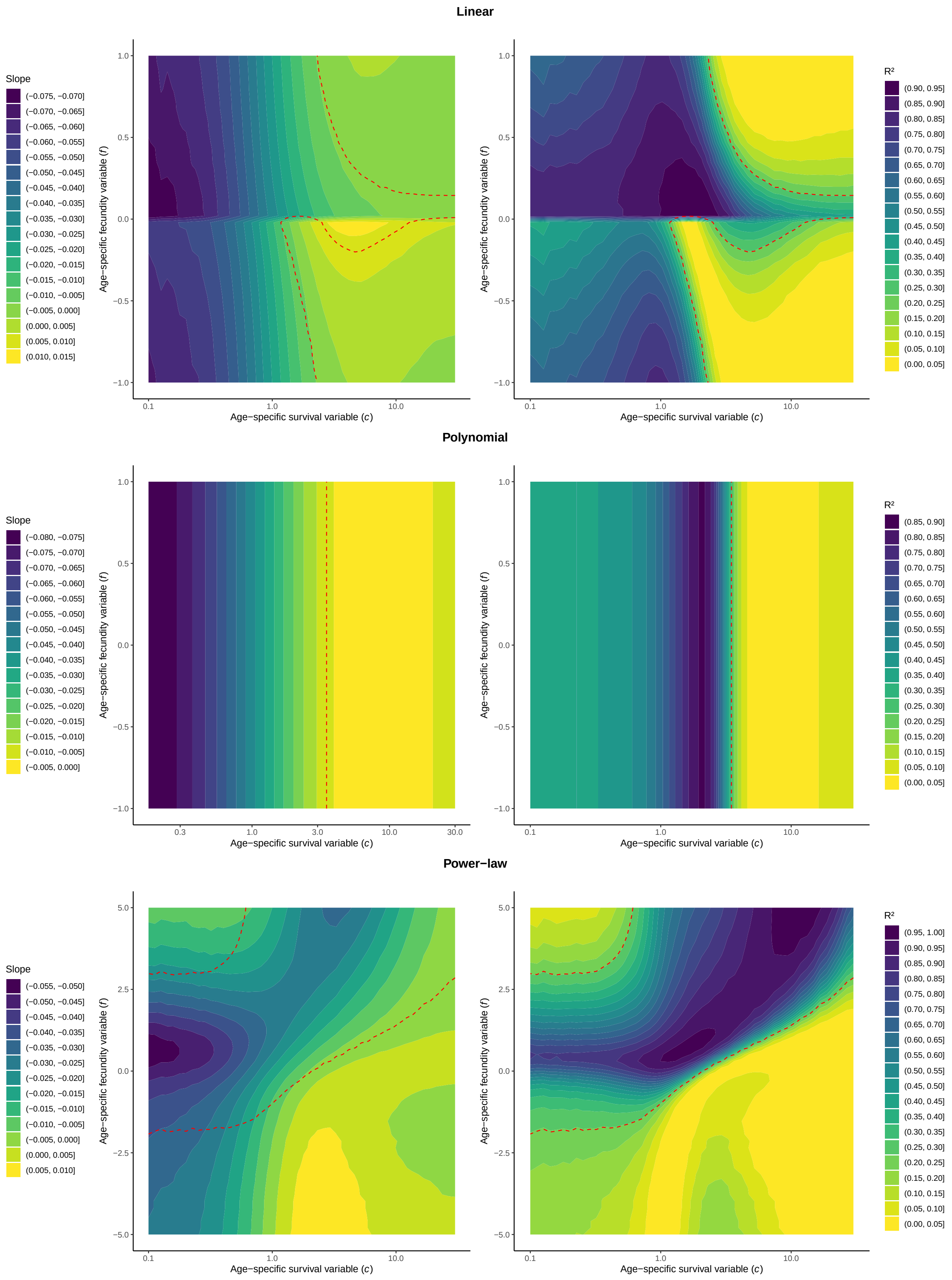

Figure S16: **Slope of and proportion of variance explained by linear models between adult lifespan and $\frac{N_e}{N}$ estimated with `AgeNe` for different combinations of age-specific survival and fecundity for three fecundity-age models: linear, polynomial and power-law**. Colder colors indicate steeper slope (left panels) or higher R^2 (right panels) of the regression between adult lifespan and $\frac{N_e}{N}$. On top, linear fecundity-age model ($F = \alpha \times L + \beta$), middle, polynomial fecundity-age model and on the bottom, power-law model, ($F = \alpha L^{\beta}$)

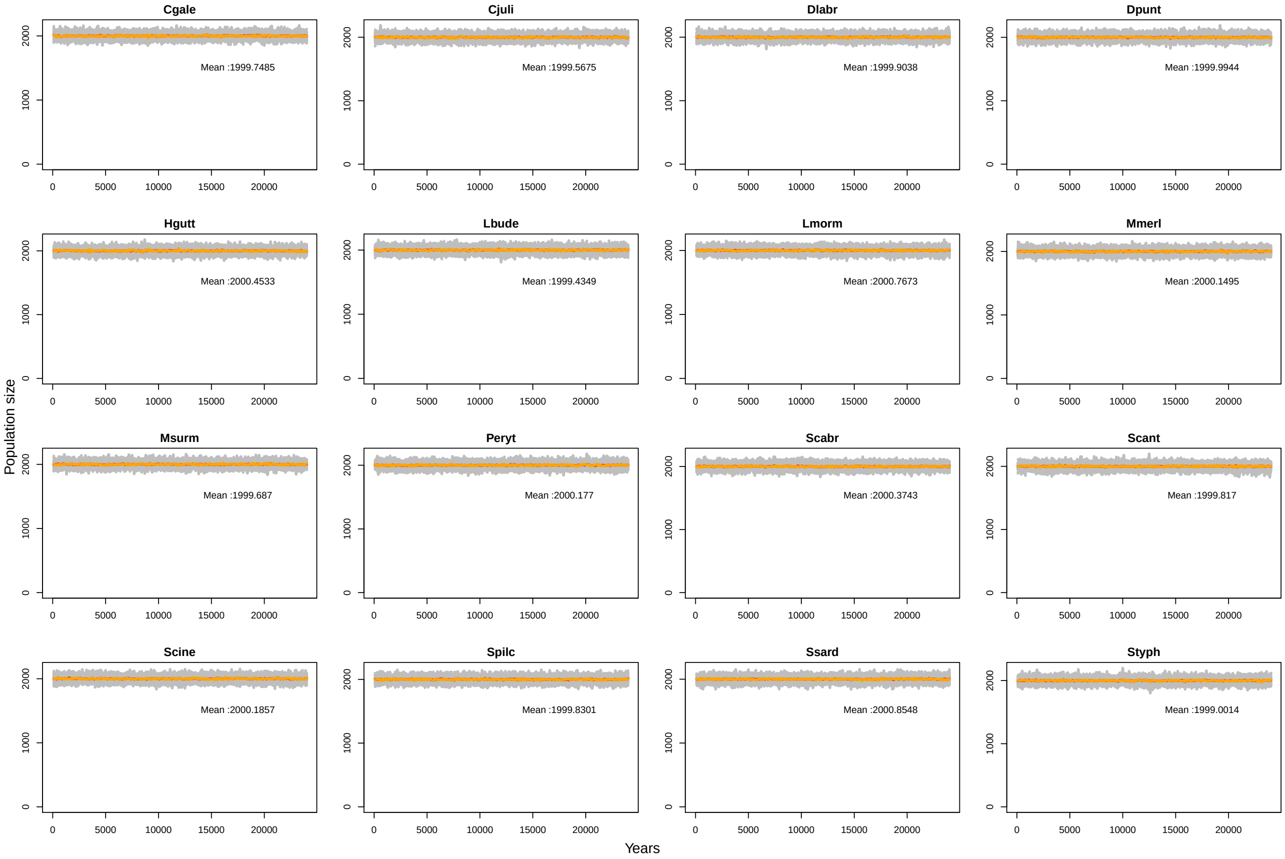

Figure S17: **Population size count for the 50 iterations of the 16 species for set 1 of life tables (age at first maturity at 1 year old, constant age-specific survival rate, constant age-specific fecundity and no differences between sex-specific life tables)**. Adult population size was count for each iteration every 100 years during the 25000 years of the simulation. Each grey line represents population size fluctuations for one iteration. For each species, orange and red line, respectively, represents the median and the mean for all the 50 iterations. Mean over all 50 iterations is written on the plot for each species. Cgale = *Coryphoblennius galerita*, Cjuli = *Coris julis*, Dlabr = *Dicentrarchus labrax*, Dpunt = *Diplodus puntazzo*, Hgutt = *Hippocampus guttulatus*, Lbude = *Lophius budegassa*, Lmorm = *Lithognathus mormyrus*, Mmerl = *Merluccius merluccius*, Msurm = *Mullus surmuletus*, Peryt = *Pagellus erythrinus*, Scabr = *Serranus cabrilla*, Scant = *Spondyliosoma cantharus*, Scine = *Symphodus cinereus*, Spilc = *Sardina pilchardus*, Ssard = *Sarda sarda*, Styph = *Syngnathus typhle*

.

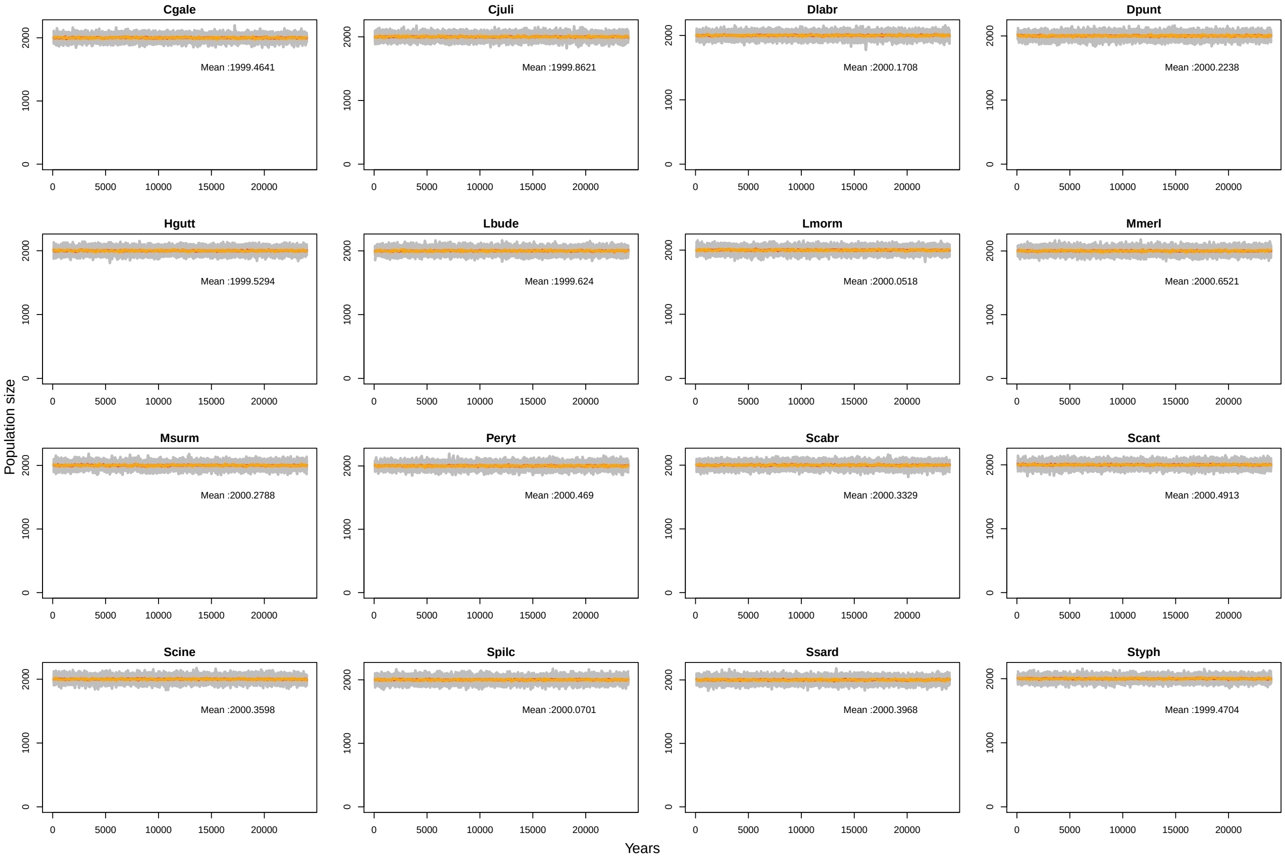

Figure S18: **Population size count for the 50 iterations of the 16 species for set 2 of life tables (age at first maturity at 1 year old, increasing age-specific survival rate, constant age-specific fecundity and no differences between sex-specific life tables)**. Adult population size was count for each iteration every 100 years during the 25000 years of the simulation. Each grey line represents population size fluctuations for one iteration. For each species, orange and red line, respectively, represents the median and the mean for all the 50 iterations. Mean over all 50 iterations is written on the plot for each species. Cgale = *Coryphoblennius galerita*, Cjuli = *Coris julis*, Dlabr = *Dicentrarchus labrax*, Dpunt = *Diplodus puntazzo*, Hgutt = *Hippocampus guttulatus*, Lbude = *Lophius budegassa*, Lmorm = *Lithognathus mormyrus*, Mmerl = *Merluccius merluccius*, Msurm = *Mullus surmuletus*, Peryt = *Pagellus erythrinus*, Scabr = *Serranus cabrilla*, Scant = *Spondyliosoma cantharus*, Scine = *Symphodus cinereus*, Spilc = *Sardina pilchardus*, Ssard = *Sarda sarda*, Styph = *Syngnathus typhle*

.

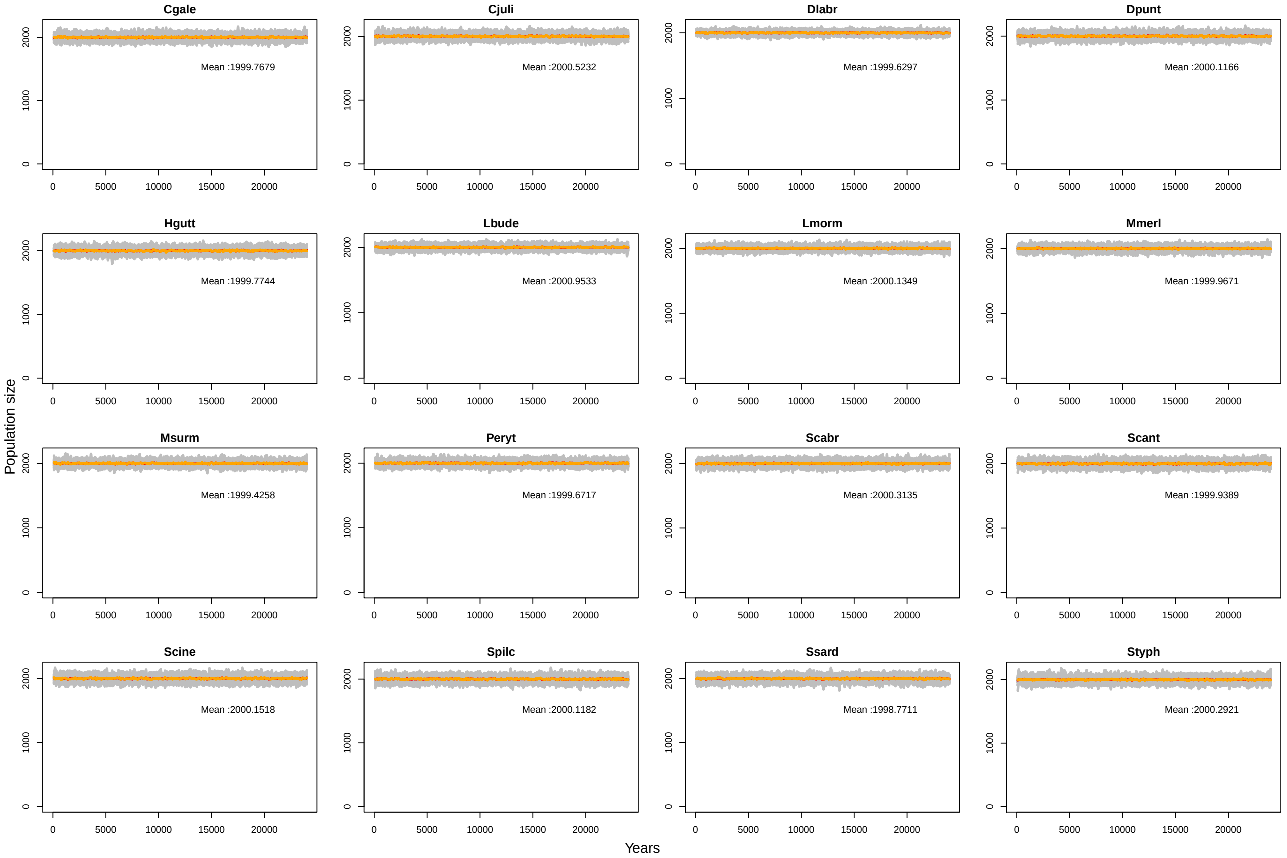

Figure S19: **Population size count for the 50 iterations of the 16 species for set 3 of life tables (age at first maturity at 1 year old, constant age-specific survival rate, increasing age-specific fecundity and no differences between sex-specific life tables)**. Adult population size was count for each iteration every 100 years during the 25000 years of the simulation. Each grey line represents population size fluctuations for one iteration. For each species, orange and red line, respectively, represents the median and the mean for all the 50 iterations. Mean over all 50 iterations is written on the plot for each species. Cgale = *Coryphoblennius galerita*, Cjuli = *Coris julis*, Dlabr = *Dicentrarchus labrax*, Dpunt = *Diplodus puntazzo*, Hgutt = *Hippocampus guttulatus*, Lbude = *Lophius budegassa*, Lmorm = *Lithognathus mormyrus*, Mmerl = *Merluccius merluccius*, Msurm = *Mullus surmuletus*, Peryt = *Pagellus erythrinus*, Scabr = *Serranus cabrilla*, Scant = *Spondyliosoma cantharus*, Scine = *Symphodus cinereus*, Spilc = *Sardina pilchardus*, Ssard = *Sarda sarda*, Styph = *Syngnathus typhle*

.

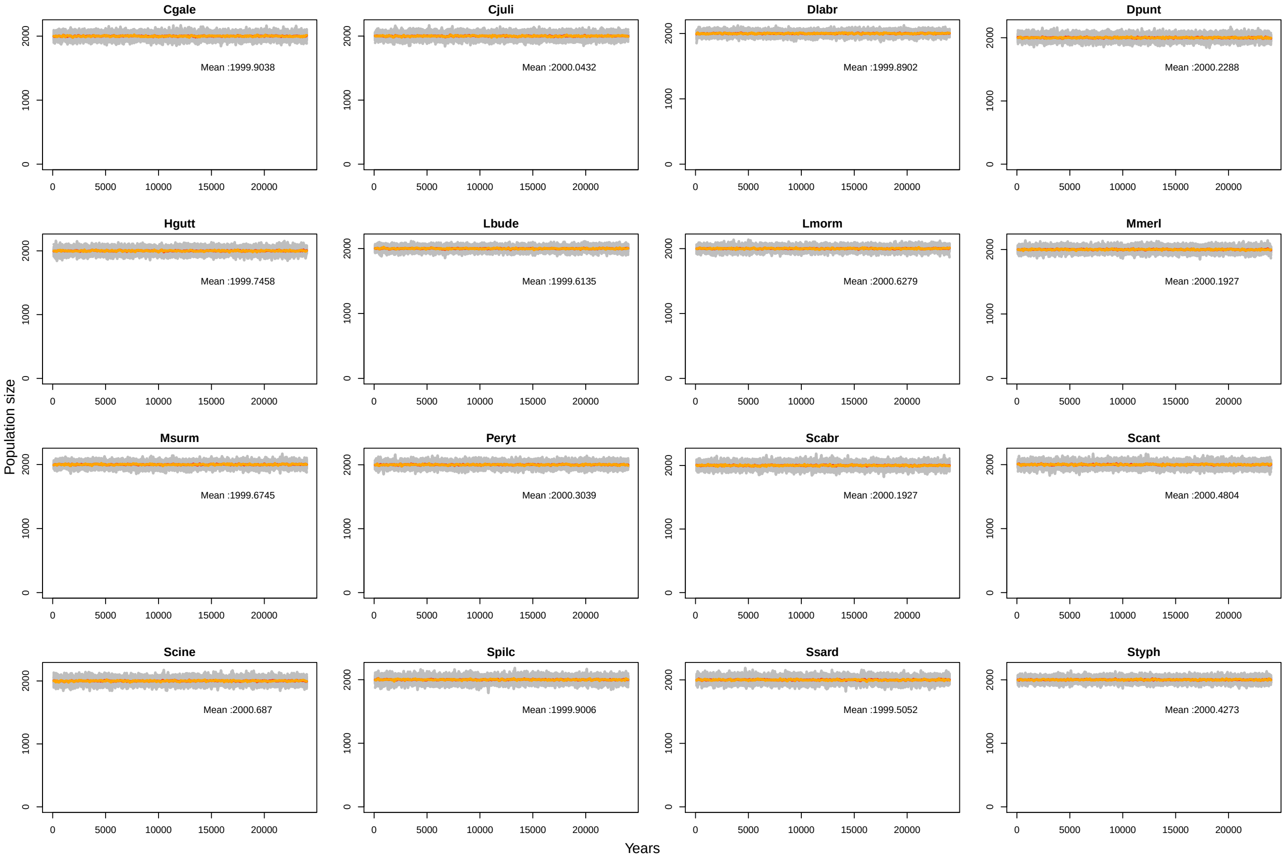

Figure S20: **Population size count for the 50 iterations of the 16 species for set 4 of life tables (age at first maturity at 1 year old, increasing age-specific survival rate, increasing age-specific fecundity and no differences between sex-specific life tables)**. Adult population size was count for each iteration every 100 years during the 25000 years of the simulation. Each grey line represents population size fluctuations for one iteration. For each species, orange and red line, respectively, represents the median and the mean for all the 50 iterations. Mean over all 50 iterations is written on the plot for each species. Cgale = *Coryphoblennius galerita*, Cjuli = *Coris julis*, Dlabr = *Dicentrarchus labrax*, Dpunt = *Diplodus puntazzo*, Hgutt = *Hippocampus guttulatus*, Lbude = *Lophius budegassa*, Lmorm = *Lithognathus mormyrus*, Mmerl = *Merluccius merluccius*, Msurm = *Mullus surmuletus*, Peryt = *Pagellus erythrinus*, Scabr = *Serranus cabrilla*, Scant = *Spondyliosoma cantharus*, Scine = *Symphodus cinereus*, Spilc = *Sardina pilchardus*, Ssard = *Sarda sarda*, Styph = *Syngnathus typhle*

.

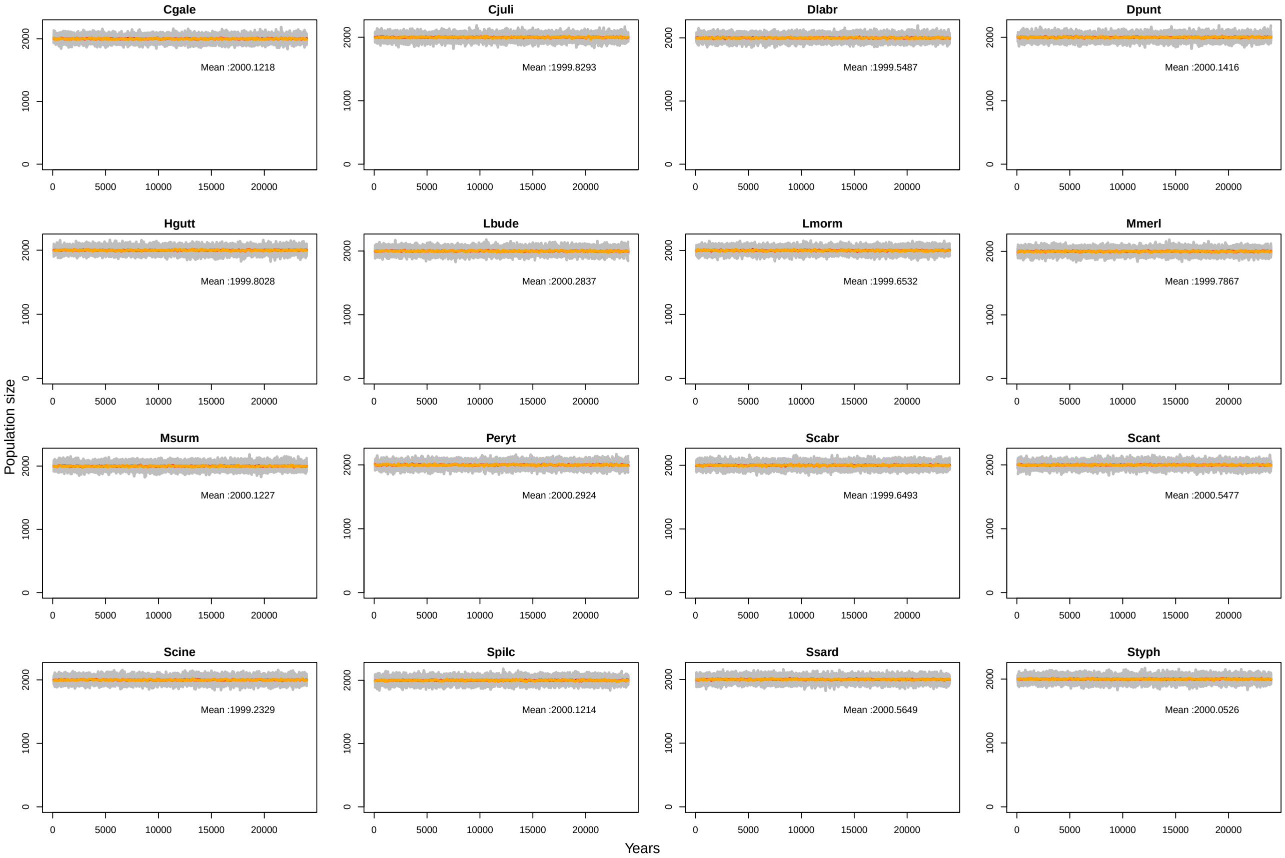

Figure S21: **Population size count for the 50 iterations of the 16 species for set 5 of life tables (age at first maturity at 1 year old, constant age-specific survival rate, constant age-specific fecundity and sex-specific differences in life tables)**. Adult population size was count for each iteration every 100 years during the 25000 years of the simulation. Each grey line represents population size fluctuations for one iteration. For each species, orange and red line, respectively, represents the median and the mean for all the 50 iterations. Mean over all 50 iterations is written on the plot for each species. Cgale = *Coryphoblennius galerita*, Cjuli = *Coris julis*, Dlabr = *Dicentrarchus labrax*, Dpunt = *Diplodus puntazzo*, Hgutt = *Hippocampus guttulatus*, Lbude = *Lophius budegassa*, Lmorm = *Lithognathus mormyrus*, Mmerl = *Merluccius merluccius*, Msurm = *Mullus surmuletus*, Peryt = *Pagellus erythrinus*, Scabr = *Serranus cabrilla*, Scant = *Spondyliosoma cantharus*, Scine = *Symphodus cinereus*, Spilc = *Sardina pilchardus*, Ssard = *Sarda sarda*, Styph = *Syngnathus typhle*

.

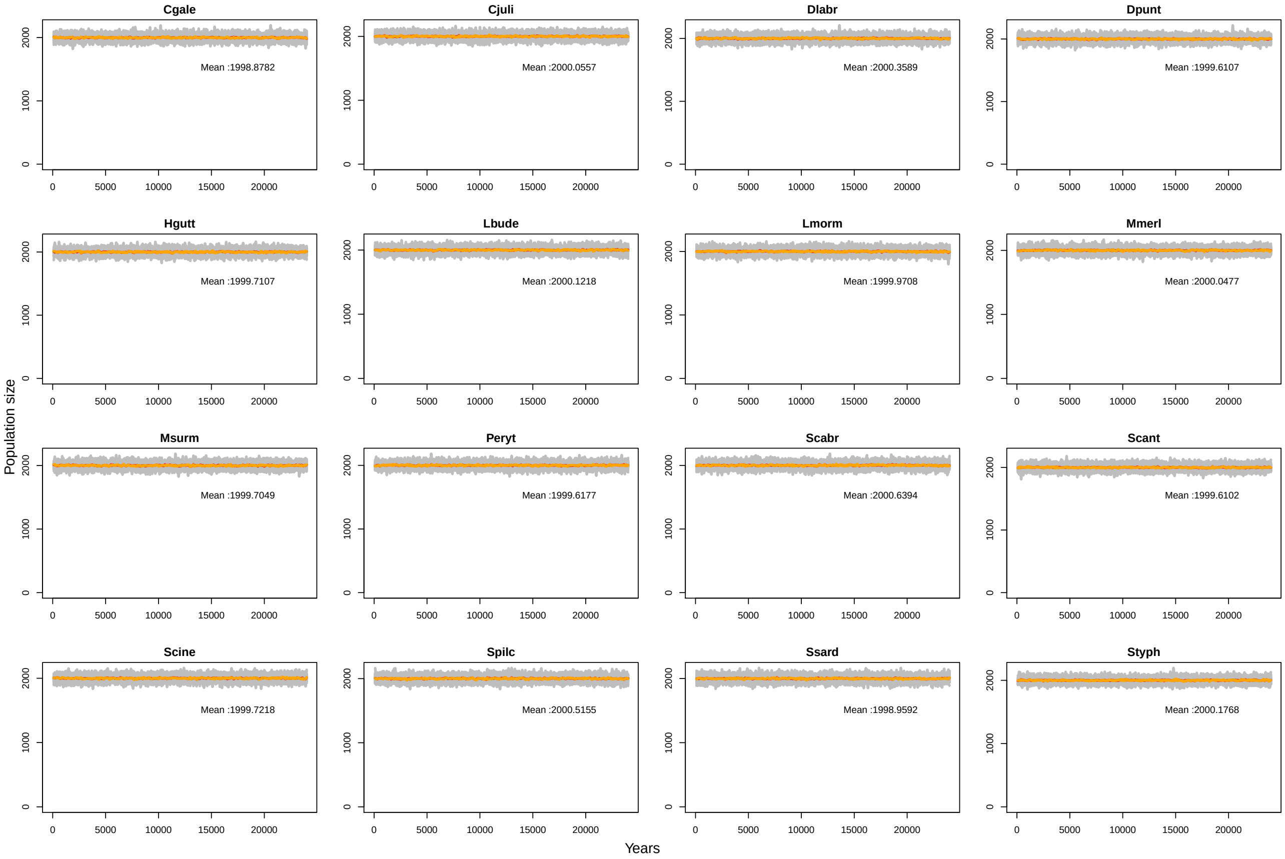

Figure S22: **Population size count for the 50 iterations of the 16 species for set 6 of life tables (age at first maturity at 1 year old, increasing age-specific survival rate, constant age-specific fecundity and sex-specific differences in life tables)**. Adult population size was count for each iteration every 100 years during the 25000 years of the simulation. Each grey line represents population size fluctuations for one iteration. For each species, orange and red line, respectively, represents the median and the mean for all the 50 iterations. Mean over all 50 iterations is written on the plot for each species. Cgale = *Coryphoblennius galerita*, Cjuli = *Coris julis*, Dlabr = *Dicentrarchus labrax*, Dpunt = *Diplodus puntazzo*, Hgutt = *Hippocampus guttulatus*, Lbude = *Lophius budegassa*, Lmorm = *Lithognathus mormyrus*, Mmerl = *Merluccius merluccius*, Msurm = *Mullus surmuletus*, Peryt = *Pagellus erythrinus*, Scabr = *Serranus cabrilla*, Scant = *Spondyliosoma cantharus*, Scine = *Symphodus cinereus*, Spilc = *Sardina pilchardus*, Ssard = *Sarda sarda*, Styph = *Syngnathus typhle*

.

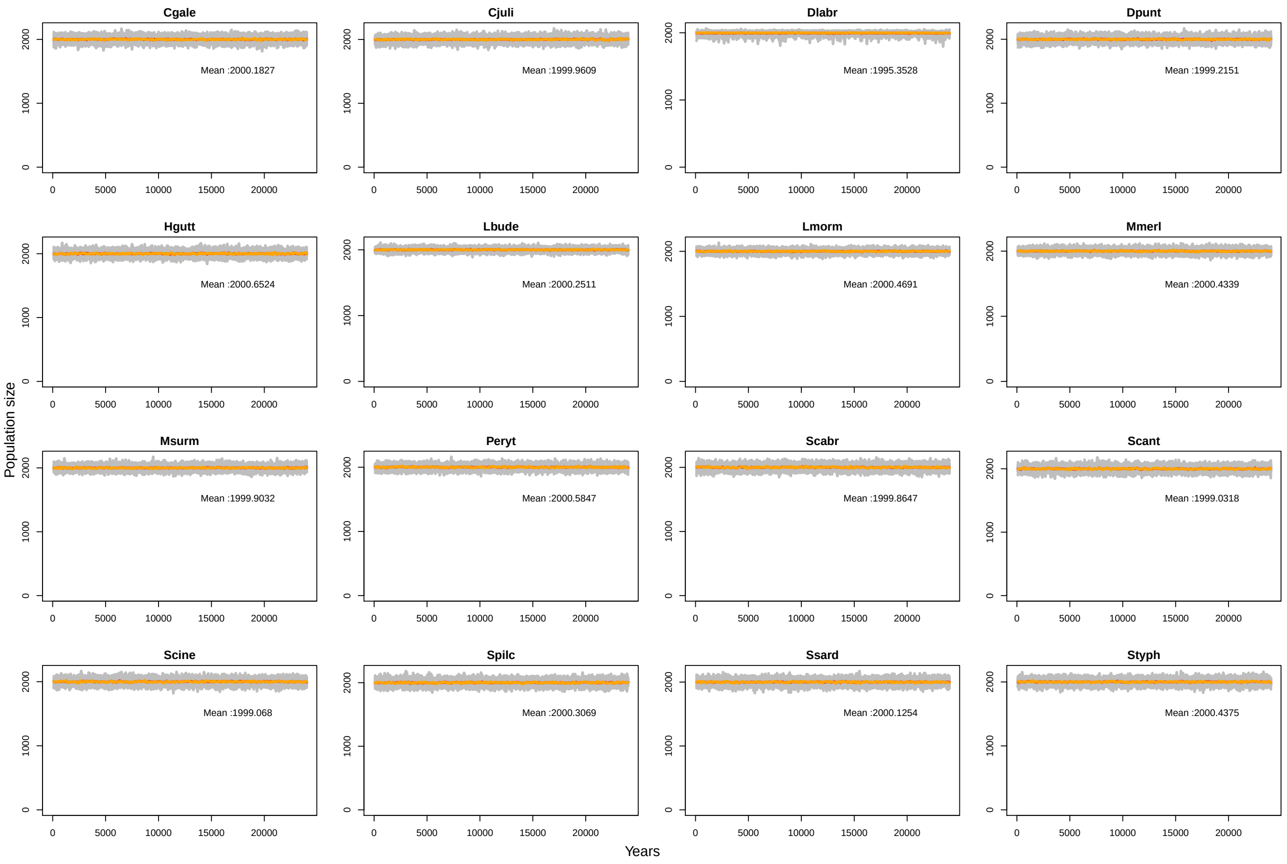

Figure S23: **Population size count for the 50 iterations of the 16 species for set 7 of life tables (age at first maturity at 1 year old, constant age-specific survival rate, increasing age-specific fecundity and sex-specific differences in life tables)**. Adult population size was count for each iteration every 100 years during the 25000 years of the simulation. Each grey line represents population size fluctuations for one iteration. For each species, orange and red line, respectively, represents the median and the mean for all the 50 iterations. Mean over all 50 iterations is written on the plot for each species. Cgale = *Coryphoblennius galerita*, Cjuli = *Coris julis*, Dlabr = *Dicentrarchus labrax*, Dpunt = *Diplodus puntazzo*, Hgutt = *Hippocampus guttulatus*, Lbude = *Lophius budegassa*, Lmorm = *Lithognathus mormyrus*, Mmerl = *Merluccius merluccius*, Msurm = *Mullus surmuletus*, Peryt = *Pagellus erythrinus*, Scabr = *Serranus cabrilla*, Scant = *Spondyliosoma cantharus*, Scine = *Symphodus cinereus*, Spilc = *Sardina pilchardus*, Ssard = *Sarda sarda*, Styph = *Syngnathus typhle*

.

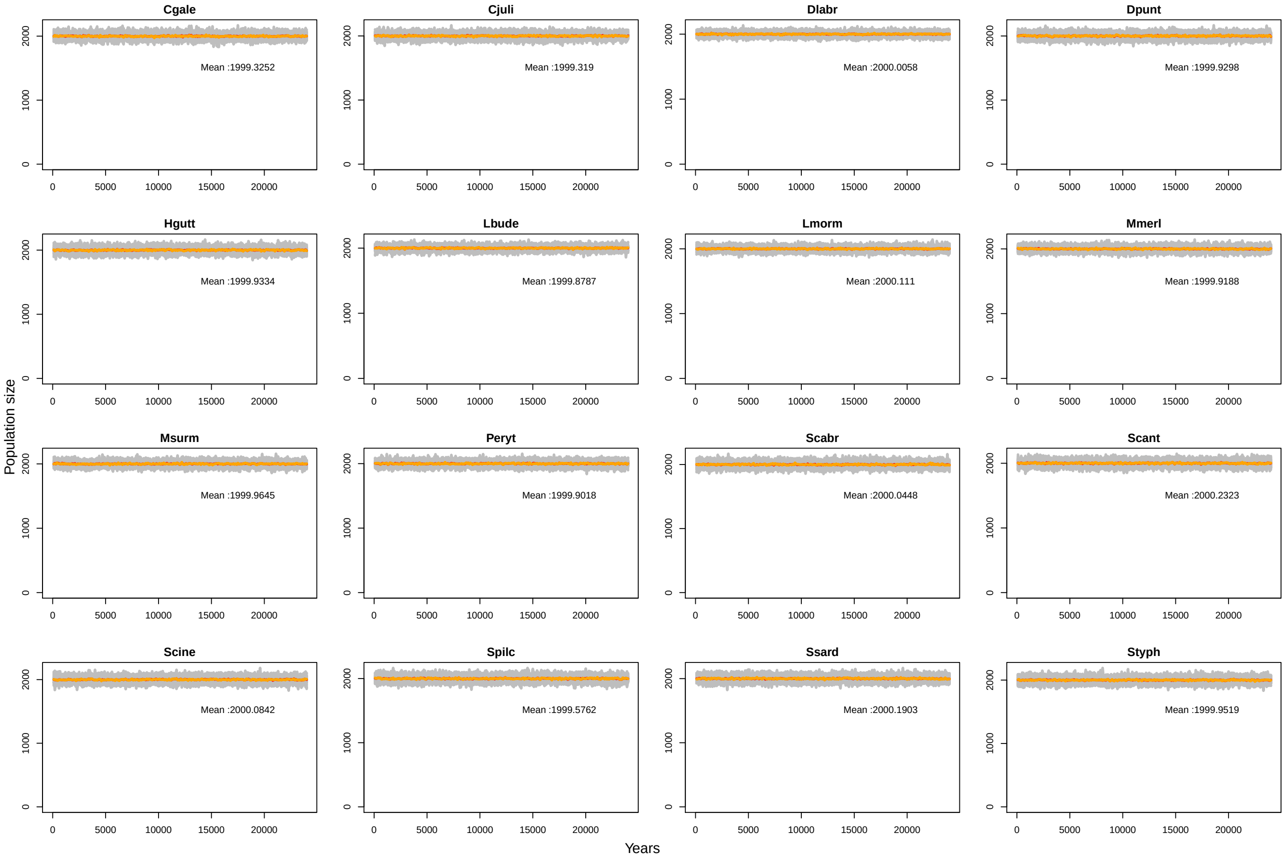

Figure S24: **Population size count for the 50 iterations of the 16 species for set 8 of life tables (age at first maturity at 1 year old, increasing age-specific survival rate, increasing age-specific fecundity and sex-specific differences in life tables)**. Adult population size was count for each iteration every 100 years during the 25000 years of the simulation. Each grey line represents population size fluctuations for one iteration. For each species, orange and red line, respectively, represents the median and the mean for all the 50 iterations. Mean over all 50 iterations is written on the plot for each species. Cgale = *Coryphoblennius galerita*, Cjuli = *Coris julis*, Dlabr = *Dicentrarchus labrax*, Dpunt = *Diplodus puntazzo*, Hgutt = *Hippocampus guttulatus*, Lbude = *Lophius budegassa*, Lmorm = *Lithognathus mormyrus*, Mmerl = *Merluccius merluccius*, Msurm = *Mullus surmuletus*, Peryt = *Pagellus erythrinus*, Scabr = *Serranus cabrilla*, Scant = *Spondyliosoma cantharus*, Scine = *Symphodus cinereus*, Spilc = *Sardina pilchardus*, Ssard = *Sarda sarda*, Styph = *Syngnathus typhle*

.

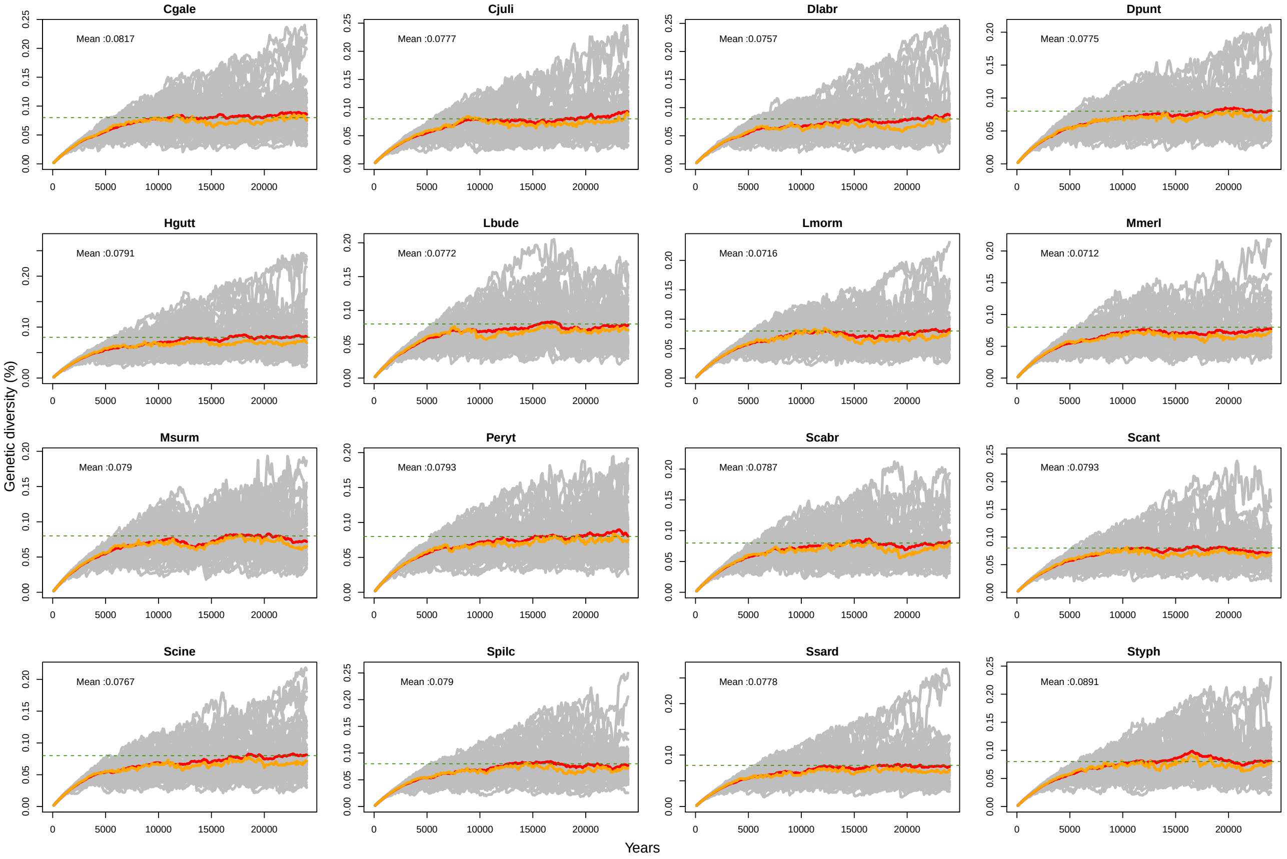

Figure S25: **Genetic diversity simulated for each of the 16 species for set 1 of life tables (age at first maturity at 1 year old, constant age-specific survival rate, constant age-specific fecundity and no differences between sex-specific life tables)**. Simulation were conducted with `SLiM` v.3.3.1 for 25000 years, with mutation rate, $\mu = 1e^{-7}$ on a 1 Mb non recombining loci, and carrying capacity equals $N = 2000$. Genetic diversity was estimated for each iteration every 100 years during the 25000 years of the simulation. Each grey line represents genetic diversity fluctuation for one iteration. For each species, orange and red line, respectively, represents the median and the mean for all the 50 iterations. Green dashed line represents the genetic diversity expected at mutation-drift equilibruim in a Wright-Fisher model ($4N\mu = 4 \times 2000 \times 1e^{-7} = 0.0008 = 0.08\%$). Mean over all 50 iterations, calculated with genetic diversity values estimated between 15 000 and 25 000 years after the beginning of the simulation, is written on the plot for each species. Cgale = *Coryphoblennius galerita*, Cjuli = *Coris julis*, Dlabr = *Dicentrarchus labrax*, Dpunt = *Diplodus puntazzo*, Hgutt = *Hippocampus guttulatus*, Lbude = *Lophius budegassa*, Lmorm = *Lithognathus mormyrus*, Mmerl = *Merluccius merluccius*, Msurm = *Mullus surmuletus*, Peryt = *Pagellus erythrinus*, Scabr = *Serranus cabrilla*, Scant = *Spondyliosoma cantharus*, Scine = *Symphodus cinereus*, Spilc = *Sardina pilchardus*, Ssard = *Sarda sarda*, Styph = *Syngnathus typhle*

.

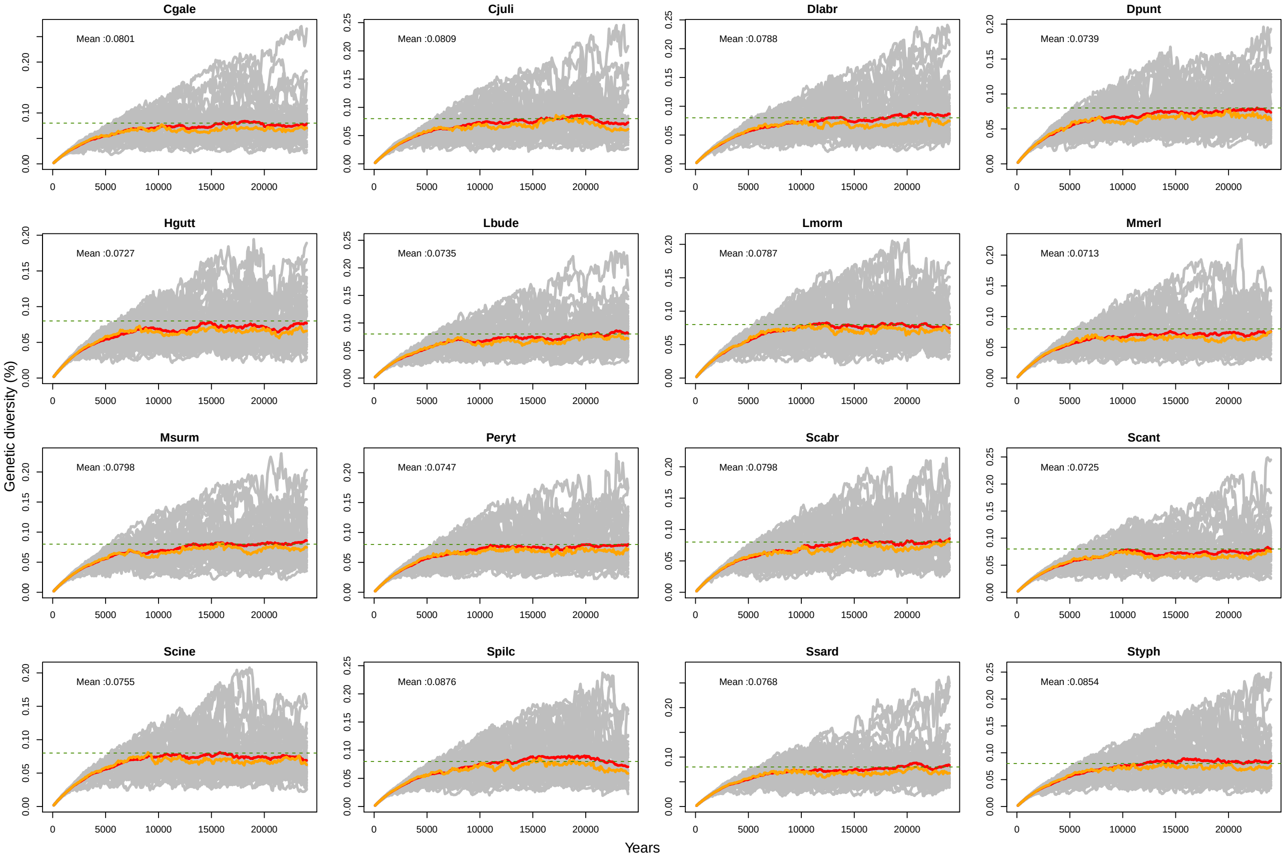

Figure S26: **Genetic diversity simulated for each of the 16 species for set 2 of life tables (age at first maturity at 1 year old, increasing age-specific survival rate, constant age-specific fecundity and no differences between sex-specific life tables)**. Simulation were conducted with `SLiM` v.3.3.1 for 25000 years, with mutation rate, $\mu = 1e^{-7}$ on a 1 Mb non recombining loci, and carrying capacity equals $N = 2000$. Genetic diversity was estimated for each iteration every 100 years during the 25000 years of the simulation. Each grey line represents genetic diversity fluctuation for one iteration. For each species, orange and red line, respectively, represents the median and the mean for all the 50 iterations. Green dashed line represents the genetic diversity expected at mutation-drift equilibruim in a Wright-Fisher model ($4N\mu = 4 \times 2000 \times 1e^{-7} = 0.0008 = 0.08\%$). Mean over all 50 iterations, calculated with genetic diversity values estimated between 15 000 and 25 000 years after the beginning of the simulation, is written on the plot for each species. Cgale = *Coryphoblennius galerita*, Cjuli = *Coris julis*, Dlabr = *Dicentrarchus labrax*, Dpunt = *Diplodus puntazzo*, Hgutt = *Hippocampus guttulatus*, Lbude = *Lophius budegassa*, Lmorm = *Lithognathus mormyrus*, Mmerl = *Merluccius merluccius*, Msurm = *Mullus surmuletus*, Peryt = *Pagellus erythrinus*, Scabr = *Serranus cabrilla*, Scant = *Spondyliosoma cantharus*, Scine = *Symphodus cinereus*, Spilc = *Sardina pilchardus*, Ssard = *Sarda sarda*, Styph = *Syngnathus typhle*

.

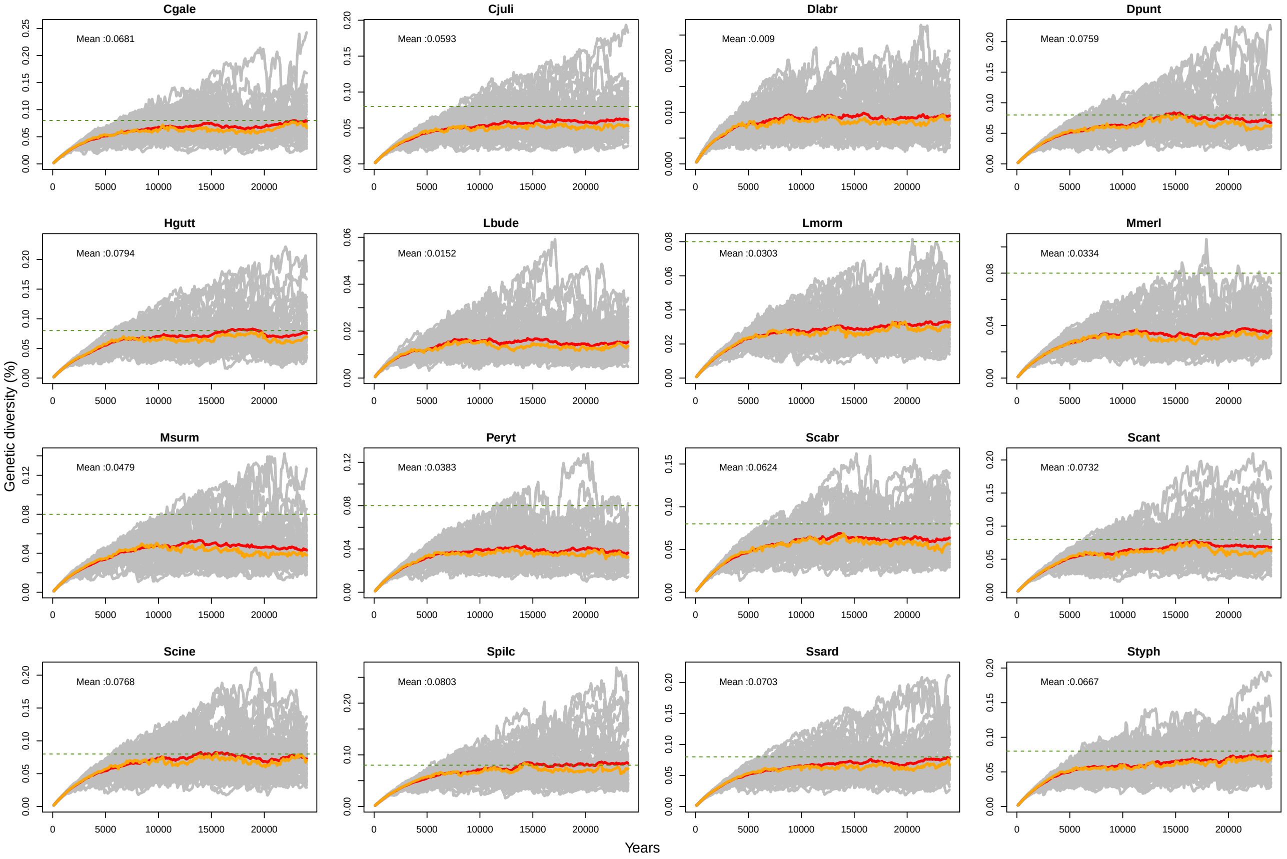

Figure S27: **Genetic diversity simulated for each of the 16 species for set 3 of life tables (age at first maturity at 1 year old, constant age-specific survival rate, increasing age-specific fecundity and no differences between sex-specific life tables)**. Simulation were conducted with `SLiM` v.3.3.1 for 25000 years, with mutation rate, $\mu = 1e^{-7}$ on a 1 Mb non recombining loci, and carrying capacity equals $N = 2000$. Genetic diversity was estimated for each iteration every 100 years during the 25000 years of the simulation. Each grey line represents genetic diversity fluctuation for one iteration. For each species, orange and red line, respectively, represents the median and the mean for all the 50 iterations. Green dashed line represents the genetic diversity expected at mutation-drift equilibruim in a Wright-Fisher model ($4N\mu = 4 \times 2000 \times 1e^{-7} = 0.0008 = 0.08\%$). Mean over all 50 iterations, calculated with genetic diversity values estimated between 15 000 and 25 000 years after the beginning of the simulation, is written on the plot for each species. Cgale = *Coryphoblennius galerita*, Cjuli = *Coris julis*, Dlabr = *Dicentrarchus labrax*, Dpunt = *Diplodus puntazzo*, Hgutt = *Hippocampus guttulatus*, Lbude = *Lophius budegassa*, Lmorm = *Lithognathus mormyrus*, Mmerl = *Merluccius merluccius*, Msurm = *Mullus surmuletus*, Peryt = *Pagellus erythrinus*, Scabr = *Serranus cabrilla*, Scant = *Spondyliosoma cantharus*, Scine = *Symphodus cinereus*, Spilc = *Sardina pilchardus*, Ssard = *Sarda sarda*, Styph = *Syngnathus typhle*

.

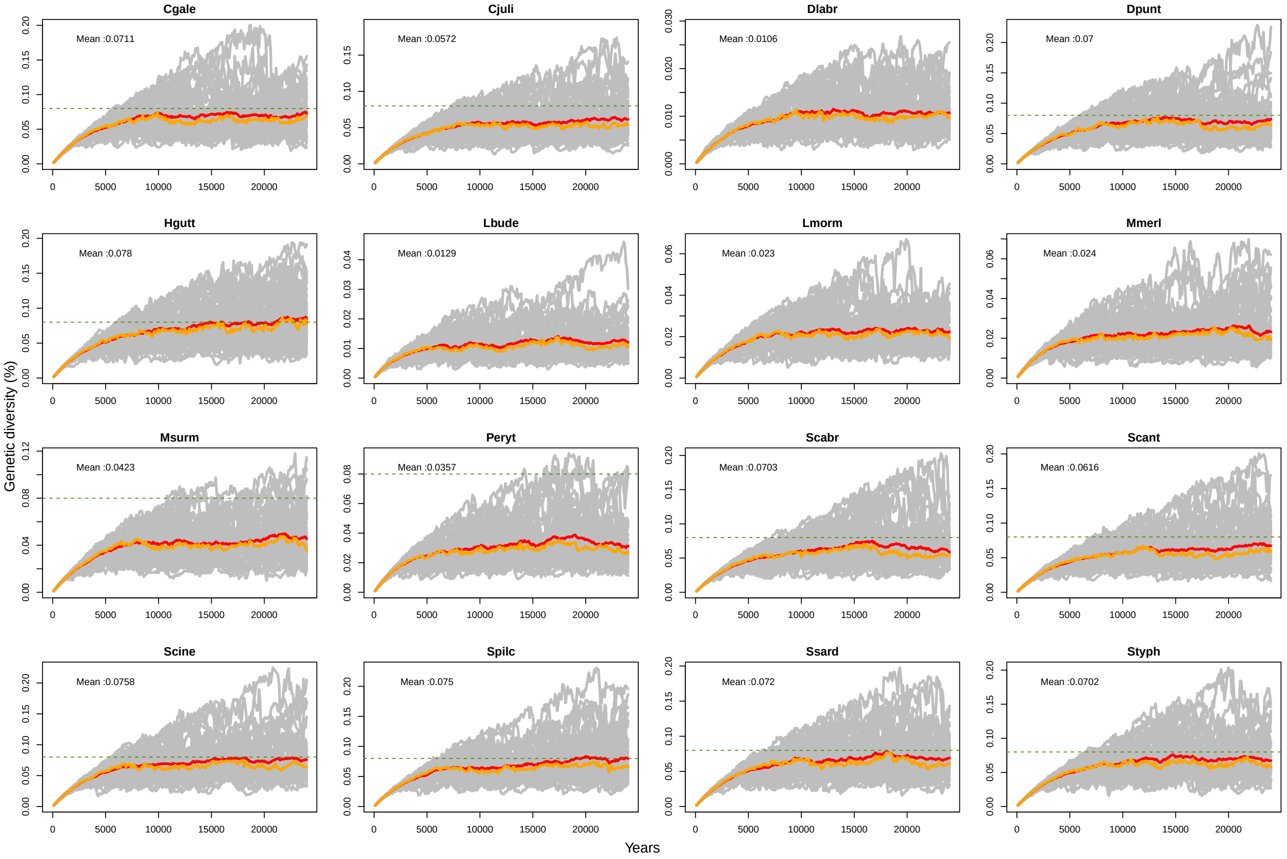

Figure S28: **Genetic diversity simulated for each of the 16 species for set 4 of life tables (age at first maturity at 1 year old, increasing age-specific survival rate, increasing age-specific fecundity and no differences between sex-specific life tables)**. Simulation were conducted with `SLiM` v.3.3.1 for 25000 years, with mutation rate, $\mu = 1e^{-7}$ on a 1 Mb non recombining loci, and carrying capacity equals $N = 2000$. Genetic diversity was estimated for each iteration every 100 years during the 25000 years of the simulation. Each grey line represents genetic diversity fluctuation for one iteration. For each species, orange and red line, respectively, represents the median and the mean for all the 50 iterations. Green dashed line represents the genetic diversity expected at mutation-drift equilibruim in a Wright-Fisher model ($4N\mu = 4 \times 2000 \times 1e^{-7} = 0.0008 = 0.08\%$). Mean over all 50 iterations, calculated with genetic diversity values estimated between 15 000 and 25 000 years after the beginning of the simulation, is written on the plot for each species. Cgale = *Coryphoblennius galerita*, Cjuli = *Coris julis*, Dlabr = *Dicentrarchus labrax*, Dpunt = *Diplodus puntazzo*, Hgutt = *Hippocampus guttulatus*, Lbude = *Lophius budegassa*, Lmorm = *Lithognathus mormyrus*, Mmerl = *Merluccius merluccius*, Msurm = *Mullus surmuletus*, Peryt = *Pagellus erythrinus*, Scabr = *Serranus cabrilla*, Scant = *Spondyliosoma cantharus*, Scine = *Symphodus cinereus*, Spilc = *Sardina pilchardus*, Ssard = *Sarda sarda*, Styph = *Syngnathus typhle*

.

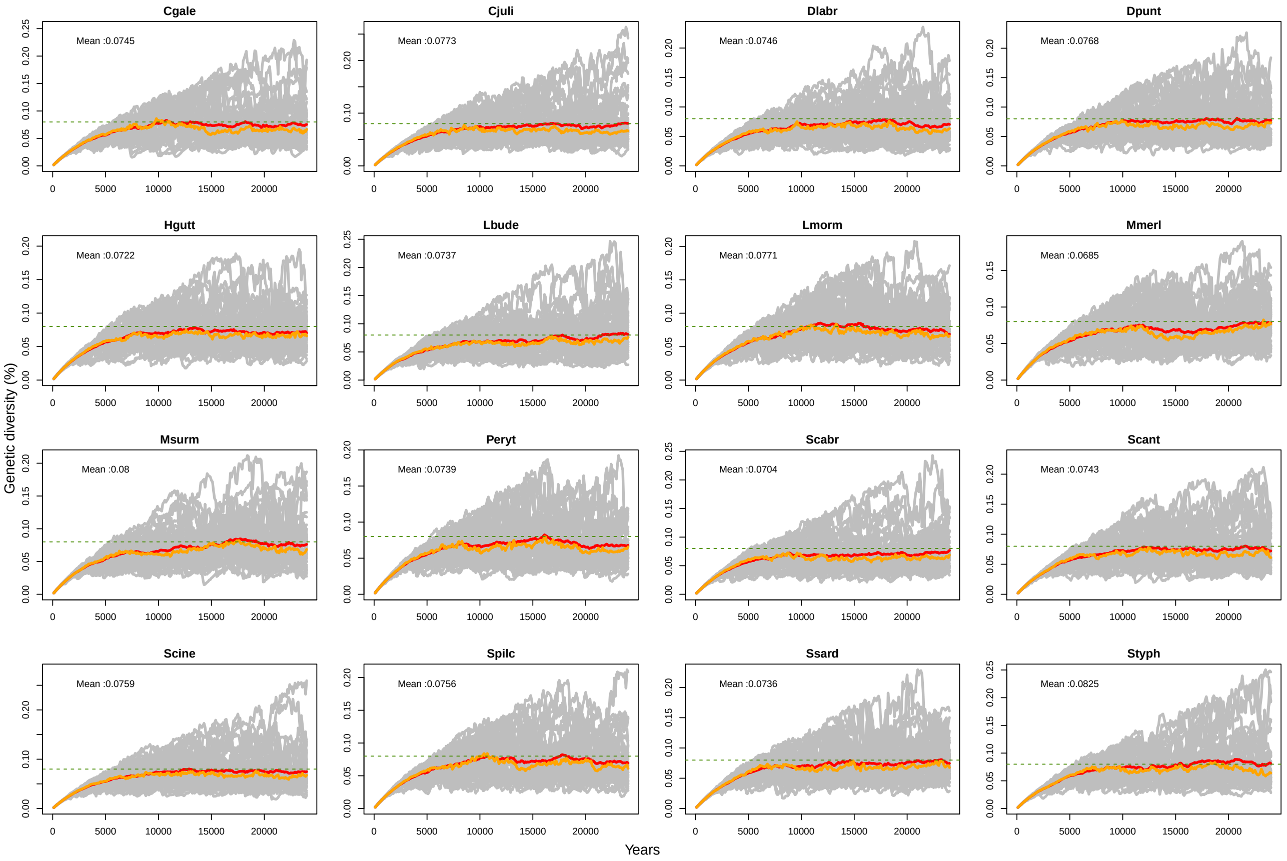

Figure S29: **Genetic diversity simulated for each of the 16 species for set 5 of life tables (age at first maturity at 1 year old, constant age-specific survival rate, constant age-specific fecundity and sex-specific differences in life tables)**. Simulation were conducted with SLiM v.3.3.1 for 25000 years, with mutation rate, $\mu = 1e^{-7}$ on a 1 Mb non recombining loci, and carrying capacity equals $N = 2000$. Genetic diversity was estimated for each iteration every 100 years during the 25000 years of the simulation. Each grey line represents genetic diversity fluctuation for one iteration. For each species, orange and red line, respectively, represents the median and the mean for all the 50 iterations. Green dashed line represents the genetic diversity expected at mutation-drift equilibruim in a Wright-Fisher model ($4N\mu = 4 \times 2000 \times 1e^{-7} = 0.0008 = 0.08\%$). Mean over all 50 iterations, calculated with genetic diversity values estimated between 15 000 and 25 000 years after the beginning of the simulation, is written on the plot for each species. Cgale = *Coryphoblennius galerita*, Cjuli = *Coris julis*, Dlabr = *Dicentrarchus labrax*, Dpunt = *Diplodus puntazzo*, Hgutt = *Hippocampus guttulatus*, Lbude = *Lophius budegassa*, Lmorm = *Lithognathus mormyrus*, Mmerl = *Merluccius merluccius*, Msurm = *Mullus surmuletus*, Peryt = *Pagellus erythrinus*, Scabr = *Serranus cabrilla*, Scant = *Spondyliosoma cantharus*, Scine = *Symphodus cinereus*, Spilc = *Sardina pilchardus*, Ssard = *Sarda sarda*, Styph = *Syngnathus typhle*

.

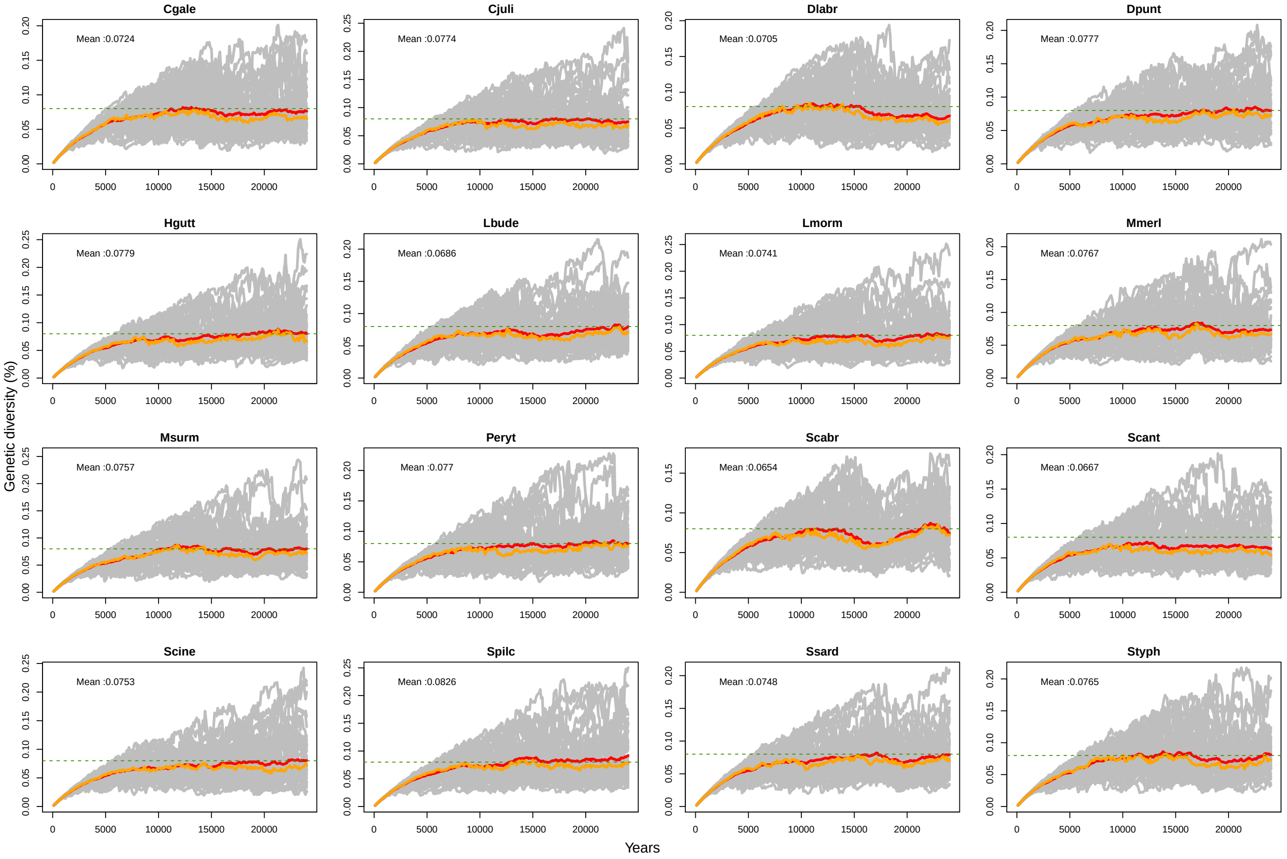

Figure S30: **Genetic diversity simulated for each of the 16 species for set 6 of life tables (age at first maturity at 1 year old, increasing age-specific survival rate, constant age-specific fecundity and sex-specific differences in life tables)**. Simulation were conducted with `SLiM` v.3.3.1 for 25000 years, with mutation rate, $\mu = 1e^{-7}$ on a 1 Mb non recombining loci, and carrying capacity equals $N = 2000$. Genetic diversity was estimated for each iteration every 100 years during the 25000 years of the simulation. Each grey line represents genetic diversity fluctuation for one iteration. For each species, orange and red line, respectively, represents the median and the mean for all the 50 iterations. Green dashed line represents the genetic diversity expected at mutation-drift equilibruim in a Wright-Fisher model ($4N\mu = 4 \times 2000 \times 1e^{-7} = 0.0008 = 0.08\%$). Mean over all 50 iterations, calculated with genetic diversity values estimated between 15 000 and 25 000 years after the beginning of the simulation, is written on the plot for each species. Cgale = *Coryphoblennius galerita*, Cjuli = *Coris julis*, Dlabr = *Dicentrarchus labrax*, Dpunt = *Diplodus puntazzo*, Hgutt = *Hippocampus guttulatus*, Lbude = *Lophius budegassa*, Lmorm = *Lithognathus mormyrus*, Mmerl = *Merluccius merluccius*, Msurm = *Mullus surmuletus*, Peryt = *Pagellus erythrinus*, Scabr = *Serranus cabrilla*, Scant = *Spondyliosoma cantharus*, Scine = *Symphodus cinereus*, Spilc = *Sardina pilchardus*, Ssard = *Sarda sarda*, Styph = *Syngnathus typhle*

.

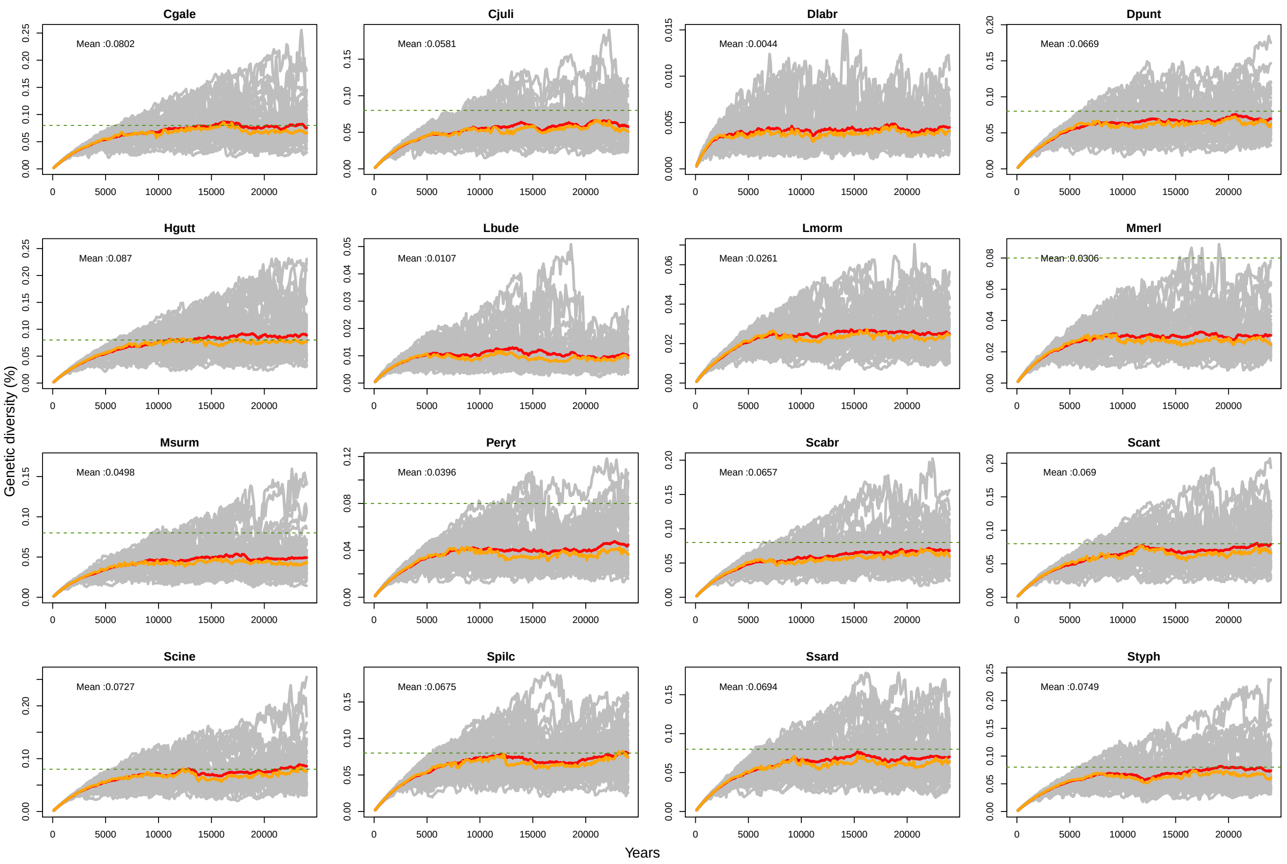

Figure S31: **Genetic diversity simulated for each of the 16 species for set 7 of life tables (age at first maturity at 1 year old, constant age-specific survival rate, increasing age-specific fecundity and sex-specific differences in life tables)**. Simulation were conducted with `SLiM` v.3.3.1 for 25000 years, with mutation rate, $\mu = 1e^{-7}$ on a 1 Mb non recombining loci, and carrying capacity equals $N = 2000$. Genetic diversity was estimated for each iteration every 100 years during the 25000 years of the simulation. Each grey line represents genetic diversity fluctuation for one iteration. For each species, orange and red line, respectively, represents the median and the mean for all the 50 iterations. Green dashed line represents the genetic diversity expected at mutation-drift equilibruim in a Wright-Fisher model ($4N\mu = 4 \times 2000 \times 1e^{-7} = 0.0008 = 0.08\%$). Mean over all 50 iterations, calculated with genetic diversity values estimated between 15 000 and 25 000 years after the beginning of the simulation, is written on the plot for each species. Cgale = *Coryphoblennius galerita*, Cjuli = *Coris julis*, Dlabr = *Dicentrarchus labrax*, Dpunt = *Diplodus puntazzo*, Hgutt = *Hippocampus guttulatus*, Lbude = *Lophius budegassa*, Lmorm = *Lithognathus mormyrus*, Mmerl = *Merluccius merluccius*, Msurm = *Mullus surmuletus*, Peryt = *Pagellus erythrinus*, Scabr = *Serranus cabrilla*, Scant = *Spondyliosoma cantharus*, Scine = *Symphodus cinereus*, Spilc = *Sardina pilchardus*, Ssard = *Sarda sarda*, Styph = *Syngnathus typhle*

.

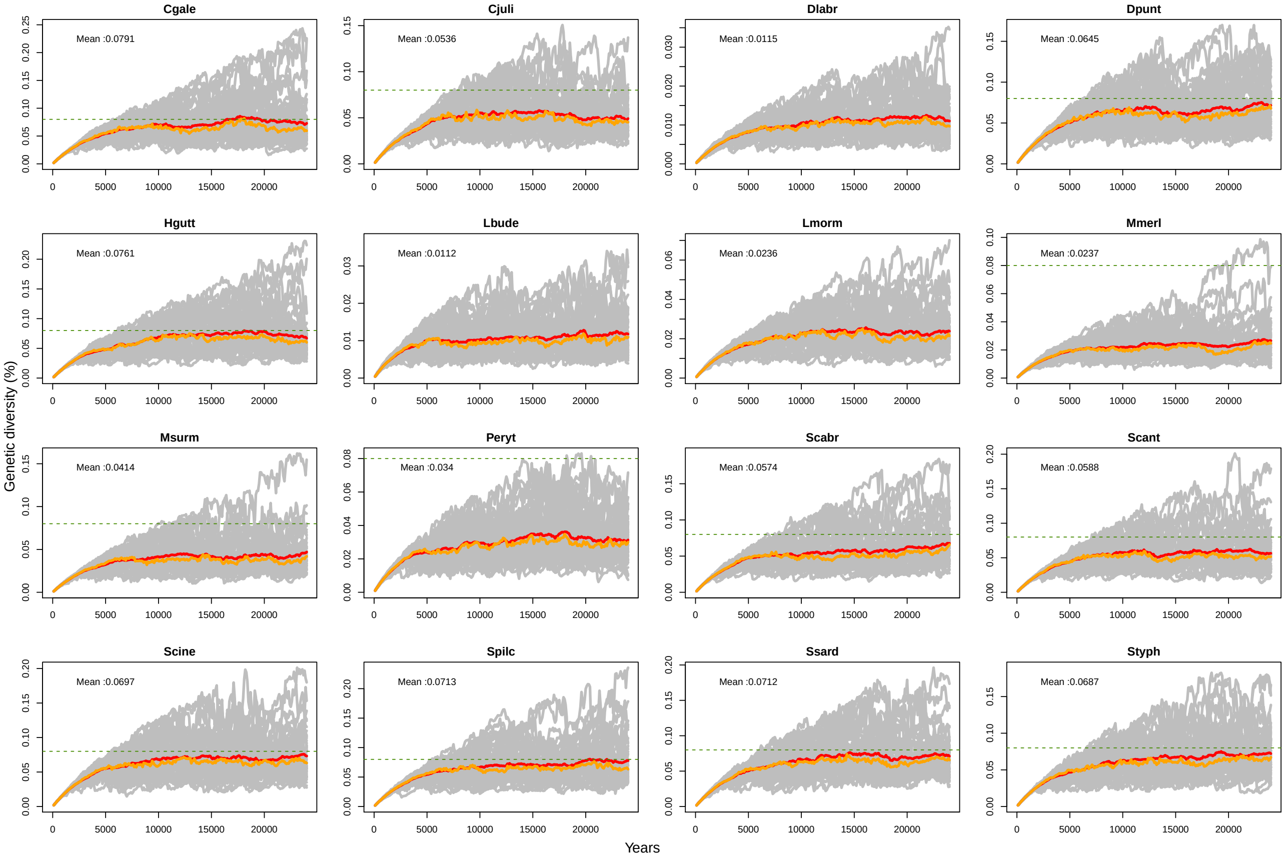

Figure S32: **Genetic diversity simulated for each of the 16 species for set 8 of life tables (age at first maturity at 1 year old, increasing age-specific survival rate, increasing age-specific fecundity and sex-specific differences in life tables)**. Simulation were conducted with `SLiM` v.3.3.1 for 25000 years, with mutation rate, $\mu = 1e^{-7}$ on a 1 Mb non recombining loci, and carrying capacity equals $N = 2000$. Genetic diversity was estimated for each iteration every 100 years during the 25000 years of the simulation. Each grey line represents genetic diversity fluctuation for one iteration. For each species, orange and red line, respectively, represents the median and the mean for all the 50 iterations. Green dashed line represents the genetic diversity expected at mutation-drift equilibruim in a Wright-Fisher model ($4N\mu = 4 \times 2000 \times 1e^{-7} = 0.0008 = 0.08\%$). Mean over all 50 iterations, calculated with genetic diversity values estimated between 15 000 and 25 000 years after the beginning of the simulation, is written on the plot for each species. Cgale = *Coryphoblennius galerita*, Cjuli = *Coris julis*, Dlabr = *Dicentrarchus labrax*, Dpunt = *Diplodus puntazzo*, Hgutt = *Hippocampus guttulatus*, Lbude = *Lophius budegassa*, Lmorm = *Lithognathus mormyrus*, Mmerl = *Merluccius merluccius*, Msurm = *Mullus surmuletus*, Peryt = *Pagellus erythrinus*, Scabr = *Serranus cabrilla*, Scant = *Spondyliosoma cantharus*, Scine = *Symphodus cinereus*, Spilc = *Sardina pilchardus*, Ssard = *Sarda sarda*, Styph = *Syngnathus typhle*

.
