# Supplementary material for: Age‐specific survivorship and fecundity shape genetic diversity in marine fishes
Source: Evol Lett. 2021 Dec 24;6(1):46–62. doi: 10.1002/evl3.265 (PMC8802244; doi:10.1002/evl3.265)
Supplement: Supplementary file 3 — Data S2 [file EVL3-6-46-s001.pdf]

Appendix 1 to Barry and al. "Life tables shape genetic diversity in marine fishes": life tables of the 16 marine teleostean species

Pierre Barry, Thomas Broquet, Pierre-Alexandre Gagnaire

Each page represents the informations on life tables and corresponding bibliographic references for each of the 16 species. On top, the image from Iglésias[13], reproduced with permissions, vernacular and Latin name of the species.

The first table, on top, shows length at infinity L_{inf}, growth parameter K, and t_0 from Von Bertalanffy equation modeling age-length relationship. Maturity show age at first maturity, and maximum age, lifespan. Values are shown for males, females and for combined sexes. On bottom of the first table, $F = y(L)$ shows the corresponding model between age and fecundity, either linear ($F = \alpha + \beta \times L$), exponential ($F = \alpha \times exp[\beta \times L]$) or power-law ($F = \alpha \times L^{\beta}$). α and β show the corresponding parameter of the relationship between age and fecundity. Corresponding bibliographic references are indicated in the last column.

The second table shows life tables components calculated with the parameters of the first table: for each age, L_x indicates length in centimeters for combined sexes, $L_{x,f}$, length for females only and $L_{x,m}$, length for males only. l_x, $l_{x,f}$ and $l_{x,m}$ indicates age-specific survival for combined sexes, females and males, respectively. Cum_c, Cum_f and Cum_m indicates cumulative age-specific survival for combined sexes, females and males respectively. B_x, $B_{x,f}$ and $B_{x,m}$ show relative fecundity for combined sexes, females and males, respectively (max fecundity equals 1 at lifespan).

On the bottom, solid lines represent the age-specific probability of survival until age x curves (y left-axis), dashed lines represent the age-specific fecundity curves (y right-axis). Black, blue and red lines represent combined sexes, male and females curves, respectively.

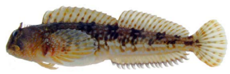

Montagu's blenny

Coryphoblennius galerita

Parameter	Combined	Female	Male	Ref
L_{inf}	6.54	6.54	6.54	Milton[16]
K	0.432	0.432	0.432	
t_0	-1.247	-1.247	-1.247	
Maturity	1	1	1	
Max age	6	6	6	
$F = y(L)$	Linear			Carrassón and Bau[5]
α	-2146.4			
β	710.07			

Age	L_x	$L_{x,f}$	$L_{x,m}$	l_x	$l_{x,f}$	$l_{x,m}$	Cum_c	Cum_f	Cum_m	B_x	$B_{x,f}$	$B_{x,m}$
1	4.4	4.4	4.4	0.46	0.46	0.46	0.180	0.180	0.180	0.420	0.420	0.420
2	4.8	4.8	4.8	0.50	0.50	0.50	0.083	0.083	0.083	0.542	0.542	0.542
3	5.3	5.3	5.3	0.55	0.55	0.55	0.041	0.041	0.041	0.695	0.695	0.695
4	5.8	5.8	5.8	0.60	0.60	0.60	0.023	0.023	0.023	0.847	0.847	0.847
5	6.2	6.2	6.2	0.63	0.63	0.63	0.013	0.013	0.013	0.969	0.969	0.969
6	6.3	6.3	6.3	0.00	0.00	0.00	0.008	0.008	0.008	1.00	1.00	1.00

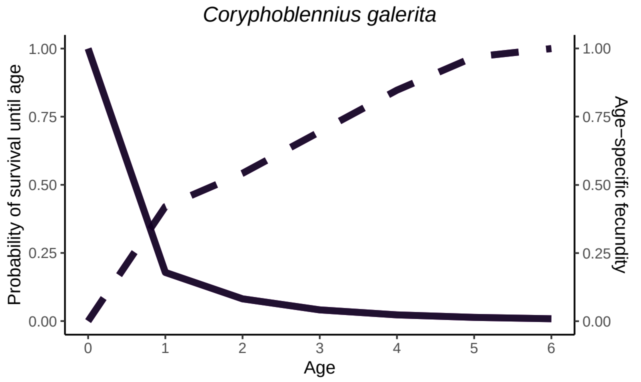

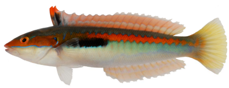

Rainbow wrasse

Coris julis

Parameter	Combined	Female	Male	Ref
L_{inf}	25.4	21.27	29.66	Škeljo[25]
K	0.16	0.21	0.12	
t_0	-1.19	-1.08	-1.52	
Maturity	1	1	1	
Max age	7	5	6	
$F = y(L)$	Power			Škeljo[25]
α	0.902			
β	3.643			

Age	L_x	$L_{x,f}$	$L_{x,m}$	l_x	$l_{x,f}$	$l_{x,m}$	Cum_c	Cum_f	Cum_m	B_x	$B_{x,f}$	$B_{x,m}$
1	8.5	8.7	8.5	0.44	0.45	0.46	0.282	0.305	0.327	0.037	0.119	0.037
2	11.4	11.3	11.5	0.59	0.58	0.61	0.123	0.137	0.150	0.108	0.309	0.112
3	12.9	12.6	13.1	0.64	0.63	0.66	0.073	0.079	0.091	0.169	0.459	0.179
4	14.8	14.9	14.8	0.70	0.70	0.71	0.047	0.050	0.060	0.280	0.846	0.280
5	16.5	15.6	16.6	0.74	0.72	0.75	0.033	0.035	0.043	0.415	1.000	0.425
6	18.1	–	18.1	0.77	–	0.78	0.024	0.025	0.032	0.582	–	0.582
7	21	–	21	0.81	–	0.82	0.018	–	0.025	1.000	–	1.000

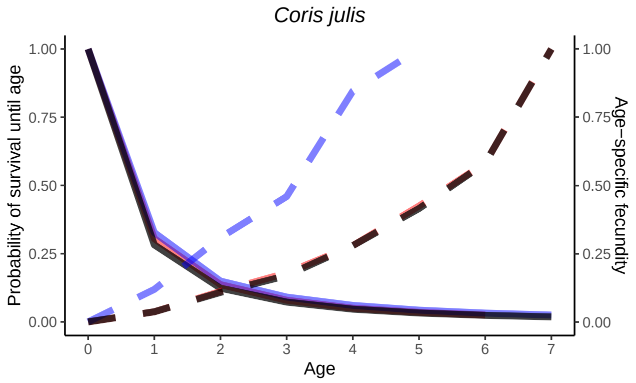

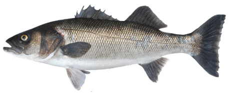

European sea bass

Dicentrarchus labrax

Parameter	Combined	Female	Male	Ref
L_{inf}	83.2	87.8	78.1	Wassef and Emary[28]
K	0.066	0.061	0.075	
t_0	-1.745	-1.797	-1.765	
Maturity	3	2	4	
Max age	15	15	9	
$F = y(L)$	Power			Kara[14]
α	0.00087			
β	5			

Age	L_x	$L_{x,f}$	$L_{x,m}$	l_x	$l_{x,f}$	$l_{x,m}$	Cum_c	Cum_f	Cum_m	B_x	$B_{x,f}$	$B_{x,m}$
1	13.3	13.3	13.6	0.36	0.36	0.36	0.376	0.162	0.179	0.000	0.000	0.000
2	18.1	18.1	18.2	0.52	0.52	0.51	0.134	0.057	0.064	0.000	0.000	0.000
3	21.8	21.8	21.7	0.61	0.61	0.60	0.070	0.030	0.033	0.000	0.000	0.028
4	25.5	25.5	25.4	0.68	0.68	0.67	0.043	0.018	0.020	0.008	0.000	0.062
5	29.5	29.5	29.4	0.73	0.73	0.72	0.029	0.012	0.013	0.017	0.017	0.129
6	33.2	33.2	33	0.77	0.77	0.76	0.021	0.009	0.009	0.031	0.031	0.229
7	36.5	36.5	36.2	0.80	0.80	0.79	0.016	0.007	0.007	0.051	0.051	0.364
8	40.3	40.3	40.3	0.82	0.82	0.82	0.013	0.006	0.006	0.083	0.083	0.623
9	44.3	44.3	44.3	0.84	0.84	0.84	0.011	0.005	0.005	0.133	0.133	1.000
10	48.3	48.3	–	0.86	0.86	–	0.009	0.004	0.004	0.205	0.205	–
11	52.4	52.4	–	0.88	0.88	–	0.008	0.003	–	0.308	0.308	–
12	56.3	56.3	–	0.89	0.89	–	0.007	0.003	–	0.442	0.442	–
13	59.8	59.8	–	0.90	0.90	–	0.006	0.003	–	0.597	0.597	–
14	63.4	63.4	–	0.91	0.91	–	0.005	0.002	–	0.800	0.800	–
15	66.3	66.3	–	0.91	0.91	–	0.005	0.002	–	1.000	1.000	–

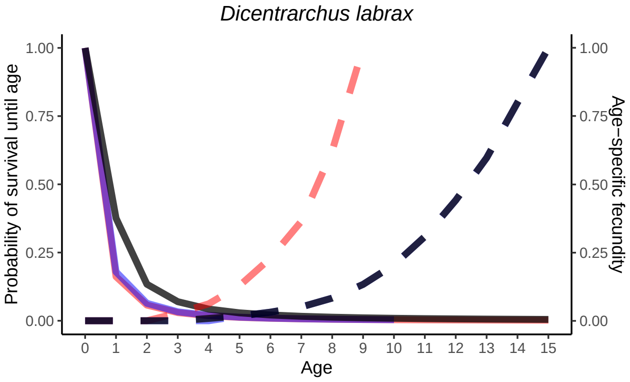

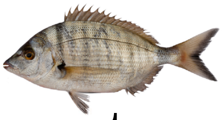

Sharp-snout seabream

Diplodus puntazzo

Parameter	Combined	Female	Male	Ref
L_{inf}	54.1	52.3	52.7	Domínguez-Seoane et al.[8]
K	0.182	0.203	0.187	
t_0	-2.531	-2.225	-2.761	
Maturity	2	2	2	
Max age	10	10	10	
$F = y(L)$	Power			Taieb et al.[26]
α	40.269			
β	2.0483			

Age	L_x	$L_{x,f}$	$L_{x,m}$	l_x	$l_{x,f}$	$l_{x,m}$	Cum_c	Cum_f	Cum_m	B_x	$B_{x,f}$	$B_{x,m}$
1	25.3	25.3	25.3	0.57	0.55	0.57	0.359	0.338	0.364	0.000	0.000	0.000
2	31.0	31.0	31.0	0.66	0.64	0.66	0.203	0.185	0.207	0.000	0.000	0.000
3	34.6	34.6	34.6	0.70	0.69	0.70	0.134	0.118	0.137	0.454	0.454	0.454
4	37.5	37.5	37.5	0.73	0.72	0.73	0.094	0.081	0.096	0.535	0.535	0.535
5	39.1	39.1	39.1	0.74	0.73	0.75	0.068	0.058	0.071	0.583	0.583	0.583
6	43.5	43.5	43.5	0.78	0.77	0.78	0.051	0.042	0.053	0.725	0.725	0.725
7	45.6	45.6	45.6	0.79	0.78	0.79	0.039	0.032	0.041	0.798	0.798	0.798
8	48.4	48.4	48.4	0.81	0.80	0.81	0.031	0.025	0.033	0.902	0.902	0.902
9	49.8	49.8	49.8	0.81	0.80	0.82	0.025	0.020	0.026	0.956	0.956	0.956
10	50.9	50.9	50.9	0.82	0.81	0.82	0.020	0.016	0.021	1.000	1.000	1.000

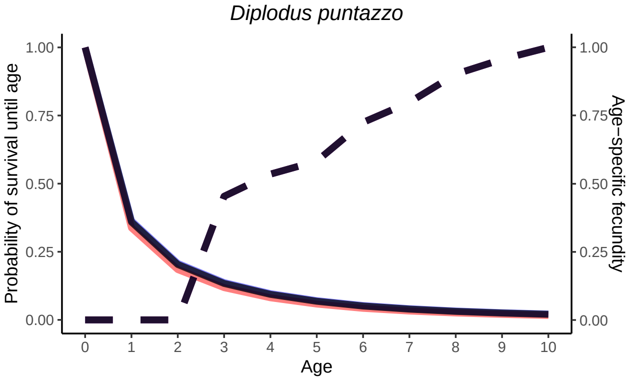

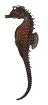

Long-snouted seahorse

Hippocampus guttulatus

Parameter	Combined	Female	Male	Ref
L_{inf}	19.76	19.76	19.76	
K	0.571	0.571	0.571	
t_0	-0.05	-0.083	-0.044	Curtis and Vincent[7]
Maturity	1	1	1	
Max age	5	5	5	
$F = y(L)$	Exponential			
α	78.54			Curtis and Vincent[7]
β	0.16			

Age	L_x	$L_{x,f}$	$L_{x,m}$	l_x	$l_{x,f}$	$l_{x,m}$	Cum_c	Cum_f	Cum_m	B_x	$B_{x,f}$	$B_{x,m}$
1	13.5	13.5	13.5	0.36	0.36	0.36	0.253	0.253	0.253	0.415	0.415	0.415
2	16.5	16.5	16.5	0.47	0.47	0.47	0.092	0.092	0.092	0.670	0.670	0.670
3	18.0	18.0	18.0	0.52	0.52	0.52	0.044	0.044	0.044	0.852	0.852	0.852
4	18.5	18.5	18.5	0.53	0.53	0.53	0.023	0.023	0.023	0.923	0.923	0.923
5	19.0	19.0	19.0	0.55	0.55	0.55	0.012	0.012	0.012	1.000	1.000	1.000

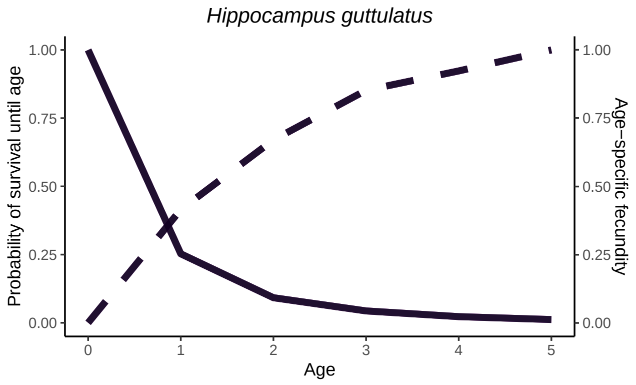

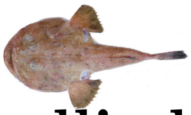

Blackbellied angler

Lophius budegassa

Parameter	Combined	Female	Male	Ref
L_{inf}	102	147.3	102.5	García-Rodríguez et al.[11]
K	0.15	0.091	0.189	
t_0	-0.05	-0.083	-0.044	
Maturity	7	6	8	
Max age	21	21	13	
$F = y(L)$	Linear			Colmenero et al.[6]
α	-694487			
β	16422			

Age	L_x	$L_{x,f}$	$L_{x,m}$	l_x	$l_{x,f}$	$l_{x,m}$	Cum_c	Cum_f	Cum_m	B_x	$B_{x,f}$	$B_{x,m}$
1	21.12	25.44	32.85	0.20	0.28	0.35	1.000	1.000	1.000	0.000	0.000	0.000
2	32.38	36.03	44.84	0.43	0.47	0.52	0.204	0.281	0.353	0.000	0.000	0.000
3	42.08	45.71	54.77	0.57	0.59	0.62	0.088	0.133	0.184	0.000	0.000	0.000
4	50.43	54.55	62.99	0.65	0.67	0.68	0.050	0.078	0.113	0.000	0.000	0.000
5	57.61	62.62	69.79	0.70	0.72	0.71	0.032	0.052	0.076	0.000	0.000	0.000
6	63.79	69.98	75.43	0.74	0.76	0.74	0.023	0.038	0.055	0.000	0.000	0.000
7	69.12	76.71	80.09	0.76	0.78	0.76	0.017	0.029	0.040	0.000	0.000	0.645
8	73.70	82.85	83.95	0.78	0.81	0.77	0.013	0.022	0.031	0.564	0.000	0.711
9	77.64	88.45	87.14	0.80	0.82	0.79	0.010	0.018	0.024	0.635	0.541	0.765
10	81.03	93.57	89.79	0.81	0.84	0.79	0.008	0.015	0.019	0.696	0.601	0.810
11	83.95	98.25	91.98	0.82	0.85	0.80	0.007	0.012	0.015	0.748	0.656	0.848
12	86.47	102.51	93.79	0.83	0.85	0.81	0.005	0.010	0.012	0.793	0.706	0.879
13	88.63	106.41	95.29	0.83	0.86	0.81	0.004	0.009	0.010	0.832	0.752	0.904
14	90.49	109.97	96.53	0.84	0.87	0.81	0.004	0.008	0.008	0.866	0.794	0.925
15	92.10	113.21	97.56	0.84	0.87	0.82	0.003	0.007	0.006	0.895	0.832	0.943
16	93.48	116.18	98.41	0.84	0.88	0.82	0.003	0.006	0.005	0.919	0.867	0.957
17	94.66	118.88	99.11	0.85	0.88	0.82	0.002	0.005	0.004	0.941	0.898	0.969
18	95.68	121.36	99.70	0.85	0.89	0.82	0.002	0.005	0.003	0.959	0.927	0.979
19	96.56	123.61	100.18	0.85	0.89	0.82	0.002	0.004	0.003	0.975	0.954	0.988
20	97.32	125.67	100.58	0.85	0.89	0.82	0.001	0.004	0.002	0.988	0.978	0.994
21	97.97	127.55	100.91	0.85	0.89	0.82	0.001	0.003	0.002	1.000	1.000	1.000

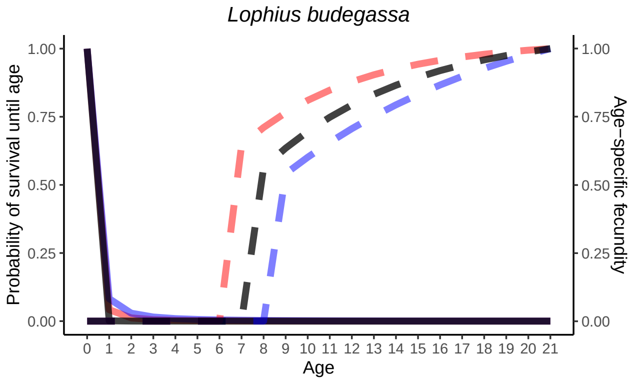

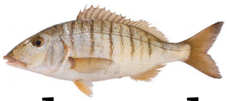

Striped seabream

Lithognathus mormyrus

Parameter	Combined	Female	Male	Ref
L_{inf}	35.3	35.3	35.3	Monteiro et al.[17]
K	0.264	0.264	0.264	
t_0	-0.809	-0.809	-0.809	
Maturity	2	2	2	
Max age	12	12	9	
$F = y(L)$	Power			Faraj et al.[9]
α	0.0026			
β	4.94			

Age	L_x	$L_{x,f}$	$L_{x,m}$	l_x	$l_{x,f}$	$l_{x,m}$	Cum_c	Cum_f	Cum_m	B_x	$B_{x,f}$	$B_{x,m}$
1	13.3	16.3	15.5	0.32	0.43	0.40	1.000	1.000	1.000	0.000	0.000	0.000
2	19.1	19.3	19.3	0.52	0.52	0.52	0.319	0.431	0.404	0.000	0.000	0.000
3	23.0	23.2	22.9	0.61	0.61	0.60	0.165	0.224	0.210	0.051	0.053	0.148
4	24.9	24.8	24.9	0.64	0.64	0.64	0.100	0.137	0.127	0.076	0.074	0.224
5	26.1	26.0	26.1	0.66	0.66	0.66	0.064	0.087	0.081	0.095	0.094	0.283
6	28.1	28.1	28.4	0.69	0.69	0.69	0.042	0.058	0.054	0.137	0.137	0.429
7	30.2	30.5	29.8	0.72	0.72	0.71	0.029	0.040	0.037	0.196	0.206	0.545
8	32.0	31.9	32.4	0.74	0.74	0.74	0.021	0.029	0.026	0.261	0.257	0.823
9	33.9	34.1	33.7	0.76	0.76	0.75	0.015	0.021	0.020	0.347	0.357	1.000
10	34.8	34.4	–	0.76	0.76	–	0.012	0.016	0.015	0.395	0.373	–
11	32.5	35.2	–	0.74	0.74	–	0.009	0.012	–	0.282	0.282	–
12	42.0	42.0	–	0.82	0.82	–	0.007	0.009	–	1.000	1.000	–

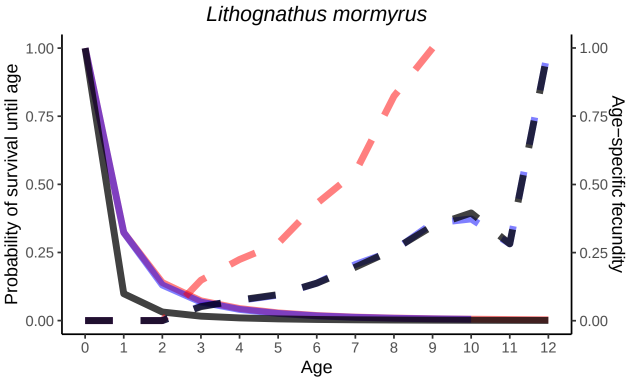

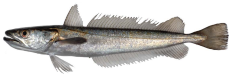

European hake

Merluccius merluccius

Parameter	Combined	Female	Male	Ref
L_{inf}	88.7	88.0	70	Piñeiro and Saínza[22]
K	0.128	0.127	0.184	
t_0	-1.174	-1.157	-0.973	
Maturity	3	3	4	
Max age	11	11	9	
$F = y(L)$	Power			Biagi et al.[3]
α	2.54			
β	3.07			

Age	L_x	$L_{x,f}$	$L_{x,m}$	l_x	$l_{x,f}$	$l_{x,m}$	Cum_c	Cum_f	Cum_m	B_x	$B_{x,f}$	$B_{x,m}$
1	20.6	20.9	21.0	0.32	0.33	0.33	1.000	1.000	1.000	0.000	0.000	0.000
2	29.0	28.4	29.5	0.50	0.50	0.51	0.319	0.334	0.326	0.000	0.000	0.000
3	36.7	37.0	36.4	0.62	0.63	0.61	0.161	0.167	0.167	0.000	0.000	0.000
4	43.8	44.5	42.7	0.69	0.70	0.68	0.099	0.105	0.102	0.192	0.000	0.352
5	50.0	48.7	45.7	0.74	0.73	0.71	0.069	0.074	0.069	0.288	0.235	0.434
6	55.4	53.7	49.7	0.77	0.77	0.74	0.051	0.054	0.049	0.395	0.318	0.561
7	58.3	56.4	54.2	0.79	0.78	0.76	0.039	0.041	0.036	0.461	0.370	0.732
8	63.1	62.3	56.6	0.81	0.81	0.78	0.031	0.032	0.027	0.588	0.502	0.836
9	67.1	68.7	60.0	0.82	0.83	0.79	0.025	0.026	0.021	0.711	0.677	1.000
10	75.0	75.0	–	0.85	0.85	–	0.020	0.022	0.017	1.000	0.887	–
11	74.0	78.0	–	0.85	0.86	–	0.017	0.018	–	0.960	1.000	–

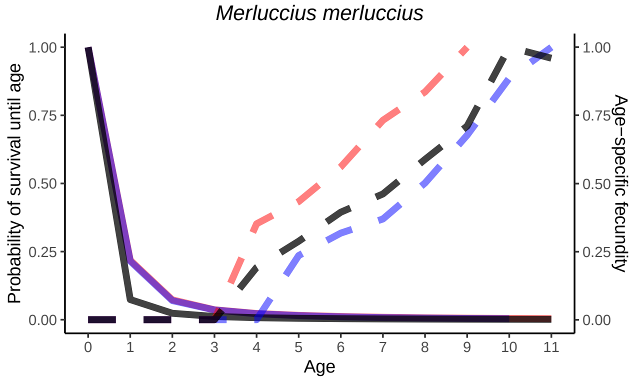

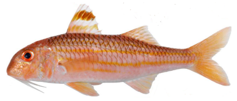

Striped red mullet

Mullus surmuletus

Parameter	Combined	Female	Male	Ref
L_{inf}	31.28	31.9	25.54	Reñones et al.[23]
K	0.211	0.205	0.273	
t_0	-2.348	-2.605	-2.45	
Maturity	2	1	2	
Max age	6	6	6	
$F = y(L)$	Power			Amin et al.[2]
α	0.0255			
β	5.031			

Age	L_x	$L_{x,f}$	$L_{x,m}$	l_x	$l_{x,f}$	$l_{x,m}$	Cum_c	Cum_f	Cum_m	B_x	$B_{x,f}$	$B_{x,m}$
1	15.9	16.7	15.7	0.56	0.58	0.57	1.000	1.000	1.000	0.000	0.000	0.146
2	18.6	19.5	17.8	0.63	0.65	0.62	0.559	0.582	0.567	0.000	0.000	0.272
3	21.3	21.9	19.8	0.69	0.70	0.67	0.352	0.379	0.354	0.203	0.157	0.465
4	23.1	23.6	21.5	0.72	0.72	0.70	0.242	0.264	0.237	0.304	0.227	0.717
5	24.6	25.3	22.0	0.74	0.75	0.71	0.174	0.191	0.167	0.419	0.320	0.800
6	29.3	31.7	23.0	0.79	0.81	0.73	0.128	0.143	0.118	1.000	1.000	1.000

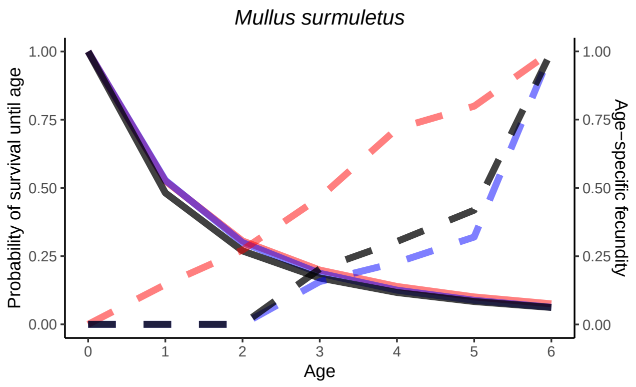

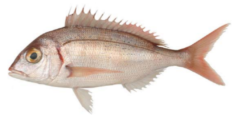

Common pandora

Pagellus erythrinus

Parameter	Combined	Female	Male	Ref
L_{inf}	38.29	35.41	40.01	Yapici and Filiz[29]
K	0.148	0.17	0.135	
t_0	-1.42	-1.32	-1.54	
Maturity	2	1	2	
Max age	8	8	8	
$F = y(L)$	Power			Papaconstantinou et al.[21]
α	1			
β	3.74			

Age	L_x	$L_{x,f}$	$L_{x,m}$	l_x	$l_{x,f}$	$l_{x,m}$	Cum_c	Cum_f	Cum_m	B_x	$B_{x,f}$	$B_{x,m}$
1	11.66	11.66	11.66	0.41	0.41	0.42	1.000	1.000	1.000	0.000	0.000	0.034
2	15.27	15.27	15.27	0.56	0.55	0.56	0.414	0.407	0.424	0.000	0.000	0.093
3	18.19	18.19	18.19	0.64	0.63	0.64	0.230	0.223	0.239	0.179	0.179	0.179
4	20.96	20.96	20.96	0.69	0.69	0.70	0.147	0.141	0.154	0.304	0.304	0.304
5	24.07	24.07	24.07	0.74	0.74	0.75	0.102	0.097	0.108	0.511	0.511	0.511
6	25.89	25.89	25.89	0.77	0.76	0.77	0.076	0.071	0.081	0.671	0.671	0.671
7	27.19	27.19	27.19	0.78	0.78	0.79	0.058	0.054	0.062	0.805	0.805	0.805
8	28.81	28.81	28.81	0.80	0.79	0.80	0.045	0.042	0.049	1.000	1.000	1.000

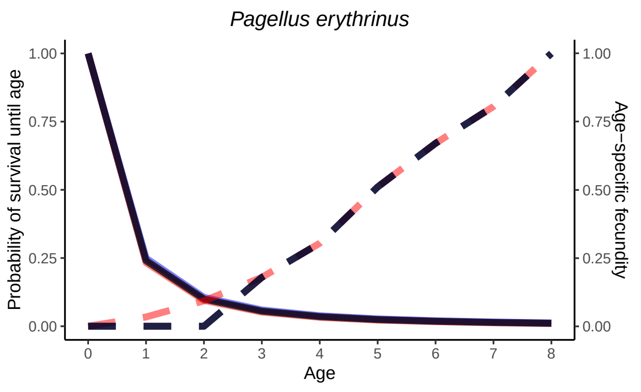

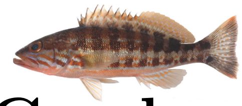

Comber

Serranus cabrilla

Parameter	Combined	Female	Male	Ref
L_{inf}	23.88	23.88	23.88	Uçkun İlhan et al.[27]
K	0.298	0.298	0.298	
t_0	-1.577	-1.577	-1.577	
Maturity	2	2	2	
Max age	6	6	6	
$F = y(L)$	Exponential			Palacios Sartagal[20]
α	72.46			
β	0.22			

Age	L_x	$L_{x,f}$	$L_{x,m}$	l_x	$l_{x,f}$	$l_{x,m}$	Cum_c	Cum_f	Cum_m	B_x	$B_{x,f}$	$B_{x,m}$
1	12.79	12.79	12.79	0.47	0.47	0.47	1.000	1.000	1.000	0.000	0.000	0.000
2	15.84	15.84	15.84	0.58	0.58	0.58	0.468	0.468	0.468	0.000	0.000	0.000
3	17.61	17.61	17.61	0.62	0.62	0.62	0.269	0.269	0.269	0.432	0.432	0.432
4	19.20	19.20	19.20	0.66	0.66	0.66	0.168	0.168	0.168	0.614	0.614	0.614
5	20.61	20.61	20.61	0.69	0.69	0.69	0.111	0.111	0.111	0.837	0.837	0.837
6	21.42	21.42	21.42	0.70	0.70	0.70	0.077	0.077	0.077	1.000	1.000	1.000

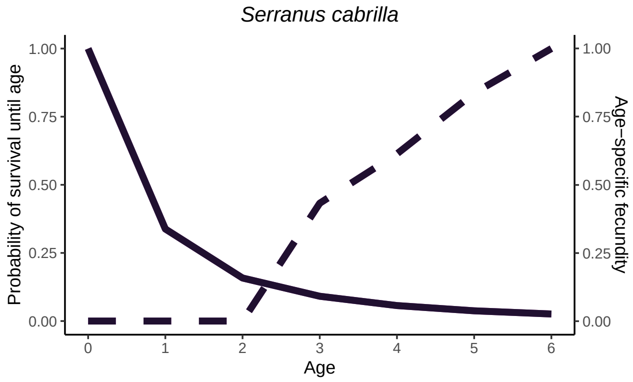

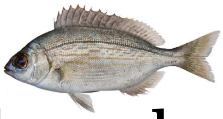

Black seabream

Spondyliosoma cantharus

Parameter	Combined	Female	Male	Ref
L_{inf}	43.35	41.92	45.89	Pajuelo and Lorenzo[19]
K	0.24	0.25	0.2	
t_0	-0.11	-0.29	-078	
Maturity	2	3	2	
Max age	10	10	10	
$F = y(L)$	Power			Gonçalves and Erzini[12]
α	436.27			
β	1.5747			

Age	L_x	$L_{x,f}$	$L_{x,m}$	l_x	$l_{x,f}$	$l_{x,m}$	Cum_c	Cum_f	Cum_m	B_x	$B_{x,f}$	$B_{x,m}$
1	10.6	13.75	10.14	0.14	0.26	0.15	1.000	1.000	1.000	0.000	0.000	0.000
2	17.3	19.57	17.22	0.39	0.46	0.42	0.137	0.264	0.146	0.000	0.000	0.000
3	23.5	24.34	22.80	0.55	0.57	0.56	0.053	0.121	0.061	0.443	0.447	0.000
4	27.9	28.25	27.18	0.63	0.64	0.64	0.029	0.069	0.035	0.581	0.565	0.555
5	31.4	31.45	30.63	0.68	0.68	0.69	0.018	0.044	0.022	0.699	0.669	0.669
6	34.5	34.06	33.35	0.71	0.71	0.72	0.012	0.030	0.015	0.811	0.759	0.765
7	36.5	36.21	35.48	0.73	0.73	0.75	0.009	0.021	0.011	0.887	0.836	0.844
8	38.2	37.96	37.61	0.75	0.75	0.76	0.006	0.015	0.008	0.952	0.900	0.908
9	38.9	39.40	38.48	0.75	0.76	0.77	0.005	0.012	0.006	0.980	0.955	0.959
10	39.4	40.58	39.52	0.76	0.77	0.78	0.004	0.009	0.005	1.000	1.000	1.000

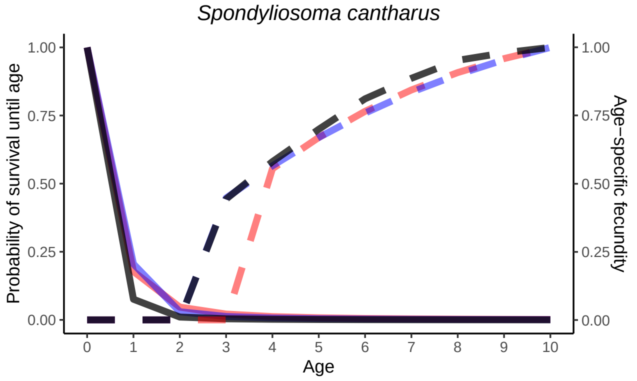

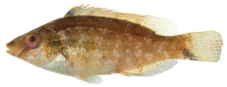

Grey wrasse

Symphodus cinereus

Parameter	Combined	Female	Male	Ref
L_{inf}	10.61	9.6	11.62	Kara and Quignard[15]
K	0.483	0.483	0.485	
t_0	-0.74	-0.74	0.44	
Maturity	1	1	1	
Max age	6	6	6	
$F = y(L)$	Exponential			Kara and Quignard[15]
α	2659			
β	0.0619			

Age	L_x	$L_{x,f}$	$L_{x,m}$	l_x	$l_{x,f}$	$l_{x,m}$	Cum_c	Cum_f	Cum_m	B_x	$B_{x,f}$	$B_{x,m}$
1	7.79	7.04	6.17	0.46	0.46	0.29	1.000	1.000	1.000	0.853	0.866	0.735
2	8.87	8.02	8.26	0.53	0.53	0.45	0.464	0.463	0.286	0.912	0.920	0.837
3	9.53	8.63	9.55	0.57	0.57	0.52	0.247	0.246	0.127	0.950	0.955	0.906
4	9.95	9.00	10.35	0.59	0.59	0.56	0.140	0.140	0.066	0.975	0.977	0.952
5	10.20	9.23	10.84	0.60	0.60	0.58	0.082	0.082	0.037	0.990	0.991	0.982
6	10.36	9.37	11.14	0.61	0.61	0.60	0.049	0.049	0.022	1.000	1.000	1.000

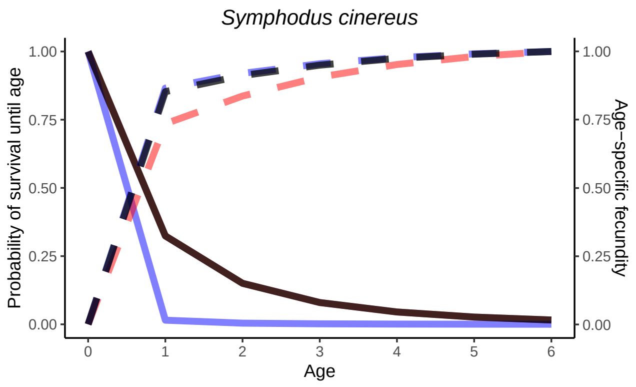

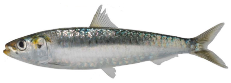

European pilchard

Sardina pilchardus

Parameter	Combined	Female	Male	Ref
L_{inf}	18.02	18.02	18.02	Alemany and Alvarez[1]
K	0.65	0.65	0.65	
t_0	-0.67	-0.67	-0.67	
Maturity	1	1	1	
Max age	5	5	5	
$F = y(L)$	Power			Bouhali et al.[4]
α	6.858			
β	2.497			

Age	L_x	$L_{x,f}$	$L_{x,m}$	l_x	$l_{x,f}$	$l_{x,m}$	Cum_c	Cum_f	Cum_m	B_x	$B_{x,f}$	$B_{x,m}$
1	14.2	14.2	14.2	0.39	0.39	0.39	1.000	1.000	1.000	0.602	0.602	0.602
2	16.0	16.0	16.0	0.46	0.46	0.46	0.395	0.395	0.395	0.811	0.811	0.811
3	17.2	17.2	17.2	0.50	0.50	0.50	0.182	0.182	0.182	0.972	0.972	0.972
4	17.2	17.2	17.2	0.50	0.50	0.50	0.090	0.090	0.090	0.972	0.972	0.972
5	17.4	17.4	17.4	0.50	0.50	0.50	0.045	0.045	0.045	1.000	1.000	1.000

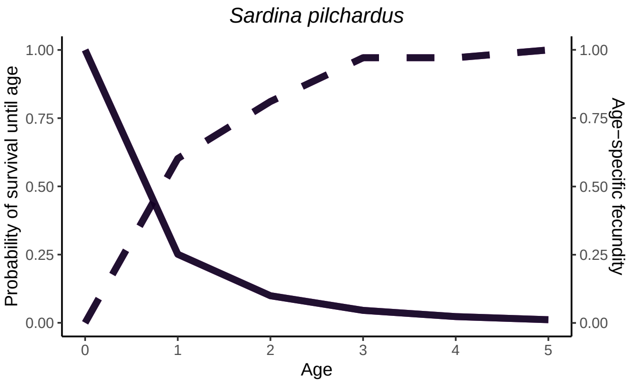

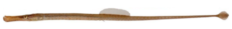

Broadnosed pipefish

Syngnathus typhle

Parameter	Combined	Female	Male	Ref
L_{inf}	26.2	26.2	26.2	Froese et al.[10]
K	0.558	0.558	0.558	
t_0	-0.5	-0.5	-0.5	
Maturity	1	1	1	
Max age	3	3	3	
$F = y(L)$	Exponential			Rispoli and Wilson[24]
α	6.7977			
β	0.1522			

Age	L_x	$L_{x,f}$	$L_{x,m}$	l_x	$l_{x,f}$	$l_{x,m}$	Cum_c	Cum_f	Cum_m	B_x	$B_{x,f}$	$B_{x,m}$
1	14.86	14.86	14.86	0.27	0.27	0.27	1.000	1.000	1.000	0.314	0.314	0.314
2	19.71	19.71	19.71	0.43	0.43	0.43	0.271	0.271	0.271	0.656	0.656	0.656
3	22.48	22.48	22.48	0.50	0.50	0.50	0.115	0.115	0.115	1.000	1.000	1.000

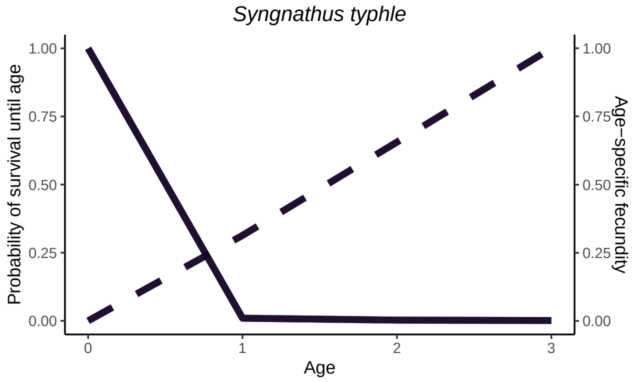

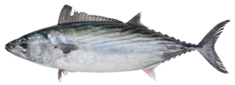

Atlantic bonito

Sarda sarda

Parameter	Combined	Female	Male	Ref
L_{inf}	80.87	80.87	80.87	Orsi Relini et al.[18]
K	0.352	0.352	0.352	
t_0	-1.7	-1.7	-1.7	
Maturity	1	1	1	
Max age	4	4	4	
$F = y(L)$	Power			Orsi Relini et al.[18]
α	0.01			
β	4.59			

Age	L_x	$L_{x,f}$	$L_{x,m}$	l_x	$l_{x,f}$	$l_{x,m}$	Cum_c	Cum_f	Cum_m	B_x	$B_{x,f}$	$B_{x,m}$
1	51.71	51.71	51.71	0.50	0.50	0.50	1.000	1.000	1.000	0.233	0.233	0.233
2	57.04	57.04	57.04	0.55	0.55	0.55	0.502	0.502	0.502	0.366	0.366	0.366
3	63.15	63.15	63.15	0.60	0.60	0.60	0.277	0.277	0.277	0.584	0.584	0.584
4	71.00	71.00	71.00	0.65	0.65	0.65	0.166	0.166	0.166	1.000	1.000	1.000

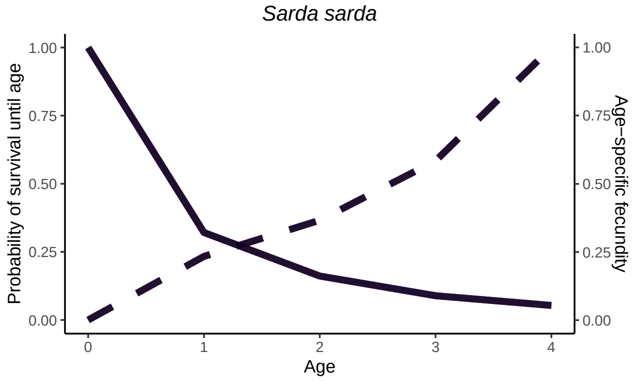

References

[1] Alemany, F. and Alvarez, F. (1993). Growth differences among sardine (Sardina pilchardus Walb.) populations in Western Mediterranean. *Sci. Mar.*, 57:229–234.

[2] Amin, A., Madkour, F., Abu El-Regal, M., and Moustafa, A. (2016). Reproductive biology of Mullus surmuletus (Linnaeus, 1758) from the Egyptian Mediterranean Sea (Port Said. *iNTERNATIONAL jOURNAL OF eNVIRONMENTAL sCIENCE and engineering*, 7:1–10.

[3] Biagi, F., Sbrana, M., Cesarini, A., and Viva, C. (1995). Reproductive biology and fecundity of Merlucciusmerluccius (Linnaeus, 1758) in the Northern Tyrrhenian sea.Dynamique des populationsmarines. *Cahiers Options Méditerranéennes*, (10):47–48.

[4] Bouhali, F., Lechekhab, S., Ladaimia, S., Assia, B., Amara, R., and Borhane, D. (2015). Reproduction et maturation des gonades de Sardina pilchardus dans le golfe d'Annaba (Nord-Est algérien). *Cybium: international journal of ichthyology.*

[5] Carrassón, M. and Bau, M. (2003). Reproduction, gonad histology and fecundity of *Aidablennius sphynx* (Pisces: Blenniidae) of the Catalan Sea (North-Western Mediterranean). *Scientia Marina*, 67(4):461–469.

[6] Colmenero, A. I., Tuset, V. M., Recasens, L., and Sanchez, P. (2013). Reproductive biology of Black Anglerfish (Lophius budegassa) in the northwestern Mediterranean Sea. *Fishery Bulletin*, 111(4):390–401.

[7] Curtis, J. M. R. and Vincent, A. C. J. (2006). Life history of an unusual marine fish: Survival, growth and movement patterns of Hippocampus guttulatus Cuvier 1829. *Journal of Fish Biology*, 68(3):707–733.

[8] Domínguez-Seoane, R., Pajuelo, J. G., Lorenzo, J. M., and Ramos, A. G. (2006). Age and growth of the sharpsnout seabream Diplodus puntazzo (Cetti, 1777) inhabiting the Canarian archipelago, estimated by reading otoliths and by backcalculation. *Fisheries Research*, 81(2):142–148.

[9] Faraj, E., Alssalam, A., Ali, S., Sayed, M., Sayed, E., Mor, E., Ali, R., Ali, S., Salem, E., and Alfergani, E. (2016). Reproductive Biology of the Striped Seabream Lithognathus mormyrus (Linnaeus, 1758) from Al Haneah Fishing Site, Mediterranean Sea, Eastern Libya. *Journal of Life Sciences*, 10.

[10] Froese, R., Pauly, D., and Editors (2000). FishBase 2000: Concepts, design and data sources. page 344.

[11] García-Rodríguez, M., Pereda, P., Landa, J., and Esteban, A. (2005). On the biology and growth of the anglerfish Lophius budegassa Spinola, 1807 in the Spanish Mediterranean: A preliminary approach. *Fisheries Research*, 71:197–208.

[12] Gonçalves, J. and Erzini, K. (2000). The reproductive biology of Spondyliosoma cantharus (L.) from the SW Coast of Portugal. *Scientia Marina*, 64:403–411.

[13] Iglésias, S. (2013). *Actinopterygians from the North-Eastern Atlantic and the Mediterranean (A Natural Classification Based on Collection Specimens, with DNA Barcodes and Standardized Photographs), Volume I (Plates), Provisional Version 09.*

[14] Kara, M. H. (1997). Cycle sexuel et fécondité du Loup Dicentrarchus labrax (Poisson Moronidé) du golfe d'Annaba. *Cahiers de Biologie Marine*, (3).

[15] Kara, M. H. and Quignard, J.-P. (2018). *Les poissons des lagunes et des estuaires de Méditerranée. 2, 2,.*

[16] Milton, P. (1983). Biology of littoral blenniid fishes on the coast of south-west England. *Journal of the Marine Biological Association of the United Kingdom*, 63(1):223–237.

[17] Monteiro, P., Bentes, L., Coelho, R., Correia, C., Erzini, K., Lino, P. G., Ribeiro, J., and Gonçalves, J. M. S. (2010). Age and growth, mortality and reproduction of the striped sea bream, Lithognathus mormyrus Linnaeus 1758, from the south coast of Portugal (Algarve). *Marine Biology Research*, 6(1):53–65.

[18] Orsi Relini, L., Garibaldi, F., Cima, C., Palandri, G., Lanteri, L., and Relini, M. (2005). Biology of atlantic bonito, Sarda sarda (Bloch, 1793), in the western and central mediterranean. A summary concerning a possible stock unit.

[19] Pajuelo, J. G. and Lorenzo, J. M. (1999). Life History of Black Seabream, Spondyliosoma cantharus, off the Canary Islands, Central-east Atlantic. *Environmental Biology of Fishes*, 54(3):325–336.

[20] Palacios Sartagal, N. (2017). *Estudi de La Fecunditat i Estratègia Reproductiva de Serranus Cabrilla (Pisces, Serranidae)*. PhD thesis, Universitat de Girona.

[21] Papaconstantinou, C., G.Petrakis, and Vassilopoulou, V. (1986). The fecundity of hake (Merluccius merluccius L.) and red pandora (Pagellus erythrinus L.) in the Greek seas. *Acta Adriatica*, 27:85–95.

[22] Piñeiro, C. and Saínza, M. (2003). Age estimation, growth and maturity of the European hake (Merluccius merluccius (Linnaeus, 1758)) from Iberian Atlantic waters. *ICES Journal of Marine Science*, 60(5):1086–1102.

[23] Reñones, O., Massutí, E., and Morales-Nin, B. (1995). Life history of the red mullet Mullus surmuletus from the bottom-trawl fishery off the Island of Majorca (north-west Mediterranean). *Marine Biology*, 123(3):411–419.

[24] Rispoli, V. F. and Wilson, A. B. (2008). Sexual size dimorphism predicts the frequency of multiple mating in the sex-role reversed pipefish Syngnathus typhle. *Journal of Evolutionary Biology*, 21(1):30–38.

[25] Škeljo, F. (2012). *Dinamika Populacije Kneza, Coris Julis (Linnaeus, 1758) u Istočnom Jadranu*. PhD thesis, University of Split.

[26] Taieb, A. H., Ghorbel, M., and Jarboui, O. (2013). Study of fecundity for Diplodus vulgaris (Teleost, Sparidae) in Gulf of Gabes. page 5.

[27] Uçkun İlhan, D., Akalın, S., tosunoğlu, Z., and Ozaydin, O. (2010). Growth Characteristics and Reproduction of Comber, Serranus Cabrilla (Actinopterygii, Perciformes, Serranidae), in the Aegean Sea. *Acta Ichthyologica Et Piscatoria*, 40:55–60.

[28] Wassef, E. A. and Emary, H. (1989). Contribution to the biology of bass, Dicentrarchus labrax L. in the Egyptian Mediterranean waters off Alexandria. /paper/Contribution-to-the-biology-of-bass%2C-Dicentrarchus-Wassef-Emary/335360d91c9f3d088051211b1da6b92b41cfb5fa.

[29] Yapici, S. and Filiz, H. (2019). Biological aspects of two coexisting native and non-native fish species in the Aegean Sea: Pagellus erythrinus vs. Nemipterus randalli. *Mediterranean Marine Science*, 20(3):594–602.
